# NMR-Guided Discovery of Luvunga D: A Novel Propellane-Type Limonoid from *Luvunga scandens* That Functions as a Non-Classical Ferroptosis Inhibitor

**DOI:** 10.3390/antiox15030402

**Published:** 2026-03-23

**Authors:** Bien-Thuy Bui Nguyen, Hoang-Minh Bui, Chia-Ching Liaw, Quoc-Dung Tran Huynh, Chih-Hua Chao, Duy-Hien Tran, I-Wen Lo, Thanh-Hoa Vo, Andreas Koeberle, Solveigh C. Koeberle, Mei-Chuan Chen, Yu-Chi Lin

**Affiliations:** 1 Ph.D. Program in Clinical Drug Development of Herbal Medicine, College of Pharmacy, Taipei Medical University, Taipei 110301, Taiwan; bnbthuy@uhsvnu.edu.vn (B.-T.B.N.); tranduyhien@ump.edu.vn (D.-H.T.); 2 National Research Institute of Chinese Medicine, Taipei 112026, Taiwan; liawcc@nricm.edu.tw (C.-C.L.); iwenlo99@nricm.edu.tw (I.-W.L.); 3 Department of Pharmacognosy, Faculty of Pharmacy, University of Health Sciences, Vietnam National University Ho Chi Minh City, Ho Chi Minh City 700000, Vietnam; vthoa@uhsvnu.edu.vn; 4 Michael Popp Institute, Center for Molecular Biosciences Innsbruck (CMBI), University of Innsbruck, 6020 Innsbruck, Austria; minh.bui-hoang@student.uibk.ac.at (H.-M.B.); andreas.koeberle@uni-graz.at (A.K.); 5 Unit of Pharmacognosy, Institute of Pharmacy, Center for Molecular Biosciences Innsbruck (CMBI), University of Innsbruck, 6020 Innsbruck, Austria; 6 Department of Pharmacy, School of Pharmaceutical Sciences, National Yang Ming Chiao Tung University, Taipei 112304, Taiwan; 7 Graduate Institute of Natural Products, Kaohsiung Medical University, Kaohsiung 807378, Taiwan; 8 Institute of Biological Chemistry, Academia Sinica, Taipei 115201, Taiwan; htqdung3012@as.edu.tw; 9 School of Pharmacy, China Medical University, Taichung 406040, Taiwan; chchao@mail.cmu.edu.tw; 10 Chinese Medicine Research and Development Center, China Medical University Hospital, Taichung 404327, Taiwan; 11 Department of Pharmacognosy–Traditional Pharmacy, School of Pharmacy, University of Medicine and Pharmacy at Ho Chi Minh City, Ho Chi Minh City 700000, Vietnam; 12 Research Center for Discovery and Development of Healthcare Products, Vietnam National University Ho Chi Minh City, Ho Chi Minh City 700000, Vietnam; 13 Institute of Pharmaceutical Sciences, Pharmacognosy and Excellence Field BioHealth, NAWI Graz, University of Graz, 8010 Graz, Austria; solveigh.koeberle@uni-graz.at; 14 School of Pharmacy, College of Pharmacy, Taipei Medical University, Taipei 110301, Taiwan; 15 Traditional Herbal Medicine Research Center, Taipei Medical University Hospital, Taipei 110301, Taiwan

**Keywords:** *Luvunga scandens*, NMR-guided, limonoid, ferroptosis, antioxidant reactions

## Abstract

Recent phytochemical investigations have demonstrated that *Luvunga scandens* is a rich source of structurally diverse secondary metabolites; however, its potential antioxidant-active constituents and their underlying mechanisms remain largely unexplored. In this study, an NMR-guided fractionation strategy applied to the rhizomes and leaves of *L. scandens* led to the isolation of ten limonoids, including three new compounds, Luvungas B–D (**3**, **4**, and **8**). Their structures and absolute configurations were determined through extensive spectroscopic analysis, X-ray diffraction, and ECD calculations. Based on the isolated analogues, a biosynthetic pathway is proposed, featuring the metabolic bifurcation of a key acyclic intermediate into the isoobacunoic acid and propellane-type lineages. Biological evaluation revealed that **8** inhibits RSL3-induced ferroptosis in HepaRG liver cells with an EC_50_ of 16.1 µM. Mechanistic studies demonstrated that, unlike classical antioxidants, compound **8** mitigates lipid peroxidation without exhibiting direct radical-scavenging or iron-chelating activities. These findings suggest that **8** suppresses ferroptosis via non-canonical mechanisms.

## 1. Introduction

Ferroptosis is an iron-dependent regulated-cell-death process driven by excessive accumulation of lipid peroxides. Distinct from apoptosis and necrosis, ferroptosis is driven by the collapse of cellular antioxidant defense mechanisms, particularly the glutathione-dependent lipid peroxide repair pathways [1]. Given its critical role in various pathologies, including neurodegenerative diseases, liver injury, and ischemia–reperfusion damage, the discovery of small molecules capable of mitigating ferroptotic cell death has become a priority in drug development. While synthetic antioxidants have shown promise, natural products—with their immense structural diversity—remain an underutilized resource for identifying compounds with novel scaffolds that may operate via distinct anti-ferroptotic mechanisms.

*Luvunga scandens* (Roxb.) Buch.-Ham. ex Wight & Arn. (Rutaceae) is a climbing shrub indigenous to Southeast Asia. The plant is a known source of limonoids, a class of highly oxygenated, modified triterpenoids derived from the mevalonate pathway [2,3,4]. Limonoids are recognized for a broad spectrum of pharmacological activities, such as anticancer, anti-inflammatory, and antimicrobial effects [3,5,6,7,8]. However, despite their chemical richness, the potential of *L. scandens*-derived limonoids in modulating ferroptosis has not been systematically investigated. Moreover, previous phytochemical studies on this species have largely relied on conventional isolation techniques, which often fail to capture minor or structurally complex metabolites, leaving a significant portion of its chemical space unexplored.

To overcome this limitation, advanced targeted isolation strategies are required. Nuclear Magnetic Resonance (NMR) spectroscopy is a definitive tool for structural elucidation; by comparing characteristic signals against accumulated research data, we can identify specific structural scaffolds during the isolation process, enabling targeted purification. This strategy complements conventional bioassay-guided fractionation by enabling the detection of structurally unique or minor metabolites that might otherwise be missed due to low abundance during preliminary screening, thereby ensuring these potential candidates are available for subsequent pharmacological evaluation.

**Figure 1:**
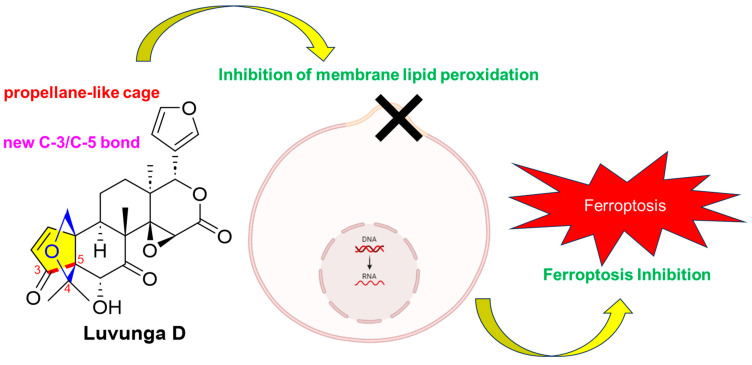
Graphical summary of Luvunga D and its inhibition of ferroptosis.

In this study, fractions displaying characteristic limonoid-type NMR signals were prioritized during the fractionation of *L. scandens* to investigate its limonoid diversity. The goal was to isolate structurally unusual limonoids, including potential new analogues, and evaluate their biological activity against ferroptosis in hepatic cells (Figure 1). Furthermore, we aimed to elucidate the biosynthetic relationships among the isolated congeners to provide insights into the metabolic diversification of limonoids in this species.

## 2. Materials and Methods

### 2.1. General

Melting points were measured on a MP(A)-J3 melting point apparatus (Yanaco, Kyoto, Japan). Optical rotations were determined using a JASCO P-2000 digital polarimeter (JASCO, Tokyo, Japan). UV spectra were recorded on a Shimadzu UV-2450 spectrophotometer (Shimadzu, Tokyo, Japan), and IR spectra were acquired using a Thermo Scientific Nicolet iS5 FT-IR spectrometer (Thermo Scientific, Madison, WI, USA). Circular dichroism spectra were measured on a JASCO J-815 CD spectropolarimeter (JASCO, Tokyo, Japan).

NMR spectra were recorded on a Bruker ARX-600 spectrometer (Bruker, Karlsruhe, Germany) with the residual solvent signals of chloroform-*d* (*δ*_H_ 7.26, *δ*_C_ 77.01) and methanol-*d*_4_ (*δ*_H_ 3.31, *δ*_C_ 49.0) as internal standard. HR-ESI-MS data were obtained using a Shimadzu LC-40D XSi LC system coupled with an LCMS-9030 Q-TOF mass spectrometer (Shimadzu, Tokyo, Japan). Single-crystal X-ray diffraction data were collected on a Bruker D8 VENTURE (Bruker, Karlsruhe, Germany) diffractometer equipped with a CuKα radiation source.

Semipreparative HPLC was performed on a Shimadzu LC-40D pump equipped with an SPD-20A UV detector using a Cosmosil C_18_ column (5 μm, 20 × 250 mm; Nacalai Tesque Inc., Kyoto, Japan). Column chromatography was carried out using silica gel (40–75 μm; Fuji Silysia Chemical Ltd., Kasugai, Japan), and thin-layer chromatography was performed on silica gel plates (Merck, Darmstadt, Germany).

### 2.2. Plant Materials

The rhizomes and the leaves of *L. scandens* were collected from Dong Nai province, Vietnam. The sample was identified by Dr. Thanh-Hoa Vo from the College of Pharmacy, University of Health Sciences, Vietnam National University Ho Chi Minh City, Ho Chi Minh City, Vietnam. The voucher specimens of the rhizomes (LS-20231101) and the leaves (LS-20231102) were deposited at the Herbarium of National Research Institute of Chinese Medicine, Taipei, Taiwan.

### 2.3. Extraction and Isolation

The dried rhizomes of *L. scandens* (3.5 kg) were extracted with 95% ethanol (20 L) under reflux for 24 h in triplicate, followed by extraction of the residue with 50% ethanol (20 L) under the same conditions. The combined EtOH extracts were concentrated under reduced pressure to afford a crude residue, which was suspended in H_2_O and subsequently sequentially partitioned with *n*-hexane, ethyl acetate, and *n*-butanol. The ethyl acetate layer (100 g) was subjected to flash column chromatography on silica gel (40–75 μm), eluted with a gradient of *n*-hexane–ethyl acetate–methanol (from 9:1:0 to 0:5:5, *v*/*v*/*v*), to afford eight fractions (LSR-B1–LSR-B8). Fractions LSR-B4 and LSR-B5 were identified as limonoid-rich based on the characteristic signals observed in their ^1^H and ^13^C NMR spectra. Compounds **1** (20.2 mg, *t*_R_ = 54 min), **2** (1.0 g, *t*_R_ = 56 min), and **3** (11.5 mg, *t*_R_ = 63 min) were obtained from fraction LSR-B4 (4.0 g) by semipreparative HPLC on an ODS column, eluted with 45% acetonitrile at a flow rate of 6.0 mL/min. Fraction LSR-B5 (5.0 g) was subsequently separated by silica gel (40–75 μm) open-column chromatography using dichloromethane–methanol (9:1, *v*/*v*) to afford seven subfractions (LSR-B5-1–LSR-B5-7). Subfraction LSR-B5-3 was further purified by semipreparative HPLC on an ODS column eluted with 40% acetonitrile (flow rate: 6.0 mL/min) to yield compounds **4** (294.7 mg, *t*_R_ = 42 min) and **5** (34.3 mg, *t*_R_ = 48 min). Compounds **6** (7.5 mg, *t*_R_ = 35 min) and **7** (12.3 mg, *t*_R_ = 45 min) were obtained from subfraction LSR-B5-4 under the same isolation conditions.

The dried leaves of *L. scandens* (7.0 kg) were extracted with 95% EtOH (40 L) under reflux. The combined extracts were concentrated under reduced pressure to yield a crude residue, which was suspended in H_2_O and successively partitioned with *n*-hexane to remove chlorophyll, followed by partitioning with ethyl acetate and *n*-butanol. The ethyl acetate layer was subjected to silica gel column chromatography (40–75 μm), eluted with a gradient of *n*-hexane–ethyl acetate–methanol (from 9:1:0 to 0:5:5, *v*/*v*/*v*), to afford 15 fractions (LSL-B1–LSL-B15). Fraction LSL-B2 (1.0 g) exhibited NMR features indicative of limonoids and was further separated by silica gel (40–75 μm) open-column chromatography using dichloromethane–methanol (95:5, *v*/*v*) to afford ten subfractions (LSL-B2-1–LSL-B2-10). Compounds **8** (5.2 mg, *t*_R_ = 44 min), **9** (7.6 mg, *t*_R_ = 49 min), and **10** (5.1 mg, *t*_R_ = 60 min) were obtained from subfraction LSL-B2-6 by semipreparative HPLC on an ODS column eluted with 40% acetonitrile at a flow rate of 7.0 mL/min.

Luvunga B (**3**): White amorphous powder; UV (MeOH) *λ_max_* 205 nm; ${\left[\alpha \right]}_{D}^{25}$ −49.6 (*c* 0.3, MeOH); ECD (MeOH): 305 (Δε −9.52) nm; IR (KBr) *ʋ_max_* 2954, 1746, 1445, 1170, 1119, 1069, 876, 805 cm^−1^; ^1^H- and ^13^C-NMR data (600 MHz/150 MHz, chloroform-*d*), see Table 1; positive HRESIMS: *m*/*z* 531.2222 ([M + H]^+^ calcd. for C_28_H_35_O_10_).

Luvunga C (**4**): colourless needles; UV (MeOH) *λ_max_* 205 nm; mp 135 °C; ${\left[\alpha \right]}_{D}^{25}$ −31.2 (*c* 0.3, MeOH); ECD (MeOH): 297 (Δε −9.71) nm, 234 (Δε −20.16) nm; IR (KBr) *ʋ_max_* 3456, 3147, 2962, 2559, 1726, 1277, 1195, 874, 736 cm^−1^; ^1^H- and ^13^C-NMR data (600 MHz/150 MHz, methanol-*d*_4_), see Table 2; positive HRESIMS: *m*/*z* 503.2254 ([M + H]^+^ calcd. for C_27_H_35_O_9_).

Luvunga D (**8**): White amorphous powder; UV (MeOH) *λ_max_* 212 nm; ${\left[\alpha \right]}_{D}^{25}$ +34.5 (*c* 0.3, MeOH); ECD (MeOH): 321 (Δε +6.97) nm, 239 (Δε −4.27) nm; IR (KBr) *ʋ_max_* 3400, 1744, 1445, 1289, 1165, 1116, 1067, 879, 602 cm^−1^; ^1^H- and ^13^C-NMR data (600 MHz/150 MHz, methanol-*d*_4_), see Table 2; positive HRESIMS: *m*/*z* 469.1832 ([M + H]^+^ calcd. for C_26_H_29_O_8_).

### 2.4. X-Ray Crystallographic Analysis of Compound **4**

The colorless crystals of compound **4** were obtained via natural evaporation from methanol solution. Crystal data were collected using a Bruker D8 VENTURE diffractometer (Bruker, Karlsruhe, Germany) with kappa geometry, a IµS DIAMOND microfocus X-ray tube (Incoatec GmbH, Geesthacht, Germany), a Photon-III C28 area detector (Bruker, Karlsruhe, Germany) and an Oxford Cryosystems low temperature device (Oxford Cryosystem, Oxford, UK). Data were corrected for absorption effects using the multi-scan method with SADABS (Version 2016/2). The space groups were assigned using XPREP within the Bruker APEX5 suite of programs, and the structures were solved using SHELXT (Version 2018/2) and refined using SHELXL (Version 2019/3), with the graphical interface Olex2 (Version 1.5).

Crystallographic data on **4**:

Crystal system: Orthorhombic, empirical formula: C_27_H_34_O_9_ (M = 502.54 g/mol), space group P2**_1_**2**_1_**2**_1_**, *a* = 7.8196(2) Å, *b* = 10.6277(3) Å, *c* = 28.4548(8) Å, α = 90°, β = 90°, γ = 90°, Volume = 2364.72(11) Å^3^, Z = 4, Density (calculated) = 1.412 Mg/m^3^, Wavelength = 1.54178 Å, theta range for Cell parameters = 3.11 to 72.11°, absorption coefficient = 0.877 mm^−1^. Crystal size: 0.150 × 0.150 × 0.100 mm^3^. Independent reflections: 4641 [R(int) = 0.0448]. Final R indices [I > 2sigma(I)]: R1 = 0.0310, wR2 = 0.0816. R indices (all data): R1 = 0.0311, wR2 = 0.0818. Absolute structure parameter = 0.00(5). CCDC number: 2424723.

### 2.5. ECD Calculations

The GMMX Plugin in GaussView 6 was used for conformational search using MMFF94 force field with an energy cutoff of 6 kcal/mol. The geometry optimizations and frequency calculations for the resulting conformers were performed using Gaussian 16 [9] at the M062X/def2tzvp level of theory with SMD solvation mode in MeOH. The time-dependent density functional theory (TDDFT) calculations were performed at the same level of theory as the preceding geometry optimization. The final ECD curve was simulated by aggregating the contributions of individual conformations, with each spectrum scaled by its respective Boltzmann population. The final ECD spectra were generated using the SpecDis (Version 1.71) program based on the Boltzmann-weighted contribution of each conformer.

### 2.6. Cell Culture

Human HepaRG liver cells were obtained from Biopredic International, (Rennes, France), (#HPR101), and were routinely maintained at 37 °C in a 5% CO_2_ atmosphere. The cells were passaged every 3–5 days at a confluence of 60–80% via trypsination (trypsin-EDTA, Merck, #59418C) and used from passage 18 to 40. Within this passage range, HepaRG cells consistently exhibit stable growth characteristics and reproducible experimental outcomes, with no detectable passage-dependent differences in the measured endpoints. Cells were cultured in William’s E medium (Sigma-Aldrich, Vienna, Austria, W4128) supplemented with 10% fetal bovine serum (FBS, Sigma-Aldrich, #0001662318), 2 mM L-glutamine (Sigma-Aldrich, R8758), 5 µg/mL human insulin (Sigma-Aldrich, I9278), and 37 (Figure 10B,C) or 50 µM hydrocortisone 21-hemisuccinate (Figure 10A) (Cayman, Ann Arbor, MI, USA, H2270).

### 2.7. Cell Viability by MTT Assay

HepaRG cells were seeded at 10,000 cells per well in 96-well plates. Following 24 h of incubation at 37 °C with 5% CO_2_, cells were exposed to either the vehicle (0.5% DMSO) or the test compounds (**3**, **4**, and **8**) for 48 h alone or in combination with cell death inducer RLS3 (Cayman, Cay19288; 0.3 µM). Cell viability was determined by monitoring the reduction of 3-(4,5-dimethylthiazol-2-yl)-2,5-diphenyltetrazolium bromide (MTT) by cellular dehydrogenases [10]. After 48 h treatment, MTT solution (Sigma Aldrich, M2128; 5 mg/mL in phosphate-buffered saline (PBS) pH 7.4, sterile filtered) was added to each well and the incubation continued for 3 h at 37 °C and 5% CO_2_. To dissolve the formazan crystals, 100 µL of sodium dodecylsulphate solution (SDS 10% in 20 mM HCl, pH 4.5) was added to each well, followed by incubation for 16 h in the dark on a POS 300 orbital shaker (Grantbio, Shepreth, UK at 60–80 rpm. Absorbance was measured at 570 nm using a multi-mode microplate reader SpectraMAX iD3 spectrometer (Molecular Devices, San José, CA, USA) or a Hidex Sense microplate reader (Hidex, Turku, Finland). Normalization was performed to vehicle (set as 100% cell viability) after subtracting the background signal from the ethanol controls. Data were fitted by nonlinear regression using a four-parameter logistic model (log[inhibitor] vs. response, variable slope) to determine EC_50_ values using Prism 10.4 (GraphPad, Boston, MA, USA). The EC_50_ was defined as the concentration of compound **8** that achieves half-maximal inhibition of RSL3-induced cytotoxicity.

### 2.8. DPPH Assay

The DPPH assay was performed by mixing 100 µL of compound **8** at varying concentrations (final concentrations: 1, 3, 10, 30, and 50 µM in ethanol) with 100 µL of 0.1 mM ethanolic DPPH solution [11]. After incubation for 30 min in the dark on a POS 300 orbital shaker (Grantbio, The Netherlands) at 130 rpm, the absorbance was measured at 520 nm using a Hidex Sense microplate reader. The radical scavenging activity (% DPPH scavenging) was calculated as: % DPPH scavenging = (1 − A_sample_)/A_0_ × 100. A_sample_: absorbance of the sample, A_0_: absorbance of the control (DPPH solution). Vitamin C (1 µM, 3 µM, 10 µM, 30 µM and 50 µM) was used as positive control.

### 2.9. Iron-Binding Capacity

To determine the iron-chelating activity, 5 µL of vehicle (DMSO) or test compound was mixed with 5 µL of 0.5 mM FeSO_4_ × 7 H_2_O in 40 µL HEPES buffer (50 mM, pH 3–6) at compound: Fe^2+^ ratios of 1:1, 2:1, 3:1, or 6:1 and incubated for 2 min [11]. 50 µL of 5 mM Ferrene (in 5 mM HEPES) was added, and the mixture was incubated for 2 min in the dark. Fe^2+^-Ferrene complex formation was measured at 595 nm using a Hidex Sense microplate reader (Hidex, Finland) [12]. Deferoxamine (DFO) was used as a positive control, and results are expressed as percentage of chelated ferrous iron.

### 2.10. FENIX Assay

The preparation of artificial membranes from egg phosphatidylcholine (PC) was performed as previously described [13]. Briefly, an appropriate amount of phosphatidylcholine (PC) was dissolved in a minimal volume of chloroform to ensure complete solubilization. The solvent was evaporated under a gentle argon stream, forming a uniform lipid film on the inner wall of the glass vial. Residual solvent was removed by maintaining the vial under argon for an additional 30 min. The dried lipid film was subsequently rehydrated with phosphate-buffered saline (PBS, pH 7.4) to obtain a 20 mM PC suspension. To form liposomes, the PC suspension was subjected to ten freeze–thaw cycles (4 min on ice, 4 min at room temperature), with 4 min sonication after each thawing (Sonorex Super Rx 2554, Berlin, Germany). The suspension was subsequently passed 20–25 times through a 100 nm polycarbonate membrane using a mini extruder (Avanti Research™—A Croda Brand, Alabaster, AL, USA). Liposome size and distribution were determined by DLS (Zetasizer Advance Ultra (Malvern Pananalytical, Almelo, The Netherlands); only preparations with a mean diameter of ~100 nm and a PDI of 0.2–0.3 were used. Liposomes were stored at 4 °C and used within two weeks. The extent of phospholipid peroxidation in artificial membranes was determined using a modified (FENIX) assay [14]. Briefly, egg PC liposomes (1 mM in PBS pH 7.4) were combined with STY-BODIPY (Cayman, #Cay27089, 1 µM) in a black 96-well polypropylene plate (Greiner, Kremsmuenster, Austria, #655087) and incubated in the presence of liproxstatin-1 (Lip-1, Fisher Scientific, Vienna, Austria, #16458017) or compound **8** (2 and 20 µM) for 10 min at 37 °C, followed by vigorous mixing for 5 min. Autoxidation was initiated by the addition of DTUN (Cayman, #Cay32742; 2 μM). Control wells contained 1% DMSO, 1 μM STY-BODIPY, and DTUN. After mixing for 5 min, the plate was equilibrated at 37 °C for 10 min before fluorescence (λ_Ex/Em_ = 485/535 nm) was time-dependently acquired for 500 min using a a Hidex Sense microplate reader (Hidex, Finland) in bottom-read mode. Fluorescence intensities of oxidized STY-BODIPY (λ_Ex/Em_ = 485/535 nm) at 500 min were used to quantify lipid peroxidation, and inhibition was calculated relative to the vehicle control (DMSO, 1%).

### 2.11. BODIPY 581/591 C11 Staining

HepaRG cells were seeded in 12-well plates at 2 × 10^5^ cells/well in growth medium and incubated for 24 h at 37 °C with 5% CO_2_. Compound **8** was added and cells incubated for 2 h at 37 °C with 5% CO_2_. Ferroptosis was induced with RSL3 (0.5 µM), and 2 h later, 2 µM C11-BODIPY (Cayman, #27086) was added for 30 min at 37 °C in the dark. Cells were washed with 500 µL PBS (pH 7.4), detached with 0.25% trypsin-EDTA, neutralized with growth medium, and collected along with washes in a 5 mL tube. Samples were centrifuged at 1200× *g* for 5 min at 4 °C and washed twice with HBSS. The pellet was resuspended in 500 µL HBSS, and flow cytometry data were acquired by collecting a total of 10,000 events within the gate defined for HepaRG cells on a CytoFLEX flow cytometer (Beckman, Vienna, Austria; λ_ex/em_ = 488/550 nm). Data were processed using FlowJo v10.10.0 with a consistent gating strategy, including ~10% C11-BODIPY-positive cells in the DMSO control and excluding debris. Mean fluorescence intensity was calculated as a percentage of the vehicle control.

### 2.12. Statistics

Data are shown as mean or individual values and means ± SEM from *n* independent experiments. Data processing and calculations were performed using Microsoft Excel (Version 2302, Microsoft 365 Apps for Enterprise, Redmond, WA, USA). Repeated measures one-way ANOVA was used, followed by Dunnett’s post-hoc tests. All statistical analyses were performed using GraphPad Prism 10, and differences were considered significant at *p* < 0.05.

## 3. Results and Discussion

Recent phytochemical investigations have demonstrated that *Luvunga scandens* is a rich source of structurally diverse secondary metabolites. Our recent work revealed that the rhizomes of this species are particularly enriched in bioactive monoterpene–coumarins, highlighting its considerable chemical diversity [15]. In addition to coumarin derivatives, previous studies have also reported the occurrence of limonoids and acridone alkaloids in *L. scandens*, indicating the presence of multiple biosynthetically distinct metabolite classes within this plant [16]. As part of our ongoing efforts to discover structurally novel natural products from *L. scandens*, the limonoid-type constituents of this species were further investigated in the present study.

### 3.1. NMR-Guided Isolation of Limonoid

**Figure 2:**
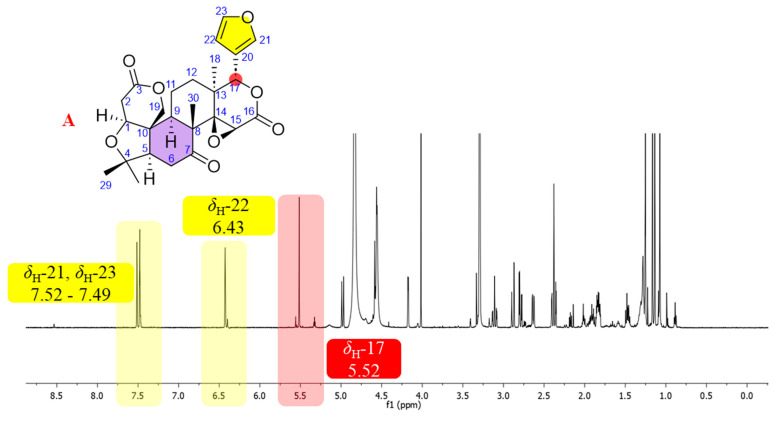

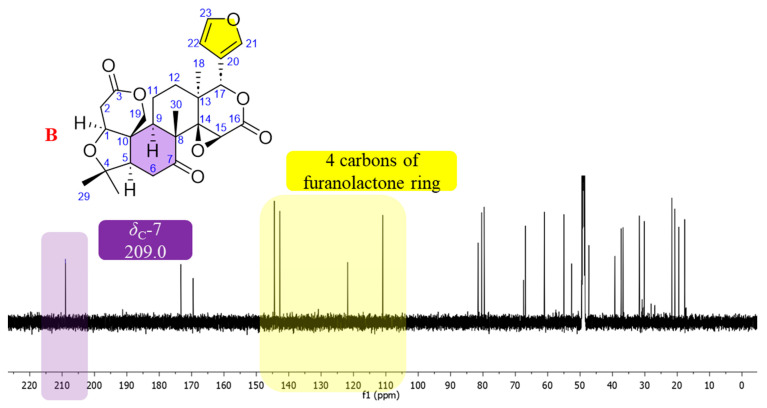
(**A**) The characteristic signals of limonin on ^1^H-NMR (600 MHz, Methanol–*d*_4_), (**B**) the characteristic signals of limonin on ^13^C-NMR (150 MHz, Methanol–*d*_4_). Colored backgrounds highlight specific structural assignments: the furan/furanolactone ring (yellow), H-17 (red), and C-7 (purple).

Among the limonoids, limonin is one of the most extensively studied members owing to its well-defined structural framework and biological properties. In the present study, limonin (**1**) was initially isolated from *L. scandens* and served as a reference compound for the identification of limonoid-type constituents [2,17]. The NMR spectroscopic features of limonin were consistent with those of a limonoid-type skeleton. A furan moiety was indicated by three downfield olefinic proton resonances at *δ*_H_ 7.52 (d, *J* = 0.8 Hz, H-21), 6.43 (dd, *J* = 2.0, 0.8 Hz, H-22), and 7.49 (t, *J* = 1.8 Hz, H-23), which displayed small coupling constants characteristic of five-membered heteroaromatic rings [18], together with four olefinic carbon signals at *δ*_C_ 121.8 (C, C-20), 142.7 (CH, C-21), 111.0 (CH, C-22), and 144.5 (CH, C-23). An oxymethine singlet proton resonance at *δ*_H_ 5.52 attributable to H-17 was consistent with the presence of a furanolactone ring system attached at C-17 (Figure 2A). Moreover, a characteristic downfield carbonyl carbon resonance at *δ*_C_ 209.0 was observed within the typical range reported for C-6 or C-7 ketone functionalities in limonoids (*δ*_C_ 205–215). According to previous reports, the presence of such a ketone moiety represents a diagnostic structural feature commonly associated with limonin-type and related limonoid subclasses (Figure 2B).

Based on these characteristic NMR signatures, limonin was employed as a diagnostic scaffold to guide the systematic profiling and tracking of limonoid-type constituents during the fractionation process. Accordingly, fractions and subfractions exhibiting similar diagnostic ^1^H and ^13^C NMR features were prioritized for further purification, facilitating the isolation of additional, structurally novel limonoids from this species.

**Figure 3:**
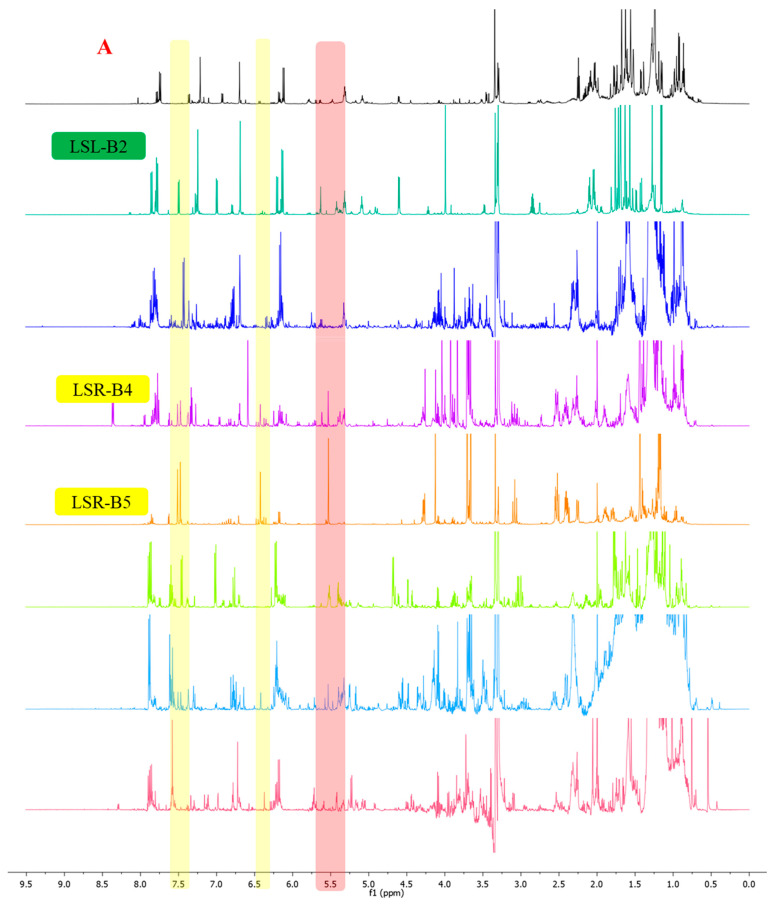

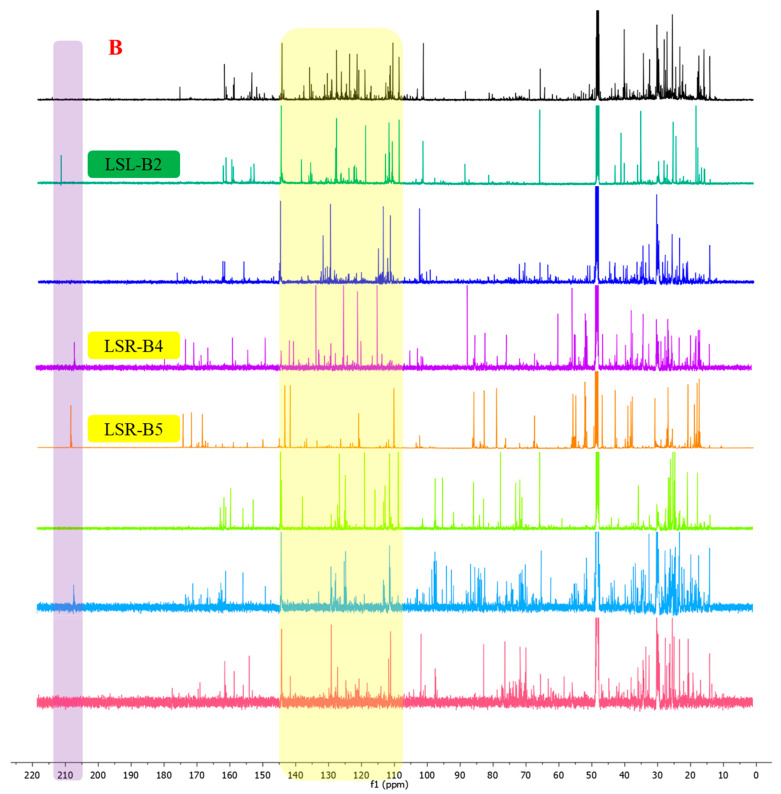
Representative NMR spectra of selected fractions from *Luvunga scandens*. (**A**) ^1^H-NMR (600 MHz, Methanol–*d*_4_) and (**B**) ^13^C-NMR (150 MHz, Methanol–*d*_4_). Different colored lines represent different selected fractions. The colored backgrounds highlight diagnostic signals characteristic of limonoid constituents: the furan ring (yellow), H-17 (red, in A), and C-7 (purple, in B).

**Figure 4:**
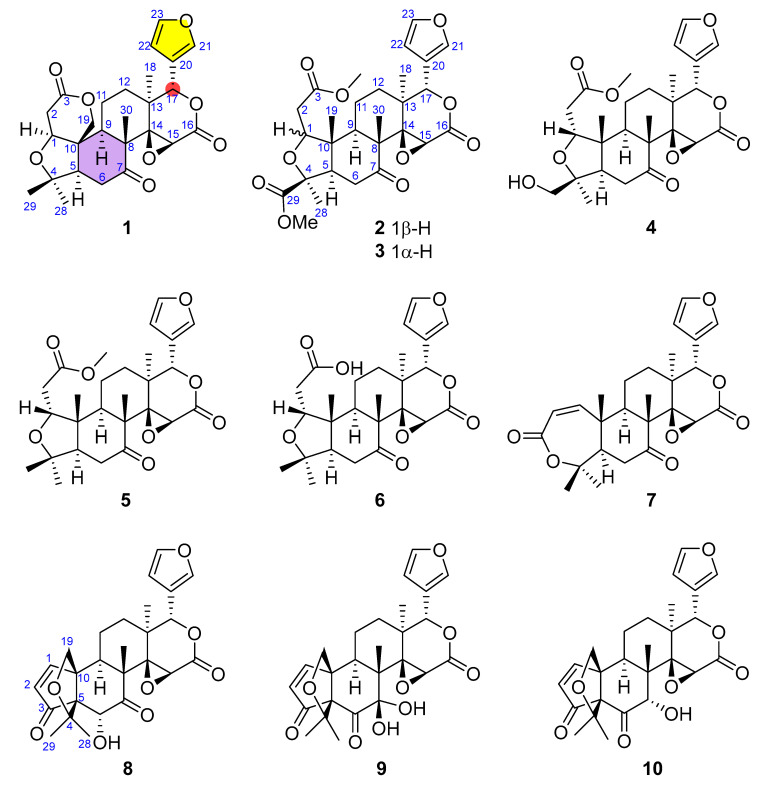
Structures of limonoids from *L. scandens.*

To access the limonoid constituents of *L. scandens* from both rhizomes and leaves, the crude extracts obtained separately from each plant part were subjected to successive liquid–liquid partitioning with *n*-hexane, ethyl acetate, and *n*-butanol. Based on the diagnostic NMR features defined above, limonoid-type constituents were predominantly enriched in the ethyl acetate layers. Subsequent silica gel column chromatography of the ethyl acetate layers afforded eight subfractions from the rhizomes (LSR-B1–LSR-B8) and fifteen subfractions from the leaves (LSL-B1–LSL-B15). Detailed inspection of the ^1^H and ^13^C NMR spectra of selected subfractions, including LSR-B4, LSR-B5, and LSL-B2 (Figure 3A,B), revealed prominent diagnostic resonances attributable to H-17, H-21, H-22, and H-23, together with olefinic carbon signals associated with C-21–C-23 and a carbonyl carbon resonance corresponding to a ketone functionality at C-6 or C-7. The relative intensities of these characteristic signals indicated that these subfractions contained limonoid-type constituents and were therefore prioritized for further purification and investigation. As a result, a total of ten limonoids were isolated from different parts of *L. scandens* through successive chromatographic separations (Figure 4).

### 3.2. Structure Elucidation of New Limonoids

Compound **3**

The molecular formula of compound **3** was established as C_28_H_34_O_10_ based on the protonated molecular ion peak at *m*/*z* 531.2222 [M + H]^+^, as determined by HR-ESI-MS (calcd. for C_28_H_35_O_10_, 531.2224). Analysis of the ^13^C NMR and DEPT spectra (Supplementary Information, Figures S5 and S6), in combination with the molecular formula, indicated twelve degrees of unsaturation.

The IR spectrum of compound **3** (Supplementary Information, Figure S3) exhibited a strong absorption band at 1746 cm^−1^, characteristic of carbonyl functionalities, while additional absorptions at 1445 cm^−1^ suggested the presence of olefinic groups. Furthermore, absorption bands observed at 1069 cm^−1^ were attributable to C–O stretching vibrations, consistent with oxygenated functionalities.

**Figure 5:**
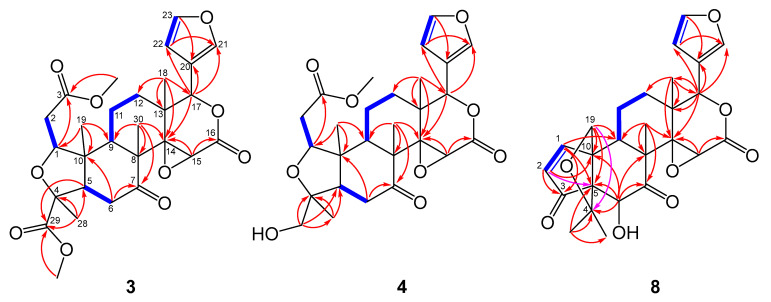
Key COSY correlations (blue bold bond) and HMBC correlations (arrow) of compound **3**, **4**, and **8,** with pink arrows highlighting the key HMBC correlations.

The ^1^H NMR spectrum (Table 1) displayed diagnostic signals of a β-substituted furan ring at δ_H_ 7.39 (H-21), 7.38 (H-23), and 6.31 (H-22), correlating with carbons at δ_C_ 141.1, 143.1, and 109.8, respectively. The elucidation of the polycyclic scaffold began with the fused B/C rings, guided by the angular methyl groups H_3_-30 and H_3_-18. The HMBC correlations from H_3_-30 (*δ*_H_ 1.19) to the ketone carbonyl C-7 (*δ*_C_ 207.8), C-8 (*δ*_C_ 50.9), C-9 (*δ*_C_ 46.2), and C-14 (*δ*_C_ 67.0) defined the B/C ring fusion and located the ketone functionality at C-7 (Figure 5). The construction of the B-ring was completed by the COSY correlation between H-5 (*δ*_H_ 2.05) and H-6 (*δ*_H_ 3.01, 2.62), which established the connectivity between the junction methine C-5 and the methylene C-6, linking the A/B junction to the ketone. Subsequently, the C-ring framework was outlined by HMBC correlations from H_3_-18 (*δ*_H_ 1.17) to C-12 (*δ*_C_ 29.3), C-13 (*δ*_C_ 38.6), C-14, and C-17 (*δ*_C_ 77.8). This central core was firmly consolidated by a continuous COSY spin system extending from H-9 (*δ*_H_ 2.32) through H-11 to H-12, which bridged the methine C-9 and the quaternary carbon C-13, completing the fused B/C skeleton. With the central core established, the furan moiety was positioned at C-17 based on the key HMBC correlations of H-17 (*δ*_H_ 5.49) with the furan carbons C-20 (*δ*_C_ 120.2), C-21 (*δ*_C_ 141.1), and C-22 (*δ*_C_ 109.8). Subsequently, the HMBC correlations from H-15 (*δ*_H_ 4.26) to C-14 and the ester carbon C-16 (*δ*_C_ 167.2) linked the C-ring to the carbonyl group (Figure 5). The presence of a 14,15-epoxide was then deduced from the characteristic chemical shifts of the quaternary C-14 (*δ*_C_ 67.0) and the methine C-15 (*δ*_C_ 54.7). Finally, the closure of the δ-lactone ring was confirmed by the significant downfield shift of H-17 and the oxymethine carbon C-17 (*δ*_C_ 77.8). The connectivity of the A-ring to the core was established by HMBC correlations from H_3_-19 (*δ*_H_ 0.98) to C-1 (*δ*_C_ 84.4), C-5, C-9, and C-10. HMBC correlations from H-2 to the ester carbonyl C-3 (*δ*_C_ 171.5) indicated a methoxycarbonylmethyl side chain at C-1. Conversely, the HMBC correlations from H_3_-28 (*δ*_H_ 1.45) to the quaternary carbon C-4 (*δ*_C_ 81.0) and the ester carbonyl C-29 (*δ*_C_ 173.6) confirmed the presence of a methyl group and a methoxycarbonyl group at C-4. Finally, to satisfy the twelfth degree of unsaturation required by the molecular formula, the distinct downfield shifts of C-1 (*δ*_C_ 84.4) and C-4 (*δ*_C_ 81.0) indicated the presence of an ether linkage between these positions, forming a tetrahydrofuran A-ring and completing the planar structure of compound **3**.

**Figure 6:**
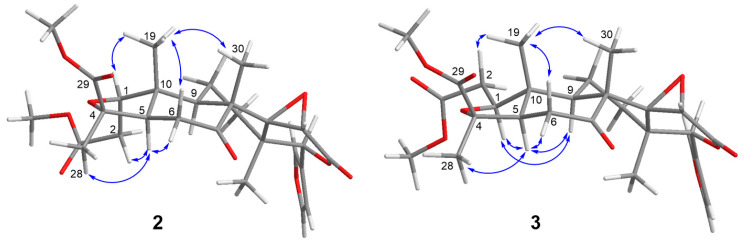
Key NOESY (blue arrow) of compounds **2** and **3**.

The relative configuration of compound **3** was deduced from its NOESY spectrum (Figure 6) and by comparison with the experimental data of Luvunga A (**2**). Assuming the ring junction proton H-5 adopts an *α*-orientation in both compounds, the stereochemistry of the tricyclic core was firmly established. In the NOESY spectrum of **3**, correlations were observed for H_3_-28 (*δ*_H_ 1.45)/H-5 (*δ*_H_ 2.05)/H-6α (*δ*_H_ 2.62), indicating that these protons are co-facial and *α*-oriented. Conversely, the correlations of H-6α (*δ*_H_ 3.01)/H_3_-19 (*δ*_H_ 0.98)/H_3_-30 (*δ*_H_ 1.19) placed these groups on the opposite face (*β*-orientation). These diagnostic signals are consistent with the conserved skeleton observed in our experimental data for **2**.

However, a distinct difference in the A-ring geometry was revealed by the specific NOE interactions involving H-1 and H-2. In our NOESY experiment for **2**, correlations were observed between H-1/H_3_-19 and H-2/H-5. In contrast, compound **3** exhibited a reversed pattern: H-1 (*δ*_H_ 4.04) showed a key correlation with H-5 (*α*-oriented), while H-2 (*δ*_H_ 2.64) correlated with the angular methyl H_3_-19 (*β*-oriented). This specific spatial arrangement implies that in compound **3**, H-1 is *α*-oriented, and H-2 is *β*-oriented, which is the opposite configuration at C-1 compared to **2**. This stereochemical assignment is strongly corroborated by the ^13^C NMR data regarding the angular methyl C-19 (Table 1, Supplementary Information Figure S5). The chemical shift of C-19 in compound **3** appeared at *δ*_C_ 10.7, in contrast to that of **2** (*δ*_C_ 17.3). This observation is attributed to the diagnostic *γ*-gauche effect between the side chain extending from C-2 and H_3_-19. This distinct chemical environment confirms the change in configuration at C-1. Consequently, based on the NOESY correlations and the supporting NMR chemical shift analysis, compound **3** was identified as the C-1 epimer of Luvunga A [19], named 1-*epi*-Luvunga A (Luvunga B).

Compound **4**

Compound **4** was obtained as an amorphous powder. Its molecular formula was established as C_27_H_34_O_9_ based on the HR-ESI-MS ion peak at *m/z* 503.2254 [M + H]^+^ (calcd. for C_27_H_35_O_9_, 503.2276). The NMR data of **4** were very similar to those of **3** (Table 2), suggesting that they belong to the same class of gedunin-type limonoids. Detailed 2D NMR spectral analysis (Figure 5) revealed that **4** and **3** exhibited similar correlation signals, such as the spin systems observed in the ^1^H-^1^H COSY spectrum and the characteristic HMBC correlations defining the rings A, B, C, and D, as well as the furan moiety at C-17.

However, a major structural difference was identified at C-4. The methoxycarbonyl group found in **3** is replaced by a hydroxymethyl group in **4**, as evidenced by the HMBC correlations from the oxygenated methylene protons H_2_-29 (*δ*_H_ 3.49) to C-4, C-5, and C-28, along with the characteristic carbon signal at *δ*_C_ 66.2 (C-29).

Notably, while **4** shares the same planar skeleton as **3**, it exhibits the opposite stereochemistry at C-1, corresponding to that of **2**. This assignment is strongly corroborated by the ^13^C NMR data regarding the angular methyl C-19 (Table 2). In compound **3**, C-19 appeared at a significantly shielded field (*δ*_C_ 10.7) due to the *γ*-gauche effect caused by the specific orientation of H-1/H-2. In contrast, the C-19 resonance of **4** appeared at *δ*_C_ 20.1, which is closely comparable to that of **2** (*δ*_C_ 17.3). The absence of the upfield shift in **4** indicates that the *γ*-gauche effect is not present, implying that **4** possesses the same C-1 stereochemistry as **2**. The above deductions were further confirmed by a single-crystal X-ray diffraction study of **4** using Cu K*α* radiation (CCDC number: 2424723, Figure 7).

**Figure 8:**
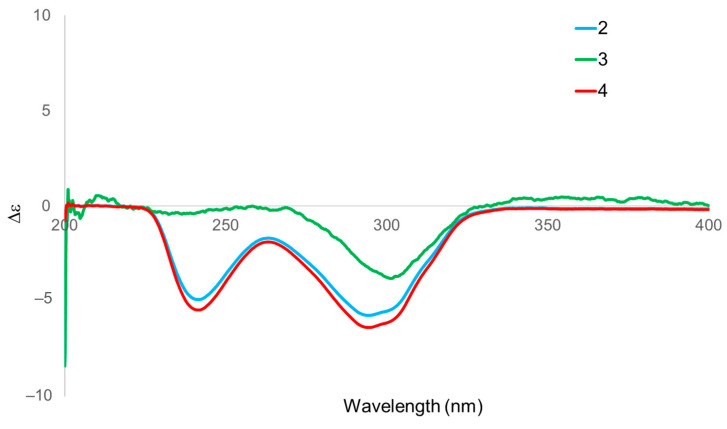
Experimental CD spectra of compounds **2** (blue), **3** (green), and **4** (red).

This result was further corroborated by Circular Dichroism analysis. The CD spectrum (Figure 8) of **4** displayed Cotton effects that were virtually superimposable with those of **2** but differed from those of **3**. Consequently, the absolute configurations of both **2** and **4** were unambiguously assigned as 1*R*, 4*S*, 5*R*, 8*R*, 9*R*, 10*R*, 13*S*, 14*R*, 15*S*, 17*S*, whereas compound **3** was assigned as 1*S*. Thus, the structure of compound **4** was established and named Luvunga C.

Compound **8**

Compound **8** was obtained as a white amorphous powder. It exhibited an optical rotation of ${\left[\alpha \right]}_{D}^{25}$ +34.5 (*c* = 0.3, MeOH). Its molecular formula was determined to be C_26_H_28_O_8_ by HR-ESI-MS, and it exhibited a pseudomolecular ion peak at *m/z* 469.1832 [M + H]^+^ (calcd. 469.1857), indicating 13 degrees of unsaturation. The IR spectrum showed absorption bands for hydroxyl (3600–3400 cm^−1^), carbonyl (1744 cm^−1^), and olefinic (1445 cm^−1^) functionalities. The ^1^H and ^13^C NMR data (Table 2) revealed characteristic signals for a β-substituted furan ring [*δ*_H_ 7.51, 7.49, 6.41; *δ*_C_ 144.5, 142.9, 121.9, 111.0] and two ketone carbonyls (*δ*_C_ 209.4, 208.9), suggesting that **8** is a limonoid derivative.

The planar structure of **8** was determined by extensive analysis of 2D NMR experiments. The spin system of H-9 (*δ*_H_ 3.81)/H-11 (*δ*_H_ 1.98)/H-12 (*δ*_H_ 1.90, 1.53) observed in the COSY spectrum, combined with the HMBC correlations from the angular methyl H_3_-18 (*δ*_H_ 1.21) to C-12, C-13, C-14, and C-17, and from H_3_-30 (*δ*_H_ 1.47) to C-7, C-8, C-9, and C-14, established the fused B/C ring system. (Table 2, Figure 5). Regarding the A/B ring moiety, key HMBC correlations from the olefinic protons permitted the construction of the cyclopentenone ring fused to the six-membered Ring B. The correlations from H-1 (*δ*_H_ 7.93) and H-2 (*δ*_H_ 6.13) to the ketone carbonyl C-3 (*δ*_C_ 209.4) and the quaternary carbon C-10 (*δ*_C_ 62.6) were observed. Most importantly, a key HMBC correlation from H-2 to the bridgehead quaternary carbon C-5 (*δ*_C_ 68.6) established the direct connection between C-3 and C-5. This evidence, combined with the correlation from H-6 (*δ*_H_ 4.32) to C-5, C-7, and C-10, confirmed the formation of the contracted Ring A and its fusion to Ring B. The presence of the propellane skeleton was confirmed by the connectivity of the gem-dimethyl group and the ether bridge. The HMBC correlations from H_3_-28 and H_3_-29 to C-3, C-4, and C-5 located the gem-dimethyl group at C-4. Subsequently, the correlation from the oxygenated methylene protons H_2_-19 (*δ*_H_ 4.18, 3.57) to C-4 (*δ*_C_ 83.0) confirmed the C-4/C-19 ether linkage. These NMR correlations suggest that compound **8** possesses a propellane-like framework. Finally, the structure of the D-ring was elucidated via the characteristic chemical shifts of C-14 (*δ*_C_ 71.5) and C-15 (*δ*_C_ 57.8), which indicated a 14,15-epoxide, and the ester carbonyl C-16 (*δ*_C_ 169.6), consistent with a δ-lactone ring for agreement with the molecular formula and the degrees of unsaturation indicated by the HR-ESI-MS data. Thus, the planar structure of **8** was established. Thus, the planar structure of compound **8** was established. Based on these structural features, compound **8** was identified as a propellane-type limonoid, characterized by the formation of a new C-3/C-5 bond that generates a propellane-like cage framework composed of two five-membered rings fused to the B-ring system. To date, this rare limonoid skeleton has been reported only in a limited number of metabolites. In the present study, 7-hydroxycycloatalantin (**9**) and cycloepiatalantin (**10**) also belong to this rare structural class, together with a few previously reported examples from *Atalantia* species [20,21].

**Figure 9:**
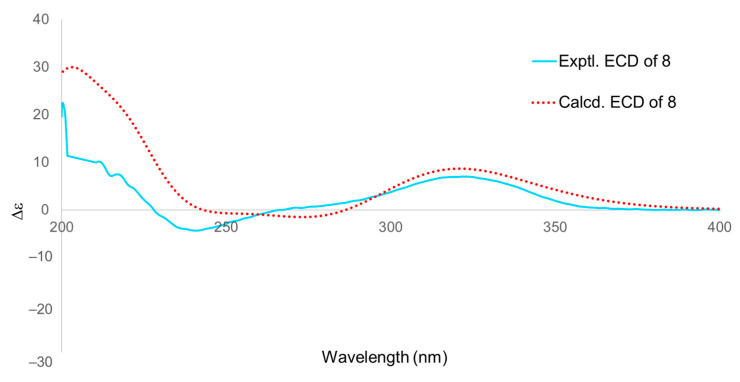
Comparison of the experimental ECD and calculation ECD spectra of compound **8**.

The absolute configuration of **8** was determined by comparing the experimental ECD spectrum (Figure 9) with the calculated data using time-dependent density functional theory. The experimental Cotton effects were in good agreement with the calculated curve for the (5*R*, 6*R*, 8*R*, 9*R*, 10*S*, 13*S*, 14*R*, 15*S*, 17*S*) enantiomer. Consequently, the structure of **8** was unambiguously established and named Luvunga D.

The structures of the remaining known compounds were identified by comparison of their mass spectrometric and NMR spectroscopic data with those reported in the literature. Accordingly, compounds **1**, **2**, **5**–**7**, **9**, and **10** were identified as limonin [22], luvunga A [19], veprisone [23], isoobacunoic acid [24], obacunone [25], 7-hydroxycycloatalantin [26], cycloepiatalantin [21], respectively.

**Scheme 1:**
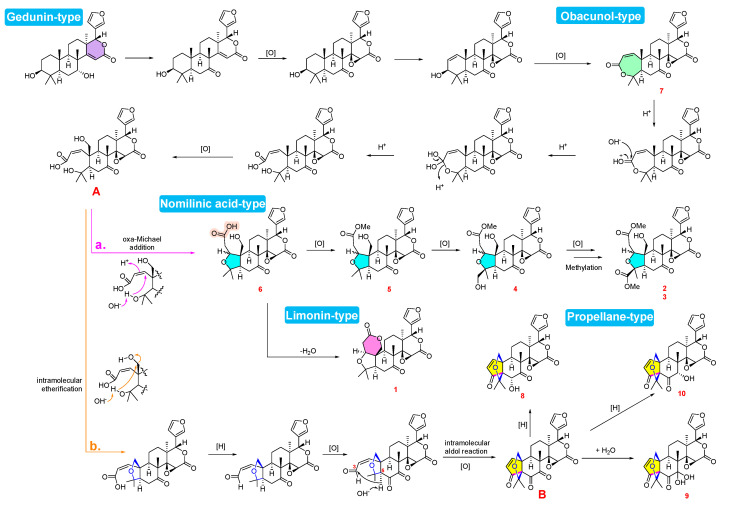
Predicted biosynthesis of limonoids isolated from *L. scandens.* **A, B**: key intermediates; **a, b**: divergent biosynthetic pathways.

Based on the isolated limonoids **1**–**10** and the established biogenetic relationships of *Citrus* limonoids, a plausible biosynthetic pathway for *L. scandens* is proposed and illustrated in Scheme 1. These metabolites are biogenetically derived from the mevalonate pathway, which leads to the fundamental tetranortriterpenoid skeleton. Specifically, the pathway is suggested to originate from a gedunin-type precursor, which undergoes oxidative modifications to furnish obacunone (**7**), an obacunone-type limonoid. Hydrolysis of the A-ring lactone in **7**, followed by oxidation, affords the key acyclic intermediate A. This intermediate serves as a metabolic branch point, diverging into two distinct skeletal pathways. In the first pathway (path a), intermediate A undergoes an oxa-Michael addition to form isoobacunoic acid (**6**), representing an A-ring opened limonoid belonging to the nomilinic acid-type framework. Dehydration of **6** reforms the A-ring lactone to yield limonin (**1**), whereas methylation of **6** produces veprisone (**5**). Subsequently, **5** is oxidized to yield Luvunga C (**4**). Finally, **4** undergoes further oxidation and methylation to generate the C-1 epimeric pair, Luvunga A (**2**) and Luvunga B (**3**).

Alternatively (path b), intermediate A undergoes intramolecular etherification followed by a reduction/oxidation sequence to establish a C-6 ketone. This functionality facilitates an intramolecular aldol reaction between C-3 and C-5, furnishing the propellane-type intermediate B. Subsequent diversification of intermediate B proceeds via hydrolysis to yield **9**, whereas regioselective reduction of the C-6 or C-8 ketones affords **8** and **10**, respectively. In summary, the biosynthetic map of *L. scandens* illustrates the metabolic bifurcation of intermediate A, demonstrating the capacity of this species to generate diverse skeletal architectures through specific cyclization and oxidation pathways [27,28,29,30,31,32,33].

### 3.3. Compound ***8*** Prevents Ferroptosis Through Non-Classical Mechanisms

**Figure 10:**
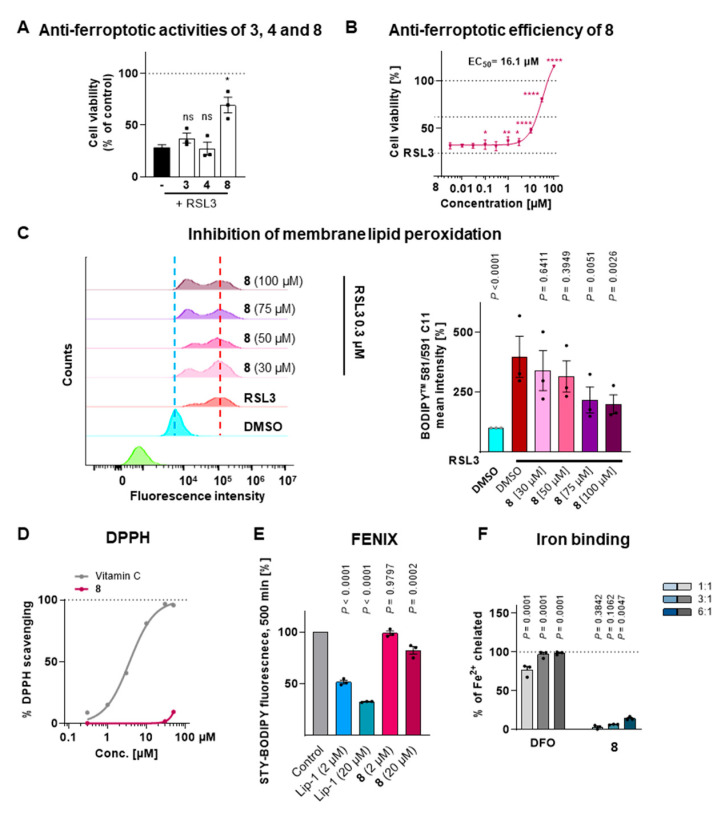
Compound 8 mitigates ferroptosis via a non-classical mechanism. (**A**) Cell viability of HepaRG cells treated with RSL3 (0.3 µM) in the presence of tested compounds (10 µM) for 48 h. Data are shown as mean values ± SEM of *n* = 3 independent experiments. (**B**) Anti-ferroptotic effect of compound **8** in HepaRG cells treated with RSL3 (0.3 µM) for 48 h. Cell viability was determined by MTT assay. (**C**) Lipid peroxidation in HepaRG cells was evaluated by BODIPY 581/591 C11 staining after treatment with compound **8** for 2 h, followed by RSL3 (0.5 µM) treatment for 2 h. Means and individual values ± SEM of *n* = 3 independent experiments. (**D**) Radical scavenging activity of compound **8** was evaluated using the DPPH assay, *n* = 4 independent experiments. (**E**) Inhibition of DTUN (2 µM)-induced lipid peroxidation in liposomes treated with compound **8** (2 and 20 µM), as determined by the FENIX assay. Lip-1 (2 and 20 µM) was used as positive control. Means and individual values ± SEM of *n* = 3 independent experiments. *p* values *vs*. vehicle control, repeated measures one-way ANOVA with Dunnett’s *post-hoc* tests (**F**) Iron-binding capacity was measured using ferene at iron-to-compound ratios of 1:1, 1:2, 1:6, *n* = 3 independent experiments. DFO was used as positive control. *p* values vs. RSL3 (**A**,**B**) or vehicle control (**C**,**E**,**F**), two-tailed paired Student’s *t*-test (**A**) or repeated measures one-way ANOVA with Dunnett’s post-hoc test. ns *p* > 0.05, * *p* < 0.05, ** *p* < 0.01, **** *p* < 0.0001.

Ferroptosis is a regulated form of cell death that has been increasingly recognized as a contributing factor in a variety of pathological conditions, including neurodegenerative diseases such as Alzheimer’s and Parkinson’s, multiple sclerosis, hepatic degeneration, and certain malignancies [1,34]. To assess the anti-ferroptotic effects of the newly isolated limonoids (**3**, **4**, and **8**), these compounds were evaluated for their protective effects against RSL3-induced ferroptosis using human HepaRG liver cells. While compounds **3** and **4** showed no significant effect at a concentration of 10 µM, compound **8** exhibited a strong protection against ferroptotic cell death (Figure 10A). Subsequent dose–response experiments further revealed that compound **8** inhibited ferroptosis with an EC_50_ value of 16.1 μM (Figure 10B). Robust ferroptosis inhibition was already observed at 10 μM, and at 100 μM, compound **8** completely counteracted the cytotoxicity induced by RSL3. Established ferroptosis inhibitors such as liproxstatin-1 (Lip-1) or ferrostatin-1 (Fer-1), which act via direct radical-scavenging mechanisms, typically exhibit EC_50_ values in the low nanomolar range (~20–40 nM in cell-based models) [35,36]. It should be noted that compound **8** was evaluated only in the context of RSL3-induced ferroptosis in the present study. While these results clearly demonstrate the anti-ferroptotic potential of compound **8**, testing its activity against additional ferroptosis inducers such as erastin or FINO2 would provide further insight into whether its protective activity extends beyond the RSL3 model. Lipid peroxidation is a defining feature of ferroptosis. We therefore investigated whether compound **8** could prevent lipid peroxidation in HepaRG cells undergoing RSL3-induced ferroptosis. Compound **8** was found to significantly and dose-dependently inhibit lipid peroxidation in response to RSL3 stimulation (Figure 10C).

Two major categories of ferroptosis inhibitors are (i) radical-scavenging compounds and (ii) iron chelators [37,38]. Radical-scavenging compounds such as Lip-1 or ferrostatin-1 (Fer-1) protect cells from ferroptosis by neutralizing lipid peroxides, thereby interrupting the lipid peroxidation chain reaction. In contrast, iron chelators inhibit ferroptosis by reducing the pool of labile iron and thus preventing the formation of toxic hydroxyl radicals via the Fenton reaction. Compound **8** did not exhibit any radical-scavenging activity in cell-free DPPH assay (Figure 10D). Notably, the DPPH assay often underestimates the cytoprotective potency of lipophilic antioxidants, such as the well-established ferroptosis inhibitors Lip-1 and Fer-1, likely due to their poor radical-trapping efficiency in hydrophilic environments [14]. To directly assess lipid protection, compound **8** was evaluated in the FENIX assay, which monitors inhibition of lipid peroxidation in artificial phospholipid membranes. Unlike Lip-1, compound **8** showed only very weak inhibition, even at 20 µM (Figure 10E). For comparison, strongly active lipophilic antioxidants inhibit >50% of lipid peroxidation at 2 µM, moderately active compounds achieve similar inhibition at 20 µM, whereas weak antioxidants only reach >50% inhibition at very high concentrations (e.g., 200 µM) [39] Thus, compound **8** can be classified as a very weak lipophilic antioxidant, and its anti-ferroptotic activity is unlikely to be explained solely by direct antioxidant effects. Furthermore, in contrast to the well-established iron-chelating ferroptosis inhibitor DFO, compound **8** exhibited only minimal iron-binding capacity (Figure 10F). Although pH-dependent or context-specific interactions cannot be entirely excluded, the structure of compound **8** lacks the structural features required to form stable iron complexes. This contrasts with strong chelators such as DFO, which generate stable multidentate, redox-inactive iron complexes. It is therefore unlikely that the anti-ferroptotic effect of compound **8** is mediated by direct iron binding. Together, these results strongly suggest that compound **8** exerts its anti-ferroptotic activity via non-classical mechanisms, beyond simple radical scavenging or iron chelation.

## 4. Conclusions

In this study, we successfully applied an NMR-guided fractionation strategy to explore the chemical diversity of *Luvunga scandens*, leading to the isolation of ten limonoids, including three new compounds: Luvunga B (**3**), C (**4**), and D (**8**). Based on the structural diversity of the isolates, we proposed a plausible biosynthetic pathway originating from obacunone (**7**), highlighting the divergence into the isoobacunoic acid lineage and the unique propellane-type lineage represented by compound **8**. Luvunga D (**8**) emerged as a potent inhibitor of RSL3-induced ferroptosis in HepaRG liver cells. Whether luvunga D (**8**) is comparably active in other cell lines remains elusive. Notably, unlike classical ferroptosis inhibitors, compound **8** does not exhibit direct radical-scavenging activity or iron-binding capacity in cell-free systems. Nevertheless, it significantly reduced lipid peroxidation in a cellular context, a hallmark of ferroptotic cell death. Together, these findings indicate that the protective effect of compound **8** is unlikely to result from classical radical-trapping or iron-chelating mechanisms and instead point to an indirect cellular mechanism. The precise pathways involved remain to be elucidated. Consequently compound **8** represents a promising scaffold for the development of novel therapeutic agents targeting ferroptosis-related oxidative stress diseases.

## Figures and Tables

**Figure 7:**
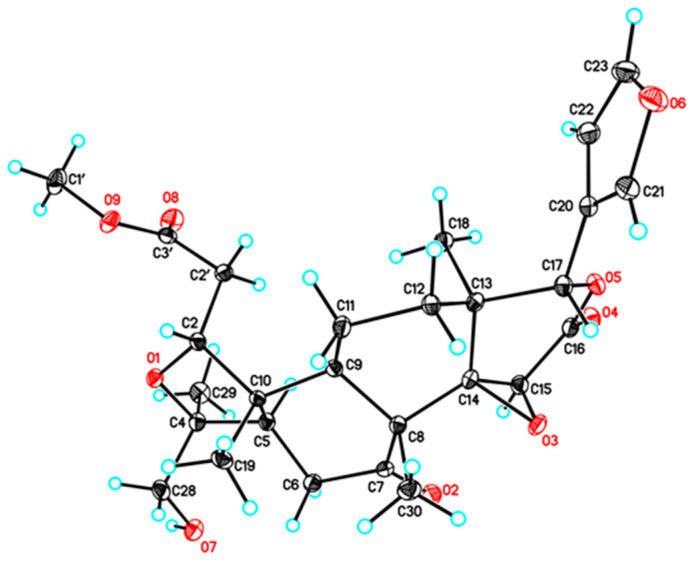
ORTEP views of the X-ray structure of compound **4**.

**Table 1:** ^1^H (600 MHz) and ^13^C (150 MHz) NMR Spectroscopic Data of **2** and **3** in CDCl_3._

No.	2		3	
	*δ*_C_	*δ*_H_ (*J* in Hz)	*δ*_C_	*δ*_H_ (*J* in Hz)
1	84.7, CH	4.32 dd (3.0; 10.2)	84.4, CH	4.04 dd (2.4; 8.4)
2	37.3, CH_2_	2.32 m 2.22 m	36.7, CH_2_	2.64 m 2.55 m
3	170.5, C	-	171.5, C	-
4	82.0, C	-	81.0, C	-
5	56.4, CH	2.08 dd (4.2; 15.6)	60.8, CH	2.05 dd (3.6; 15.6)
6	37.5, CH_2_	3.09 t (15.0) 2.61 dd (3.6; 14.4)	37.4, CH_2_	3.01 m 2.62 m
7	208.1, C	-	207.8, C	-
8	51.4, C	-	50.9, C	-
9	42.6, CH	2.15 dd (2.4; 12.0)	46.2, CH	2.32 dd (3.6; 12.0)
10	46.0, C	-	45.0, C	-
11	18.4, CH_2_	1.83 m 1.50 m	18.2, CH_2_	1.76 m 1.57 m
12	30.8, CH_2_	1.73 m 1.35 m	29.3, CH_2_	1.69 m 1.24 ovL
13	37.9, C	-	38.6, C	-
14	65.8, C	-	67.0, C	-
15	53.9, CH	3.99 s	54.7, CH	4.26 s
16	166.9, C	-	167.2, C	-
17	77.9, CH	5.44 s	77.8, CH	5.49 s
18	20.9, CH_3_	1.13 s	20.3, CH_3_	1.17 s
19	17.3, CH_3_	1.18 s	10.7, CH_3_	0.98 s
20	120.1, C	-	120.2, C	-
21	141.1, CH	7.37 d (0.8)	141.1, CH	7.39 d (0.8)
22	109.7, CH	6.31 dd (1.8; 0.8)	109.8, CH	6.31 dd (1.9; 0.9)
23	143.1, CH	7.36 d (1.8)	143.1, CH	7.38 t (1.8)
28	27.0, CH_3_	1.46 s	25.7, CH_3_	1.45 s
29	173.5, C	-	173.6, C	-
30	17.3, CH_3_	1.13 s	18.3, CH_3_	1.19 s
3-OMe	52.1, OCH_3_	3.65 s	52.1, OCH_3_	3.69 s
29-OMe	52.3, OCH_3_	3.71 s	52.2, OCH_3_	3.71 s

**Table 2:** ^1^H (600 MHz) and ^13^C (150 MHz) NMR Spectroscopic Data pertaining to **2**–**4** and **8** in CD_3_OD.

No.	2		3		4		8	
	*δ*_C_	*δ*_H_ (*J* in Hz)	*δ*_C_	*δ*_H_ (*J* in Hz)	*δ*_C_	*δ*_H_ (*J* in Hz)	*δ*_C_	*δ*_H_ (*J* in Hz)
1	86.6, CH	4. 27 dd (3.0; 10.8)	85.8, CH	4.09 dd (2.4; 9.0)	85.7, CH	4.14 dd (3.6; 11.4)	85.7, CH	4.14 dd (3.6; 11.4)
2	38.5, CH_2_	3.08 t (15.0) 2.51 m	37.7, CH_2_	2.68 dd (2.4; 16.2) 2.56 dd (9.6; 16.8)	37.9, CH_2_	2.55 dd (11.4; 14.4) 2.39 dd (3.6; 13.8)	37.9, CH_2_	2.55 dd (11.4; 14.4) 2.39 dd (3.6; 13.8)
3	173.0, C	-	173.5, C	-	173.3, C	-	173.3, C	-
4	83.4, C	-	82.4, C	-	83.5, C	-	83.5, C	-
5	56.3, CH	2.39 m	60.8, CH	2.32 dd (3.6; 15.6)	54.4, CH	2.30 m	54.4, CH	2.30 m
6	38.1, CH_2_	2.51 m 2.39 m	38.4, CH_2_	3.01 t (15.6) 2.51 dd (3.6; 15.0)	37.2, CH_2_	2.97 m	37.2, CH_2_	2.39 m 2.97 m
7	209.9, C	-	210.0, C	-	210.1, C	-	210.1, C	-
8	52.4, C	-	52.0, C	-	52.5, C	-	52.5, C	-
9	43.3, CH	2.25 dd (3.0; 12.6)	46.8, CH	2.43 dd (3.6; 12.0)	44.3, CH	2.31 m	44.3, CH	2.31 m
10	47.3, C	-	46.3, C	-	48.3, C	-	48.3, C	-
11	19.0, CH_2_	1.89 m 1.55 m	19.0, CH_2_	1.83 m 1.62 m	18.9, CH_2_	1.90 m 1.54 m	18.9, CH_2_	1.90 m 1.54 m
12	31.2, CH_2_	1.80 m 1.39 m	29.9, CH_2_	1.78 m 1.39 m	31.1, CH_2_	1.80 m 1.38 m	31.1, CH_2_	1.80 m 1.38 m
13	39.4, C	-	40.0, C	-	39.6, C	-	39.6, C	-
14	67.9, C	-	69.0, C	-	68.2, C	-	68.2, C	-
15	55.3, CH	4.12 s	55.4, CH	4.30 s	55.4, CH	4.14 s	55.4, CH	4.14 s
16	169.7, C	-	169.9, C	-	169.8, C	-	169.8, C	-
17	79.6, CH	5.53 s	79.6, CH	5.56 s	79.6, CH	5.54 s	79.6, CH	5.54 s
18	21.0, CH_3_	1.17 s	20.3, CH_3_	1.19 s	21.1, CH_3_	1.18 s	21.1, CH_3_	1.18 s
19	17.6, CH_3_	1.17 s	10.8, CH_3_	0.98 s	20.1, CH_3_	1.38 s	20.1, CH_3_	1.38 s
20	121.8, C	-	121.9, C	-	121.9, C	-	121.9, C	-
21	142.7, CH	7.51 d (0.8)	142.7, CH	7.50 d (0.8)	142.7, CH	7.51 d (0.8)	142.7, CH	7.51 d (0.8)
22	111.0, CH	6.42 dd (1.2; 1.8)	111.0, CH	6.41 dd (1.9; 0.9)	111.0, CH	6.42 dd (1.9; 0.9)	111.0, CH	6.42 dd (1.9; 0.9)
23	144.4, CH	7.47 t (1.7)	144.4, CH	7.48 t (1.8)	144.4, CH	7.47 t (1.8)	144.4, CH	7.47 t (1.8)
28	27.1, CH_3_	1.43 s	25.8, CH_3_	1.40 s	26.5, CH_3_	1.30 s	26.5, CH_3_	1.30 s
29	175.6, C	-	175.6, C	-	66.2, CH_2_	3.49 dd (11.4; 19.8)	66.2, CH_2_	3.49 dd (11.4; 19.8)
30	18.2, CH_3_	1.19 s	19.0, CH_3_	1.23 s	18.3, CH_3_	1.21 s	18.3, CH_3_	1.21 s
3-OMe	52.3, CH_3_	3.66 s	52.3, CH_3_	3.68 s	52.2, CH_3_	3.65 s	52.2, CH_3_	3.65 s
29-OMe	52.5, CH_3_	3.70 s	52.5, CH_3_	3.70 s	-	-	-	-

## Data Availability

The original contributions presented in this study are included in the article/Supplementary Material. Further inquiries can be directed to the corresponding authors.

## Supplementary Materials

The following supporting information can be downloaded at https://www.mdpi.com/article/10.3390/antiox15030402/s1. Figure S1: HR-ESI-MS spectrum of compound **3**; Figure S2: UV spectrum of compound **3** in methanol; Figure S3: IR spectrum (film on KBr plates) of compound **3**; Figure S4: ^1^H-NMR (600 MHz) spectrum of compound **3** in CDCl_3_; Figure S5: ^13^C-NMR (150 MHz) spectrum of compound **3** in CDCl_3_; Figure S6: DEPT-NMR (150 MHz) spectrum of compound **3** in CDCl_3_; Figure S7: HSQC spectrum of compound **3** in CDCl_3_; Figure S8: HMBC spectrum of compound **3** in CDCl_3_; Figure S9: ^1^H-^1^H-COSY spectrum of compound **3** in CDCl_3_; Figure S10: ^1^H-^1^H-NOESY spectrum of compound **3** in CDCl_3_; Figure S11: ^1^H-NMR (600 MHz) spectrum of compound **2** in CDCl_3_; Figure S12: ^13^C-NMR (150 MHz) spectrum of compound **2** in CDCl_3_; Figure S13: DEPT-NMR (150 MHz) spectrum of compound **2** in CDCl_3_; Figure S14: HSQC spectrum of compound **2** in CDCl_3_; Figure S15: HMBC spectrum of compound **2** in CDCl_3_; Figure S16: ^1^H-^1^H-COSY spectrum of compound **2** in CDCl_3_; Figure S17: ^1^H-^1^H-NOESY spectrum of compound **2** in CDCl_3_; Figure S18: HR-ESI-MS spectrum of compound **4**; Figure S19: UV spectrum of compound **4** in methanol; Figure S20: IR spectrum (film on KBr plates) of compound **4**; Figure S21: ^1^H-NMR (600 MHz) spectrum of compound **4** in CD_3_OD; Figure S22: ^13^C-NMR (150 MHz) spectrum of compound **4** in CD_3_OD; Figure S23: DEPT-NMR (150 MHz) spectrum of compound **4** in CD_3_OD; Figure S24: HSQC spectrum of compound **4** in CD_3_OD; Figure S25: HMBC spectrum of compound **4** in CD_3_OD; Figure S26: ^1^H-^1^H-COSY spectrum of compound **4** in CD_3_OD; Figure S27: ^1^H-NMR (600 MHz) spectrum of compound **2** in CD_3_OD; Figure S28: ^13^C-NMR (150 MHz) spectrum of compound **2** in CD_3_OD; Figure S29: HSQC spectrum of compound **2** in CD_3_OD; Figure S30: HMBC spectrum of compound **2** in CD_3_OD; Figure S31: ^1^H-^1^H-COSY spectrum of compound **2** in CD_3_OD; Figure S32: ^1^H-NMR (600 MHz) spectrum of compound **3** in CD_3_OD; Figure S33: ^13^C-NMR (150 MHz) spectrum of compound **3** in CD_3_OD; Figure S34: HSQC spectrum of compound **3** in CD_3_OD; Figure S35: HMBC spectrum of compound **3** in CD_3_OD; Figure S36: ^1^H-^1^H-COSY spectrum of compound **3** in CD_3_OD; Figure S37: HR-ESI-MS spectrum of compound **8**; Figure S38: UV spectrum of compound **8** in methanol; Figure S39: IR spectrum (film on KBr plates) of compound **8**; Figure S40: ^1^H-NMR (600 MHz) spectrum of compound **8** in CD_3_OD; Figure S41: ^13^C-NMR (150 MHz) spectrum of compound **8** in CD_3_OD; Figure S42: DEPT-NMR (150 MHz) spectrum of compound **8** in CD_3_OD; Figure S43: HSQC spectrum of compound **8** in CD_3_OD; Figure S44: HMBC spectrum of compound **8** in CD_3_OD; Figure S45: ^1^H-^1^H-COSY spectrum of compound **8** in CD_3_OD; Table S1: Crystal data and experimental details for compound **4**; Table S2: Optical Rotation of compound **3**; Table S3: Optical Rotation of compound **4**; Table S4: Optical Rotation of compound **8**; Table S5: ECD calculation of compound **8**.

## Abbreviations

The following abbreviations are used in this manuscript:

BODIPY	Boron-dipyrromethene
CD	Circular Dichroism
COSY	Homonuclear Correlation Spectroscopy
DEPT	Distortionless Enhancement by Polarization Transfer
DMSO	Dimethyl sulfoxide
DPPH	2,2-diphenyl-1-picrylhydrazyl
EC_50_	Half Maximal Effective Concentration
ECD	Electronic Circular Dichroism
FENIX	Fluorescence-enabled inhibited autoxidation
FT-IR	Fourier transform infrared spectroscopy
HEPES	4-(2-hydroxyethyl)-1-piperazineethanesulfonic acid
HMBC	Heteronuclear Multiple Bond Correlation.
HPLC	High-Performance Liquid Chromatography
HR-ESI-MS	High-Resolution Electrospray Ionization Mass Spectrometry
IR	Infrared spectrum
LCMS	Liquid Chromatography-Mass Spectrometry
MTT	3-(4,5-dimethylthiazol-2-yl)-2,5-diphenyltetrazolium bromide
NMR	Nuclear Magnetic Resonance
NOESY	Nuclear Overhauser Effect Spectroscopy
ODS	Octadecylsilane
QTOF	Quadrupole Time-of-Flight
RSL3	RAS-selective lethal 3
UV	Ultraviolet-Visible
