# Supplementary material for: NMR-Guided Discovery of Luvunga D: A Novel Propellane-Type Limonoid from Luvunga scandens That Functions as a Non-Classical Ferroptosis Inhibitor
Source: Antioxidants (Basel). 2026 Mar 23;15(3):402. doi: 10.3390/antiox15030402 (PMC13024304; doi:10.3390/antiox15030402)
Supplement: Supplementary file 1 [file antioxidants-15-00402-s001.zip › antioxidants-4188584-supplementary.pdf]

Supporting information

NMR-Guided Discovery of Luvunga D: A Novel Propellane-Type Limonoid from *Luvunga scandens* That Functions as a Non-Classical Ferroptosis Inhibitor

Bien-Thuy Bui Nguyen [1,2,3], *Hoang-Minh Bui* [4,5], *Chia-Ching Liaw* [2,6,7], *Quoc-Dung Tran Huynh* [8], *Chih-Hua Chao* [9,10], *Duy-Hien Tran* [1,2,11], *I-Wen Lo* [2], *Thanh-Hoa Vo* [3,12], *Andreas Koeberle* [4,13], *Solveigh C. Koeberle* [13], *Mei-Chuan Chen* [1,14,15,*], *Yu-Chi Lin* [2,*]

1 Ph.D. Program in Clinical Drug Development of Herbal Medicine, College of Pharmacy, Taipei Medical University, Taipei 110301, Taiwan; bnbthuy@uhsvnu.edu.vn (B.-T.B.N.); tranduyhien@ump.edu.vn (D.-H.T.)

2 National Research Institute of Chinese Medicine, Taipei 112026, Taiwan; liawcc@nricm.edu.tw (C.-C.L.); iwenlo99@nricm.edu.tw (I.-W.L.)

3 Department of Pharmacognosy, Faculty of Pharmacy, University of Health Sciences, Vietnam National University Ho Chi Minh City, Ho Chi Minh City 700000, Vietnam; vthoa@uhsvnu.edu.vn

4 Michael Popp Institute, Center for Molecular Biosciences Innsbruck (CMBI), University of Innsbruck, 6020 Innsbruck, Austria; minh.bui-hoang@student.uibk.ac.at (H.-M.B.); andreas.koeberle@uni-graz.at (A.K.)

5 Unit of Pharmacognosy, Institute of Pharmacy, Center for Molecular Biosciences Innsbruck (CMBI), University of Innsbruck, 6020 Innsbruck, Austria

6 Department of Pharmacy, School of Pharmaceutical Sciences, National Yang Ming Chiao Tung University, Taipei 112304, Taiwan

7 Graduate Institute of Natural Products, Kaohsiung Medical University, Kaohsiung 807378, Taiwan

8 Institute of Biological Chemistry, Academia Sinica, Taipei 115201, Taiwan; htqdung3012@as.edu.tw

9 School of Pharmacy, China Medical University, Taichung 406040, Taiwan; chchao@mail.cmu.edu.tw

10 Chinese Medicine Research and Development Center, China Medical University Hospital, Taichung 404327, Taiwan

11 Department of Pharmacognosy–Traditional Pharmacy, School of Pharmacy, University of Medicine and Pharmacy at Ho Chi Minh City, Ho Chi Minh City 700000, Vietnam

12 Research Center for Discovery and Development of Healthcare Products, Vietnam National University Ho Chi Minh City, Ho Chi Minh City 700000, Vietnam
13 Institute of Pharmaceutical Sciences, Pharmacognosy and Excellence Field BioHealth, NAWI Graz, University of Graz, 8010 Graz, Austria; solveigh.koeberle@uni-graz.at
14 School of Pharmacy, College of Pharmacy, Taipei Medical University, Taipei 110301, Taiwan
15 Traditional Herbal Medicine Research Center, Taipei Medical University Hospital, Taipei 110301, Taiwan

* Correspondence: mcchen1250@tmu.edu.tw (M.-C.C.); yclin@nricm.edu.tw (Y.-C.L.); Tel.: +886-2-27361661 (ext. 6184) (M.-C.C.); +886-2-28201999 (ext. 7052) (Y.-C.L.)

Figure S 1. HR-ESI-MS spectrum of compound **3** 4
Figure S 2. UV spectrum of compound **3** in methanol 5
Figure S 3. IR spectrum (film on KBr plates) of compound **3** 6
Figure S 4. ^{1}H-NMR (600 MHz) spectrum of compound **3** in $CDCl_3$ 7
Figure S 5. ^{13}C-NMR (150 MHz) spectrum of compound **3** in $CDCl_3$ 8
Figure S 6. DEPT-NMR (150 MHz) spectrum of compound **3** in $CDCl_3$ 9
Figure S 7. HSQC spectrum of compound **3** in $CDCl_3$ 10
Figure S 8. HMBC spectrum of compound **3** in $CDCl_3$ 11
Figure S 9. ^{1}H-^{1}H-COSY spectrum of compound **3** in $CDCl_3$ 12
Figure S 10. ^{1}H-^{1}H-NOESY spectrum of compound **3** in $CDCl_3$ 13
Figure S 11. ^{1}H-NMR (600 MHz) spectrum of compound **2** in $CDCl_3$ 14
Figure S 12. ^{13}C-NMR (150 MHz) spectrum of compound **2** in $CDCl_3$ 15
Figure S 13. DEPT-NMR (150 MHz) spectrum of compound **2** in $CDCl_3$ 16
Figure S 14. HSQC spectrum of compound **2** in $CDCl_3$ 17
Figure S 15. HMBC spectrum of compound **2** in $CDCl_3$ 18
Figure S 16. ^{1}H-^{1}H-COSY spectrum of compound **2** in $CDCl_3$ 19
Figure S 17. ^{1}H-^{1}H-NOESY spectrum of compound **2** in $CDCl_3$ 20
Figure S 18. HR-ESI-MS spectrum of compound **4** 21
Figure S 19. UV spectrum of compound **4** in methanol 22
Figure S 20. IR spectrum (film on KBr plates) of compound **4** 23
Figure S 21. ^{1}H-NMR (600 MHz) spectrum of compound **4** in CD_3OD 24
Figure S 22. ^{13}C-NMR (150 MHz) spectrum of compound **4** in CD_3OD 25
Figure S 23. DEPT-NMR (150 MHz) spectrum of compound **4** in CD_3OD 26

Figure S 24. HSQC spectrum of compound **4** in CD_3OD 27
Figure S 25. HMBC spectrum of compound **4** in CD_3OD 28
Figure S 26. ^1H-^1H-COSY spectrum of compound **4** in CD_3OD 29
Figure S 27. ^1H-NMR (600 MHz) spectrum of compound **2** in CD_3OD 30
Figure S 28. ^{13}C-NMR (150 MHz) spectrum of compound **2** in CD_3OD 31
Figure S 29. HSQC spectrum of compound **2** in CD_3OD 32
Figure S 30. HMBC spectrum of compound **2** in CD_3OD 33
Figure S 31. ^1H-^1H-COSY spectrum of compound **2** in CD_3OD 34
Figure S 32. ^1H-NMR (600 MHz) spectrum of compound **3** in CD_3OD 35
Figure S 33. ^{13}C-NMR (150 MHz) spectrum of compound **3** in CD_3OD 36
Figure S 34. HSQC spectrum of compound **3** in CD_3OD 37
Figure S 35. HMBC spectrum of compound **3** in CD_3OD 38
Figure S 36. ^1H-^1H-COSY spectrum of compound **3** in CD_3OD 39
Figure S 37. HR-ESI-MS spectrum of compound **8** 40
Figure S 38. UV spectrum of compound **8** in methanol 41
Figure S 39. IR spectrum (film on KBr plates) of compound **8** 42
Figure S 40. ^1H-NMR (600 MHz) spectrum of compound **8** in CD_3OD 43
Figure S 41. ^{13}C-NMR (150 MHz) spectrum of compound **8** in CD_3OD 44
Figure S 42. DEPT-NMR (150 MHz) spectrum of compound **8** in CD_3OD 45
Figure S 43. HSQC spectrum of compound **8** in CD_3OD 46
Figure S 44. HMBC spectrum of compound **8** in CD_3OD 47
Figure S 45. ^1H-^1H-COSY spectrum of compound **8** in CD_3OD 48
Table S 1. Crystal data and experimental details for compound **4** 49
Table S 2. Optical Rotation of compound **3** 51
Table S 3. Optical Rotation of compound **4** 52
Table S 4. Optical Rotation of compound **8** 53
Table S 5. ECD calculation of compound **8** 54

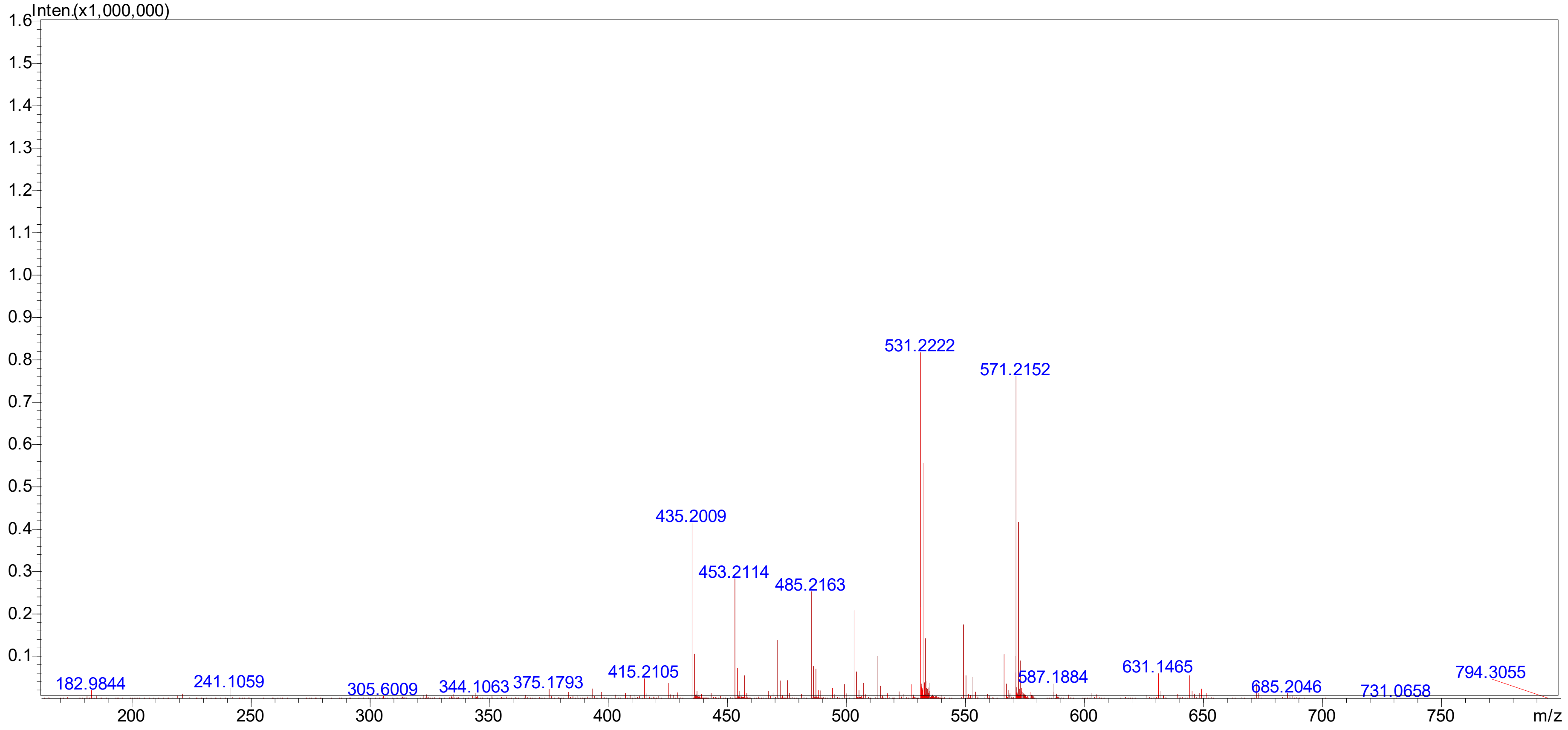

Figure S 1. HR-ESI-MS spectrum of compound **3**

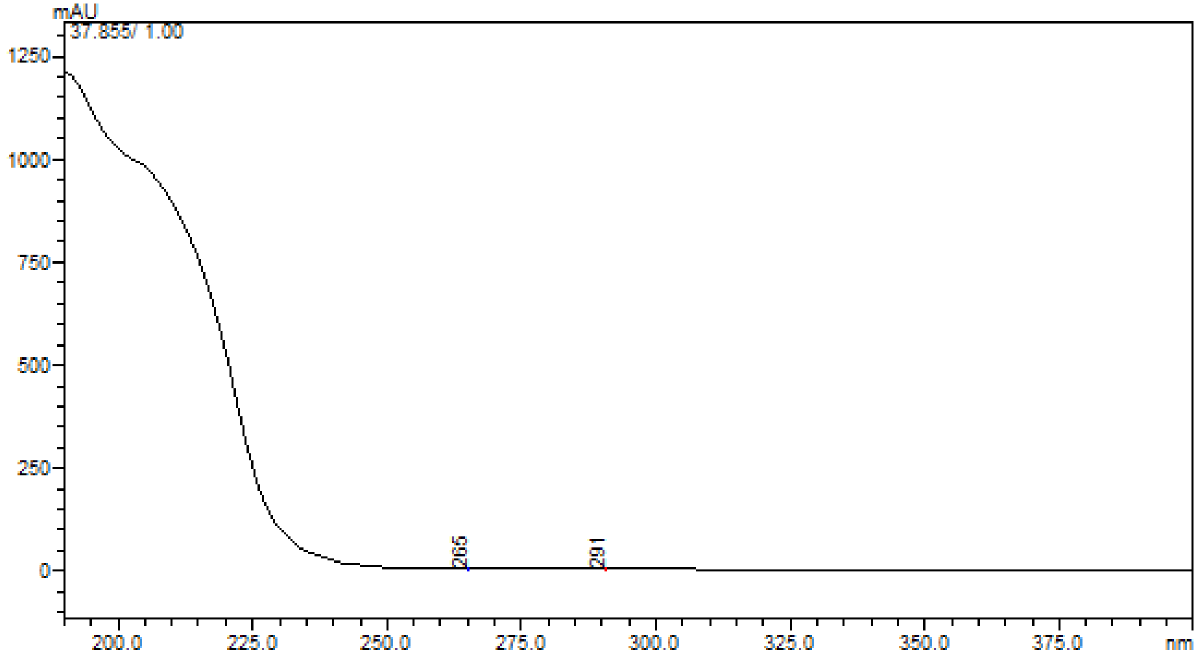

Figure S 2. UV spectrum of compound **3** in methanol

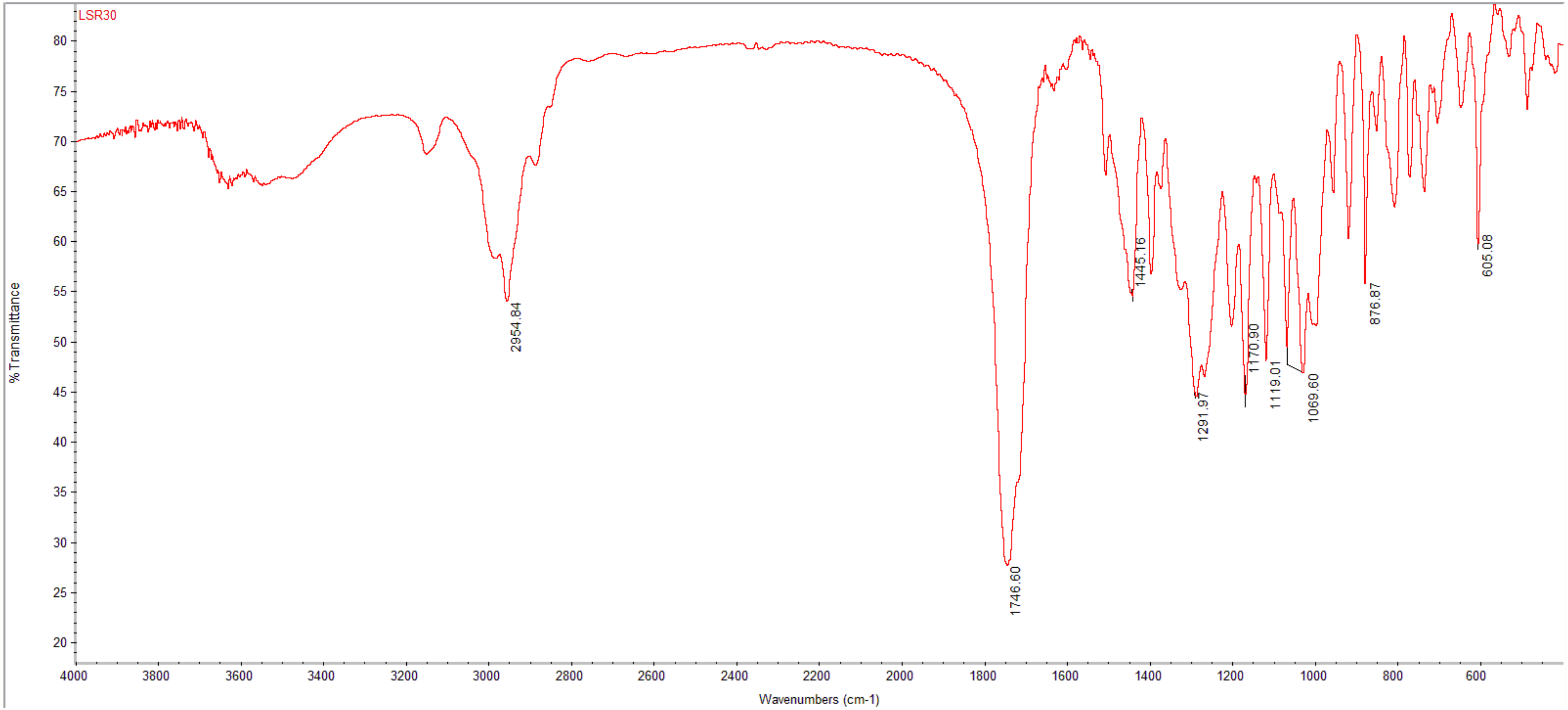

Figure S 3. IR spectrum (film on KBr plates) of compound **3**

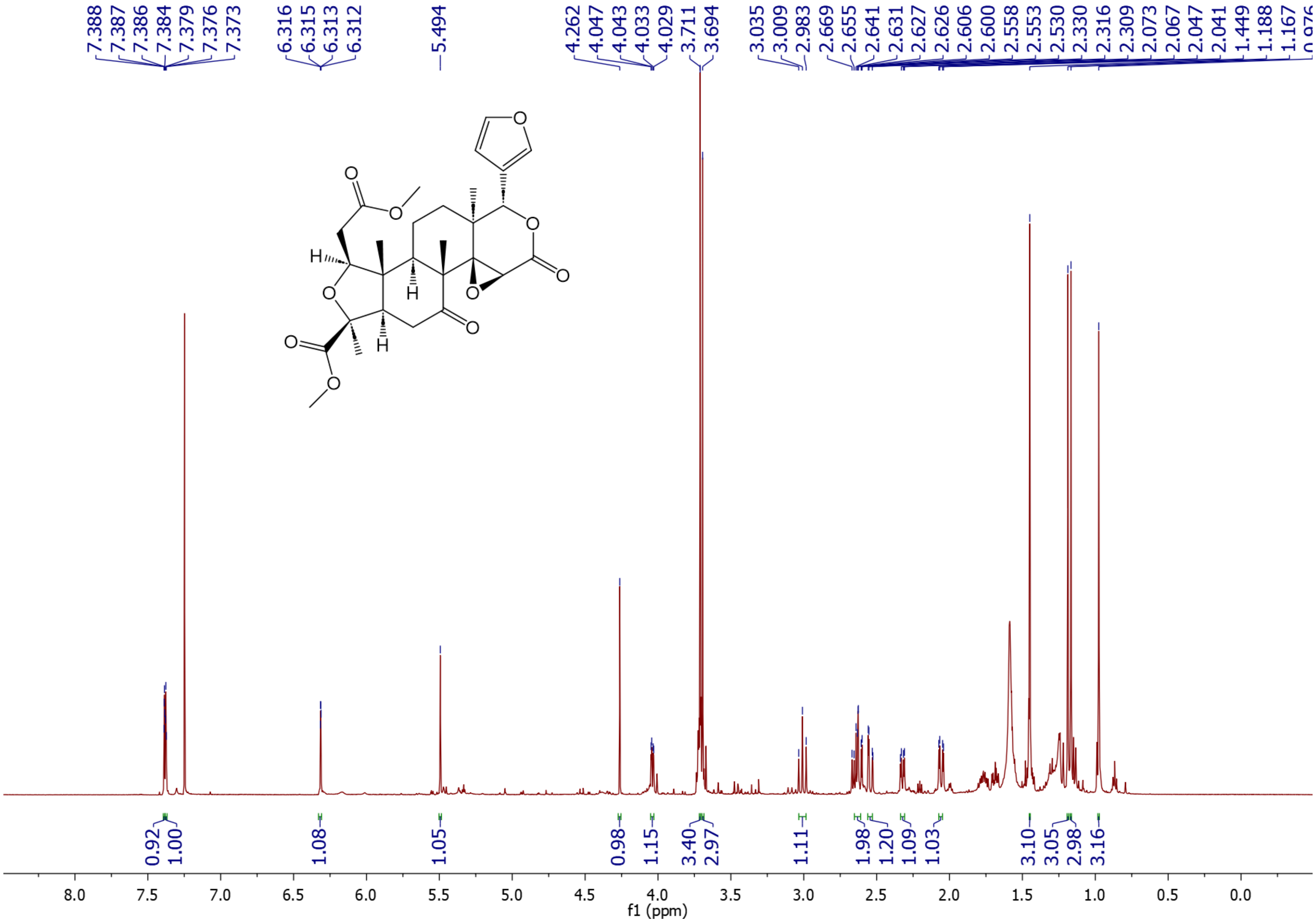

Figure S 4. ^{1}H-NMR (600 MHz) spectrum of compound **3** in $CDCl_3$

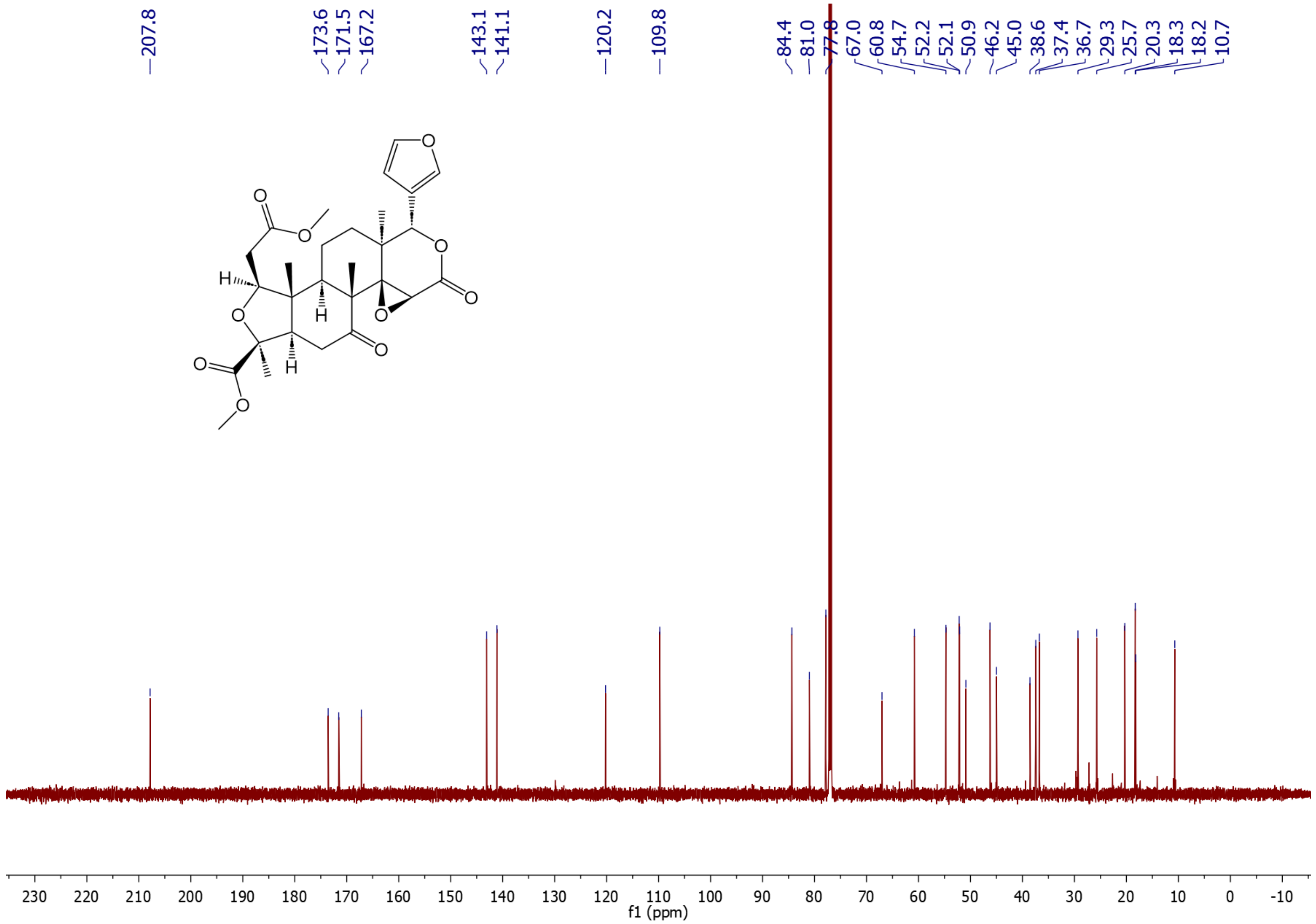

Figure S 5. ^{13}C-NMR (150 MHz) spectrum of compound **3** in $CDCl_3$

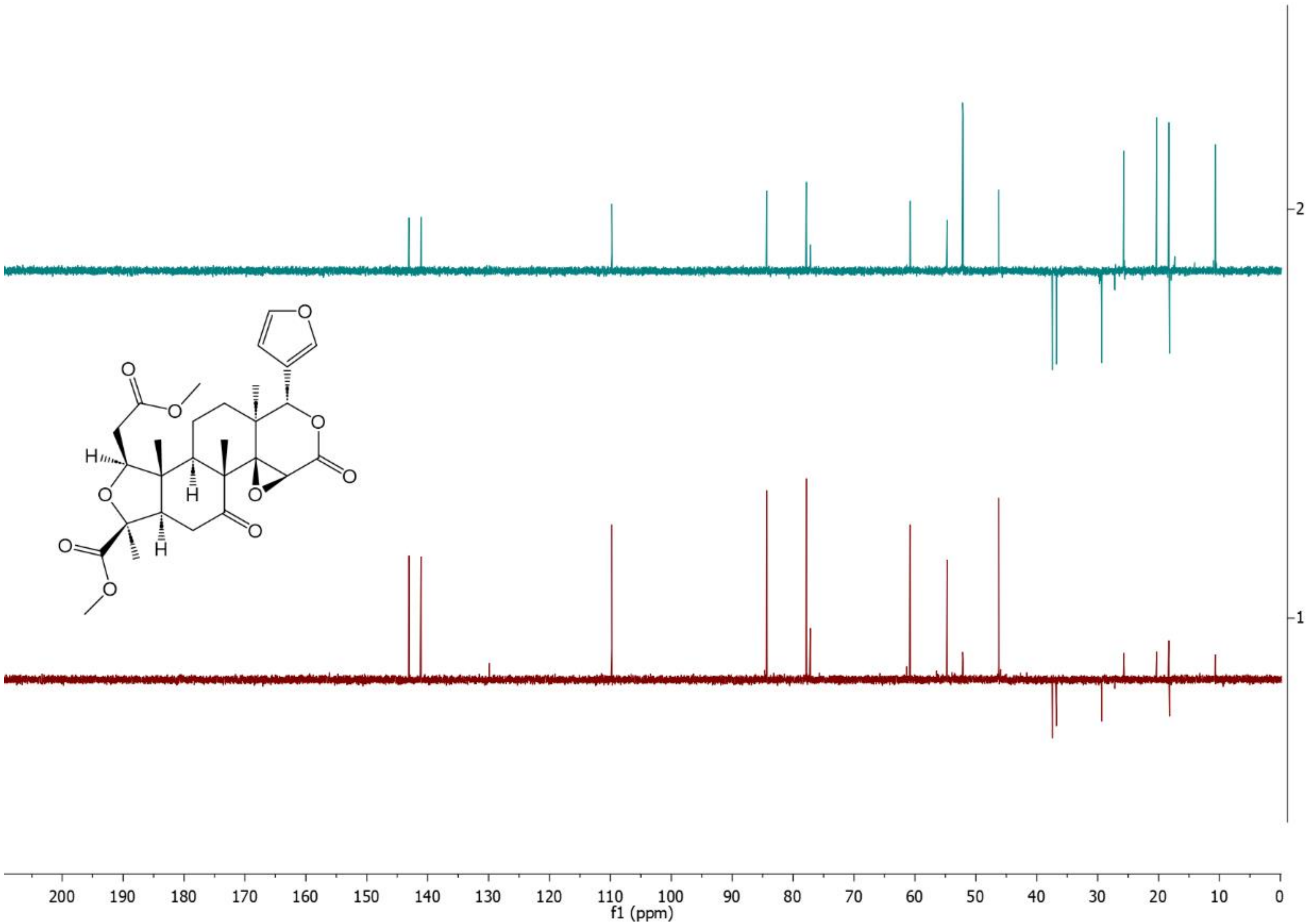

Figure S 6. DEPT-NMR (150 MHz) spectrum of compound **3** in $CDCl_3$

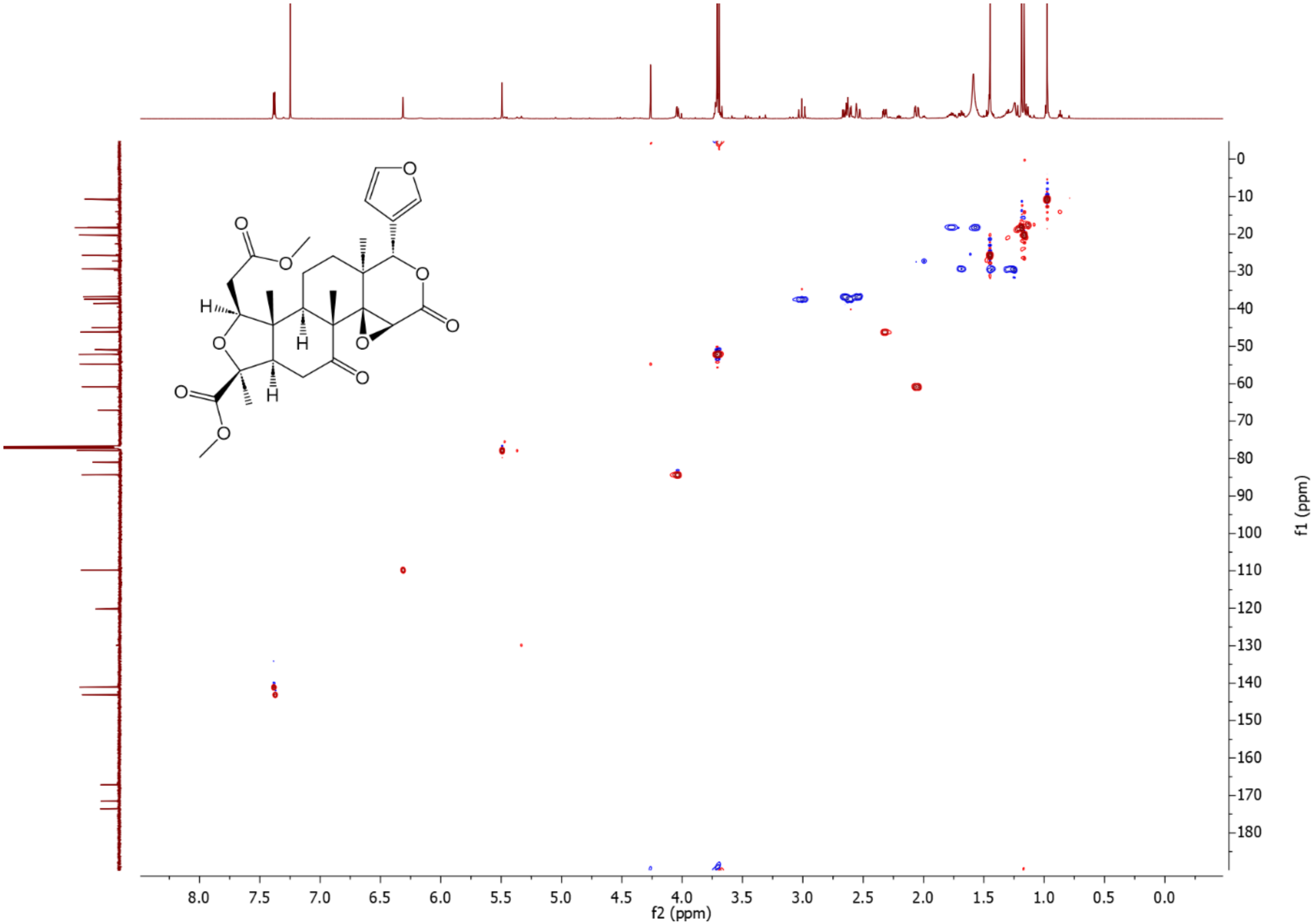

Figure S 7. HSQC spectrum of compound **3** in $CDCl_3$

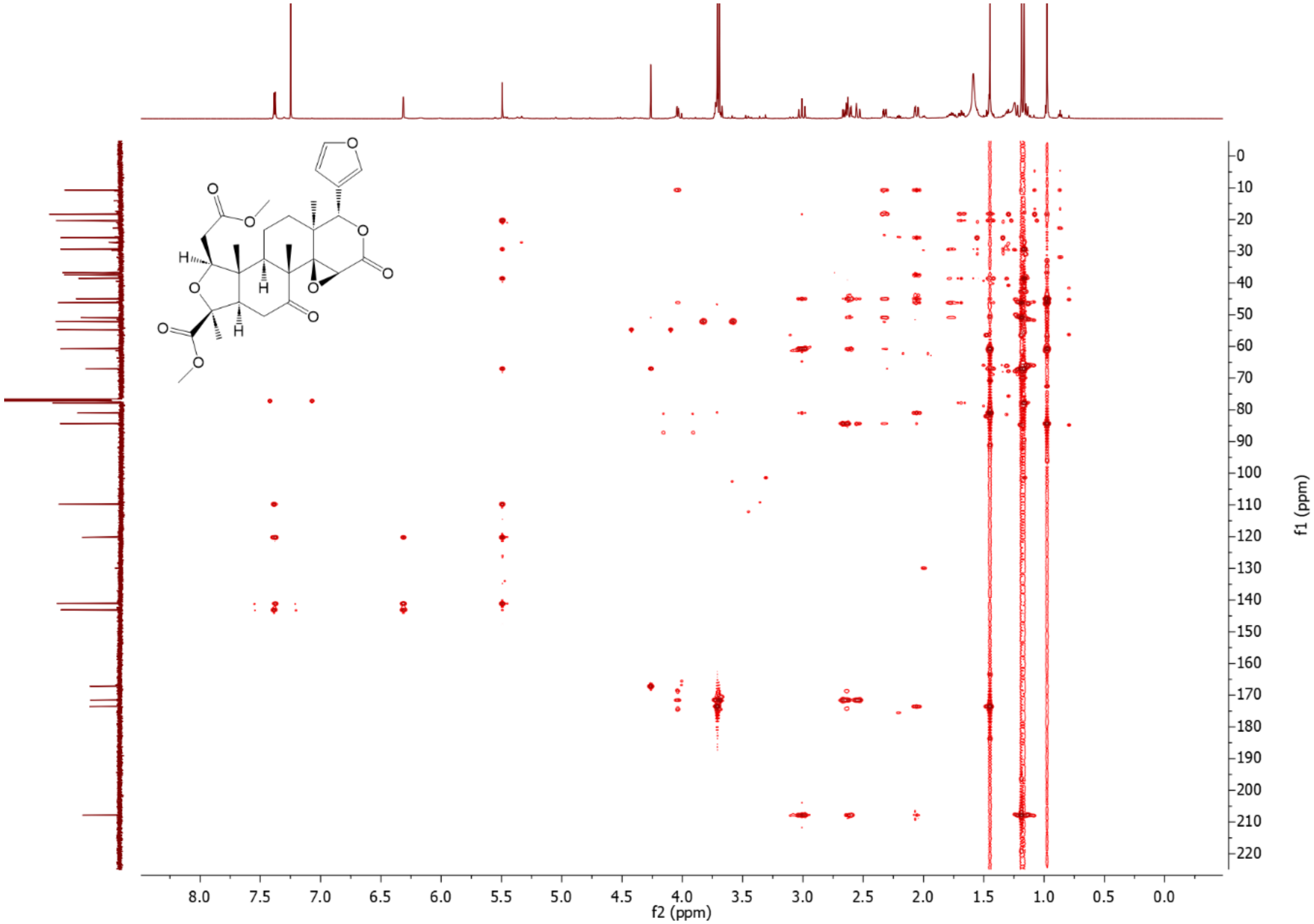

Figure S 8. HMBC spectrum of compound **3** in $CDCl_3$

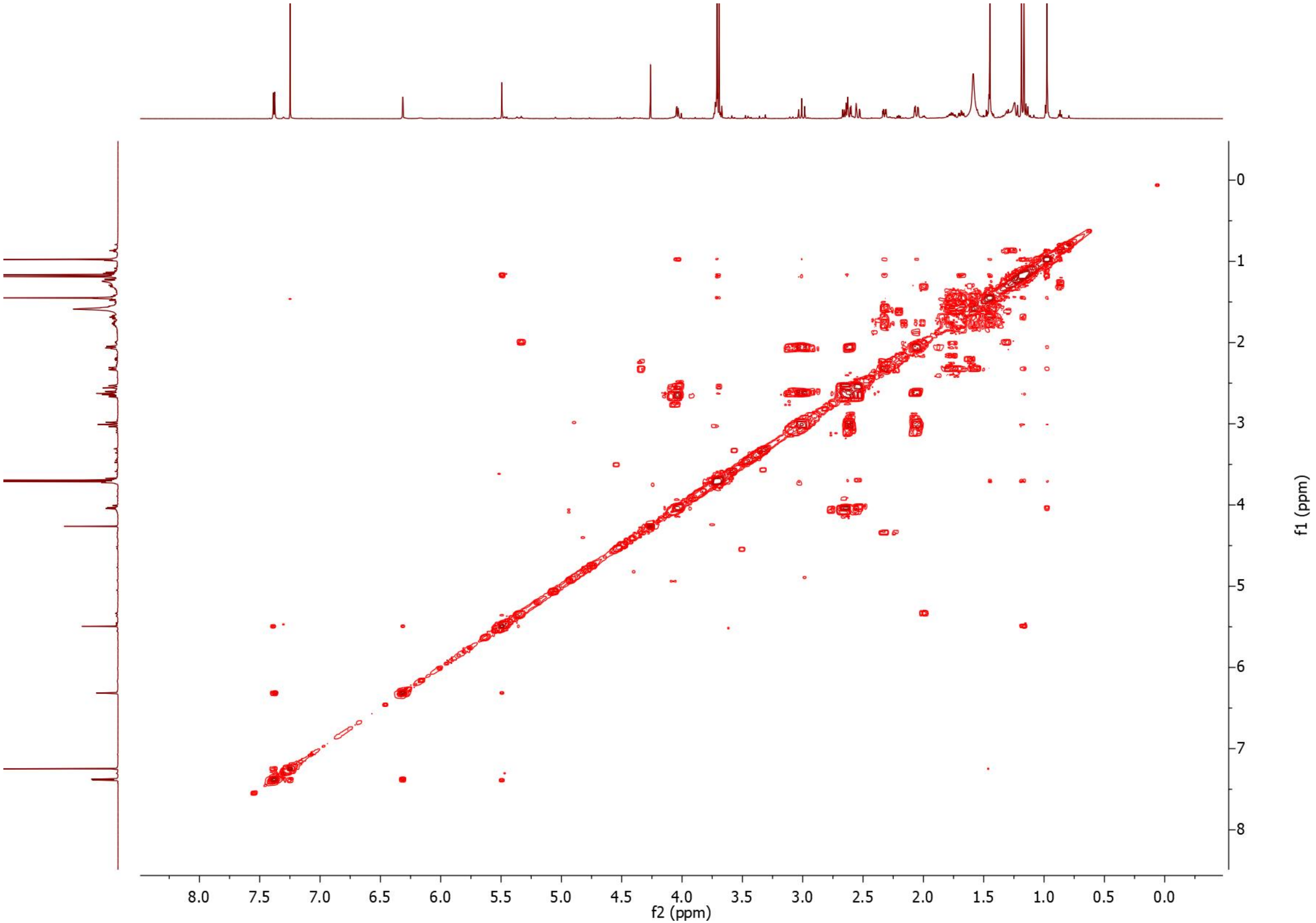

Figure S 9. ^{1}H-^{1}H-COSY spectrum of compound **3** in $CDCl_3$

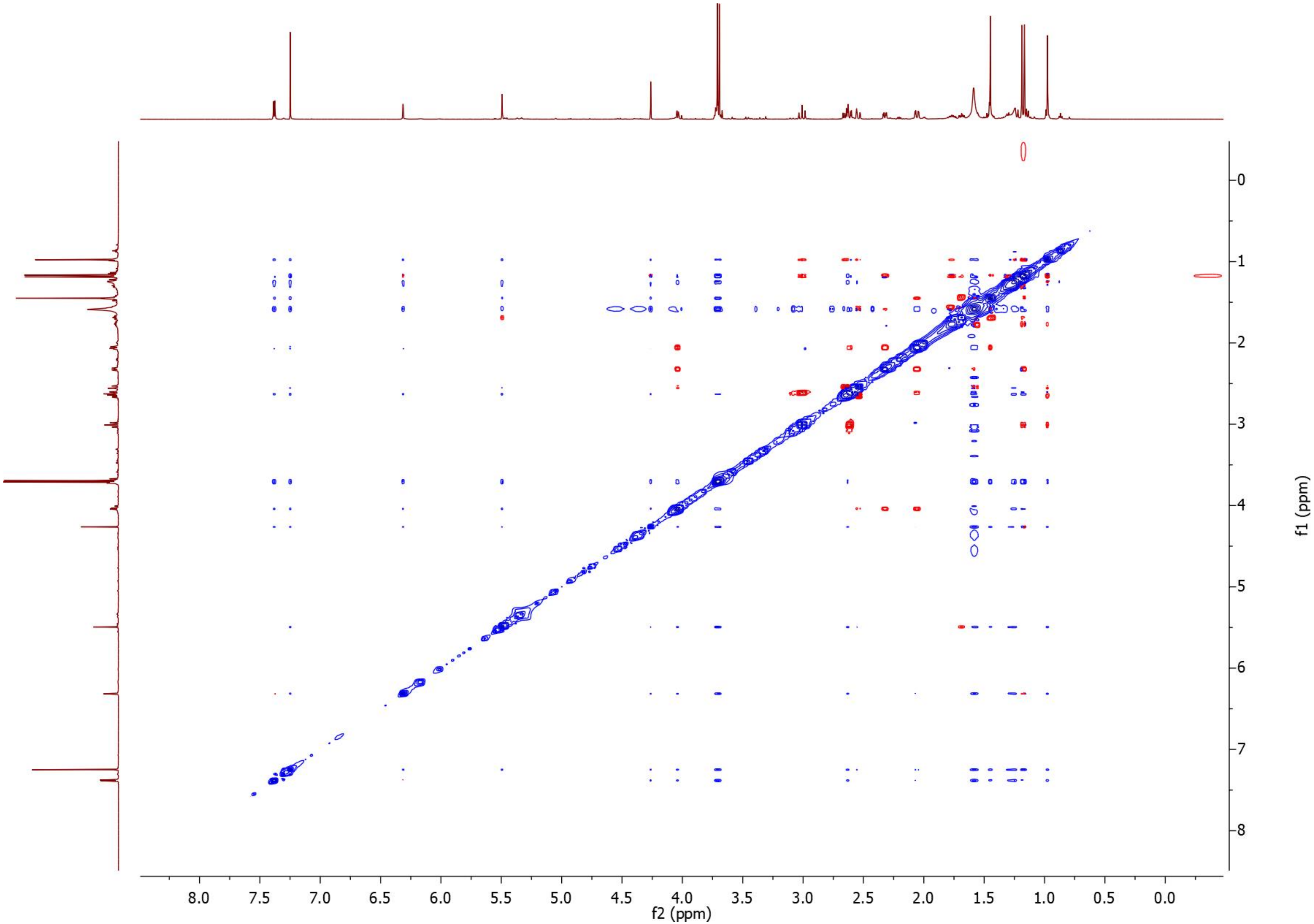

Figure S 10. ^{1}H-^{1}H-NOESY spectrum of compound **3** in $CDCl_3$

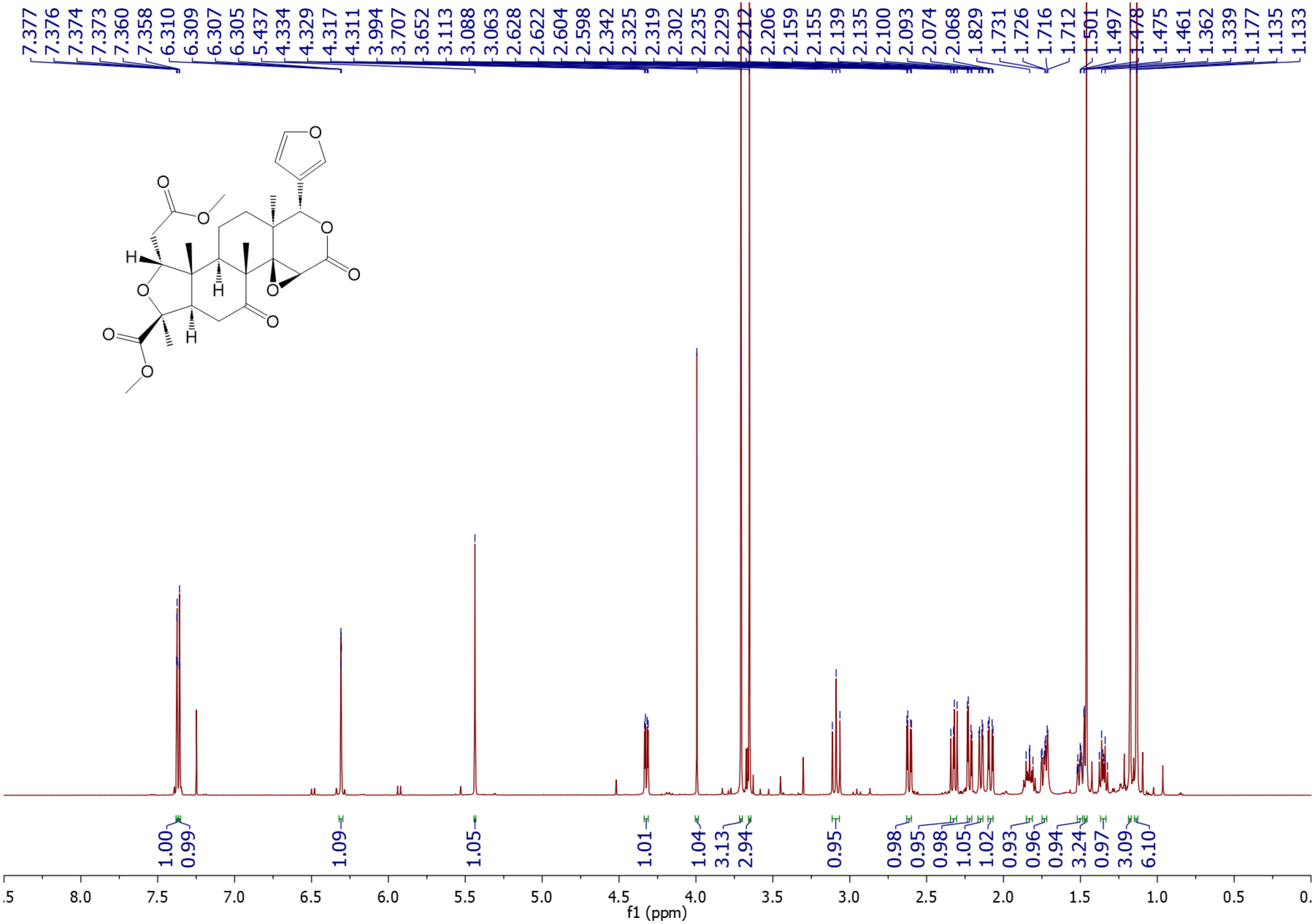

Figure S 11. ^{1}H-NMR (600 MHz) spectrum of compound **2** in $CDCl_3$

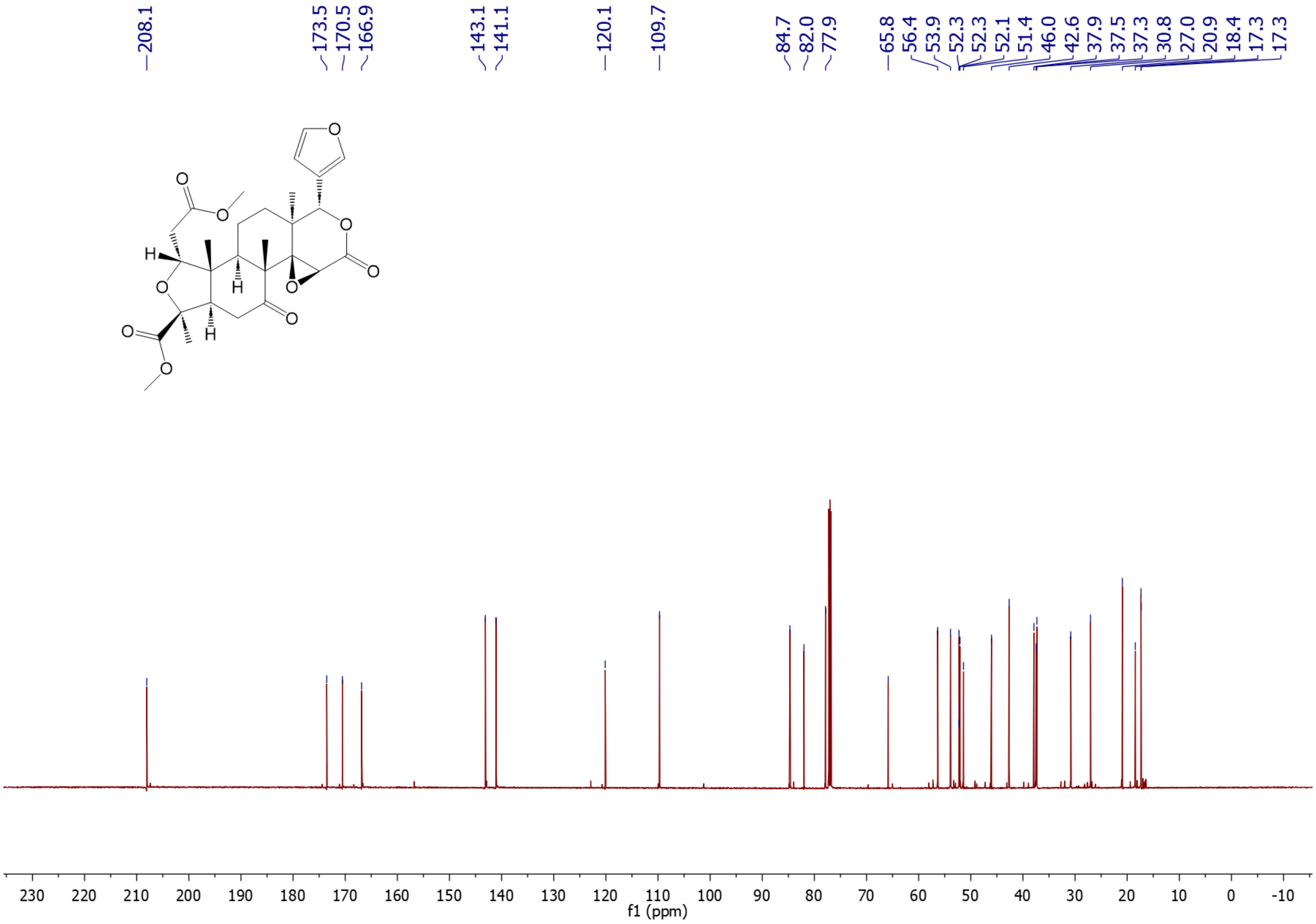

Figure S 12. ^{13}C-NMR (150 MHz) spectrum of compound **2** in $CDCl_3$

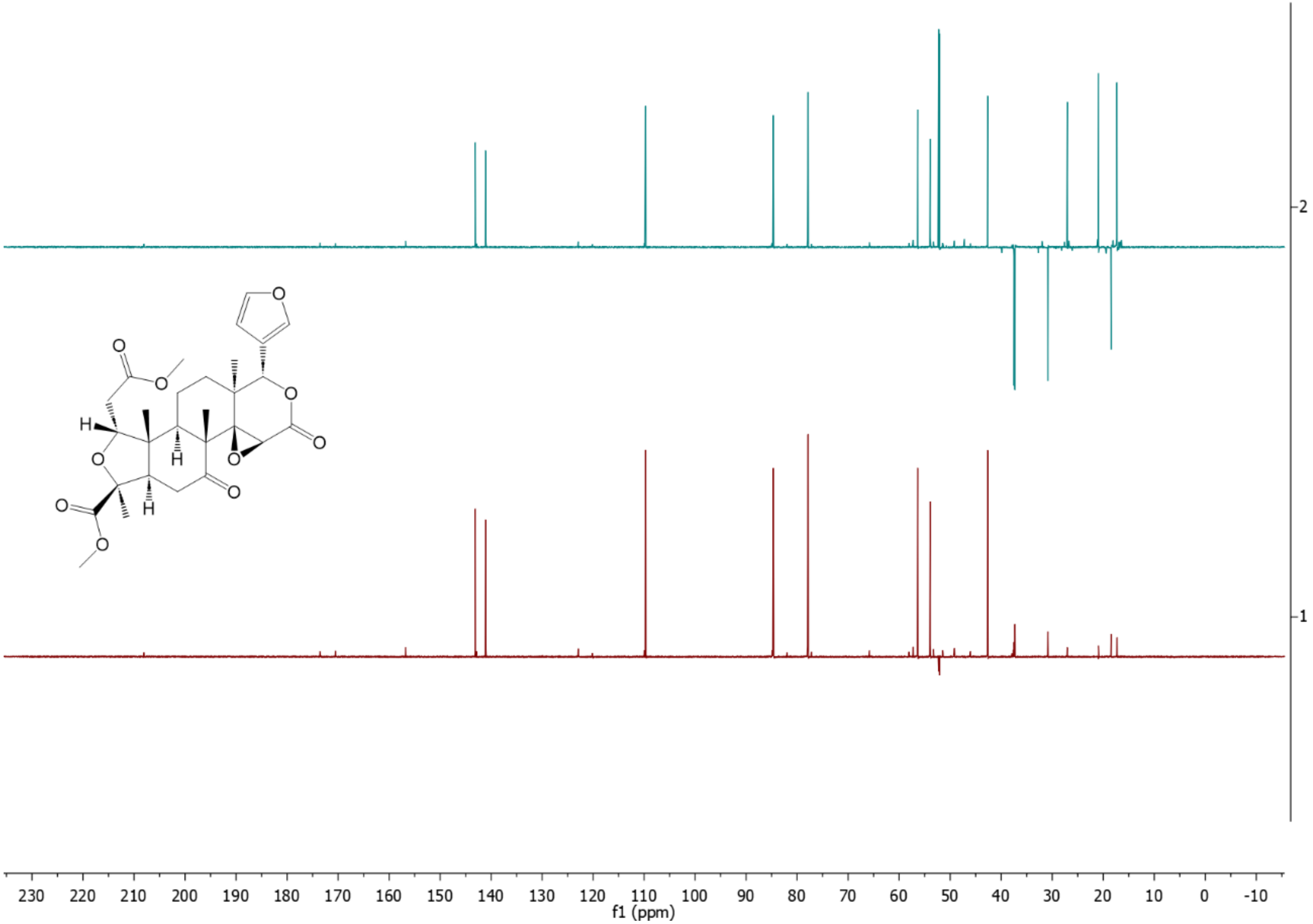

Figure S 13. DEPT-NMR (150 MHz) spectrum of compound **2** in $CDCl_3$

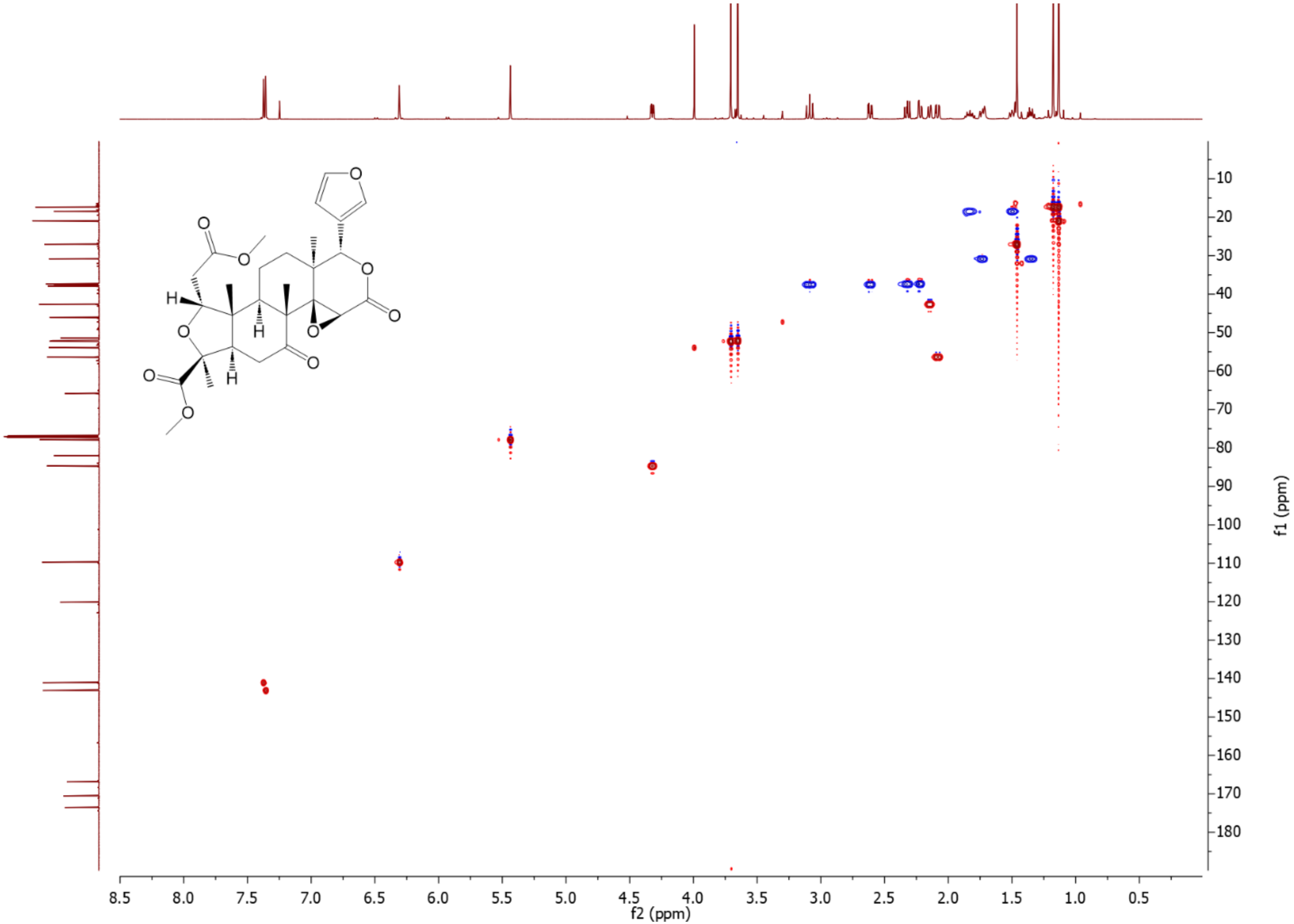

Figure S 14. HSQC spectrum of compound **2** in $CDCl_3$

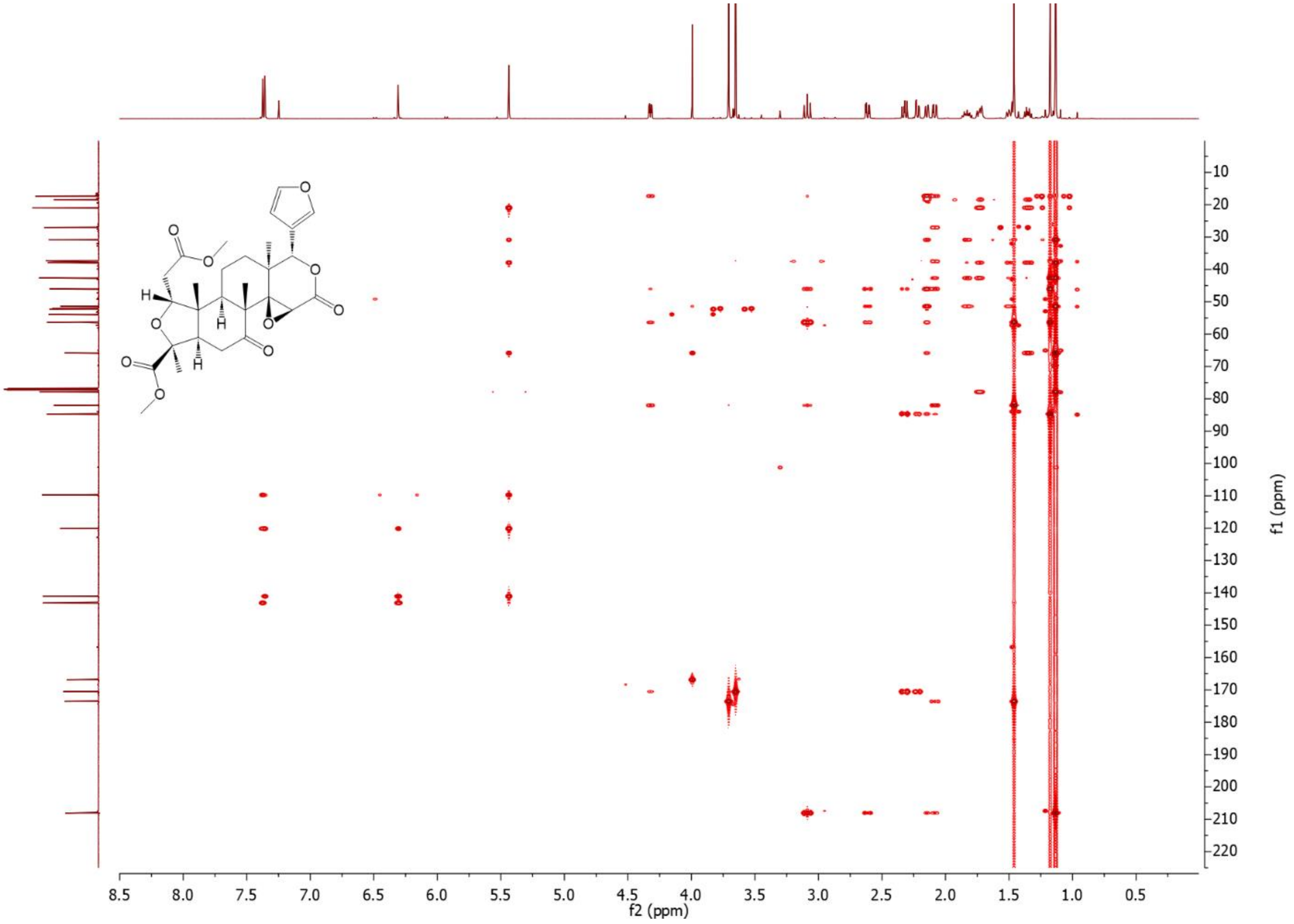

Figure S 15. HMBC spectrum of compound **2** in $CDCl_3$

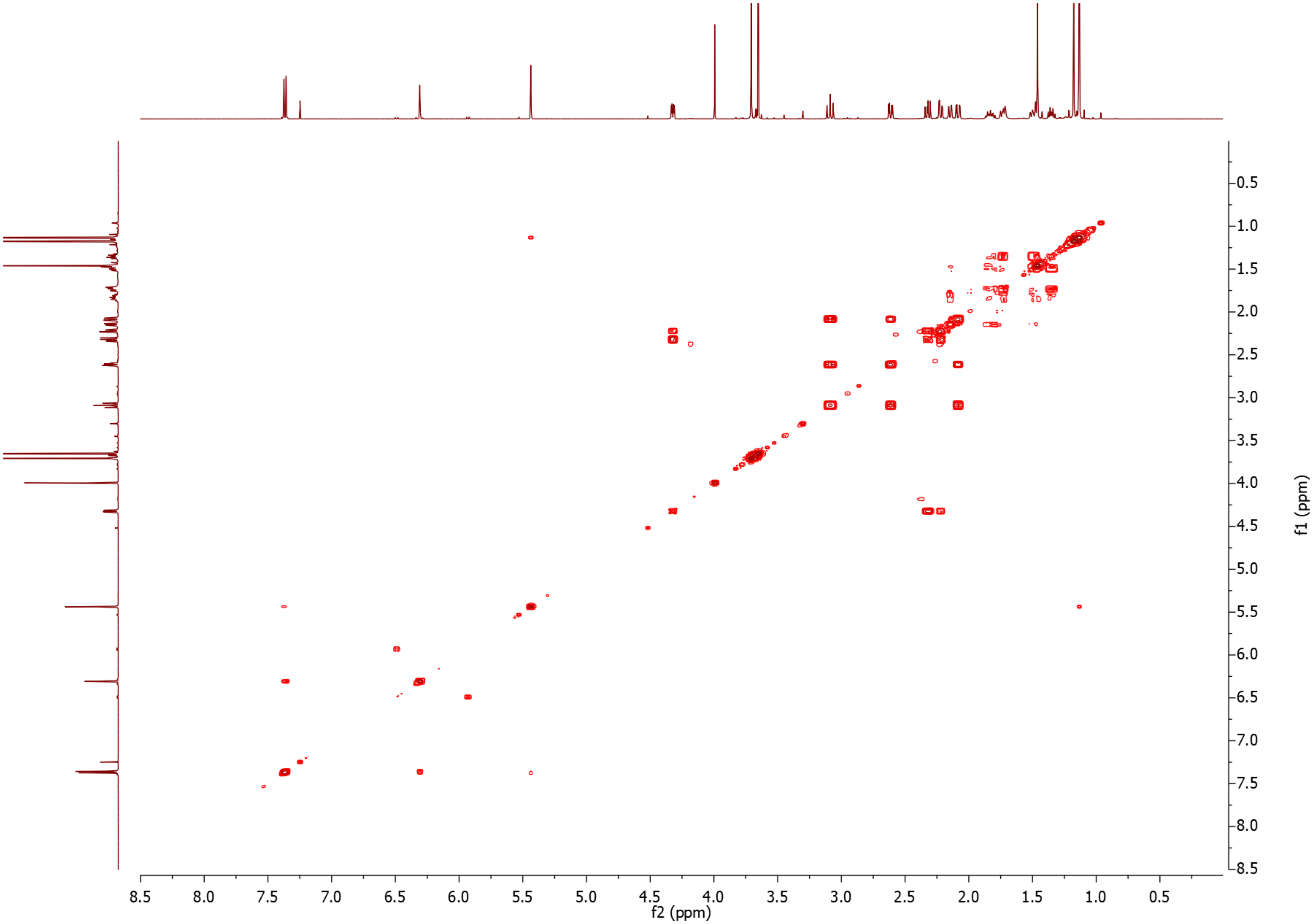

Figure S 16. ^{1}H-^{1}H-COSY spectrum of compound **2** in $CDCl_3$

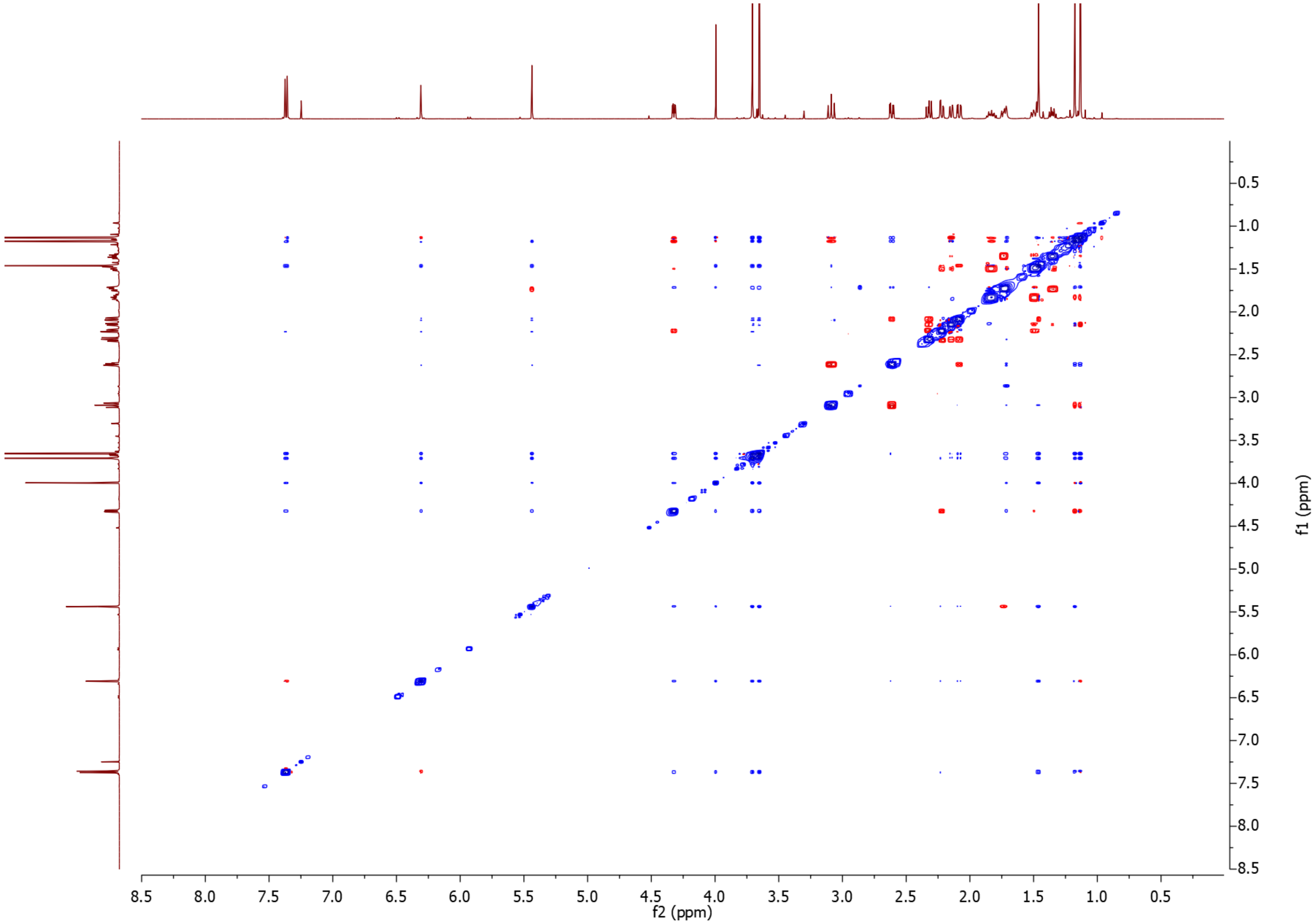

Figure S 17. ^{1}H-^{1}H-NOESY spectrum of compound **2** in $CDCl_3$

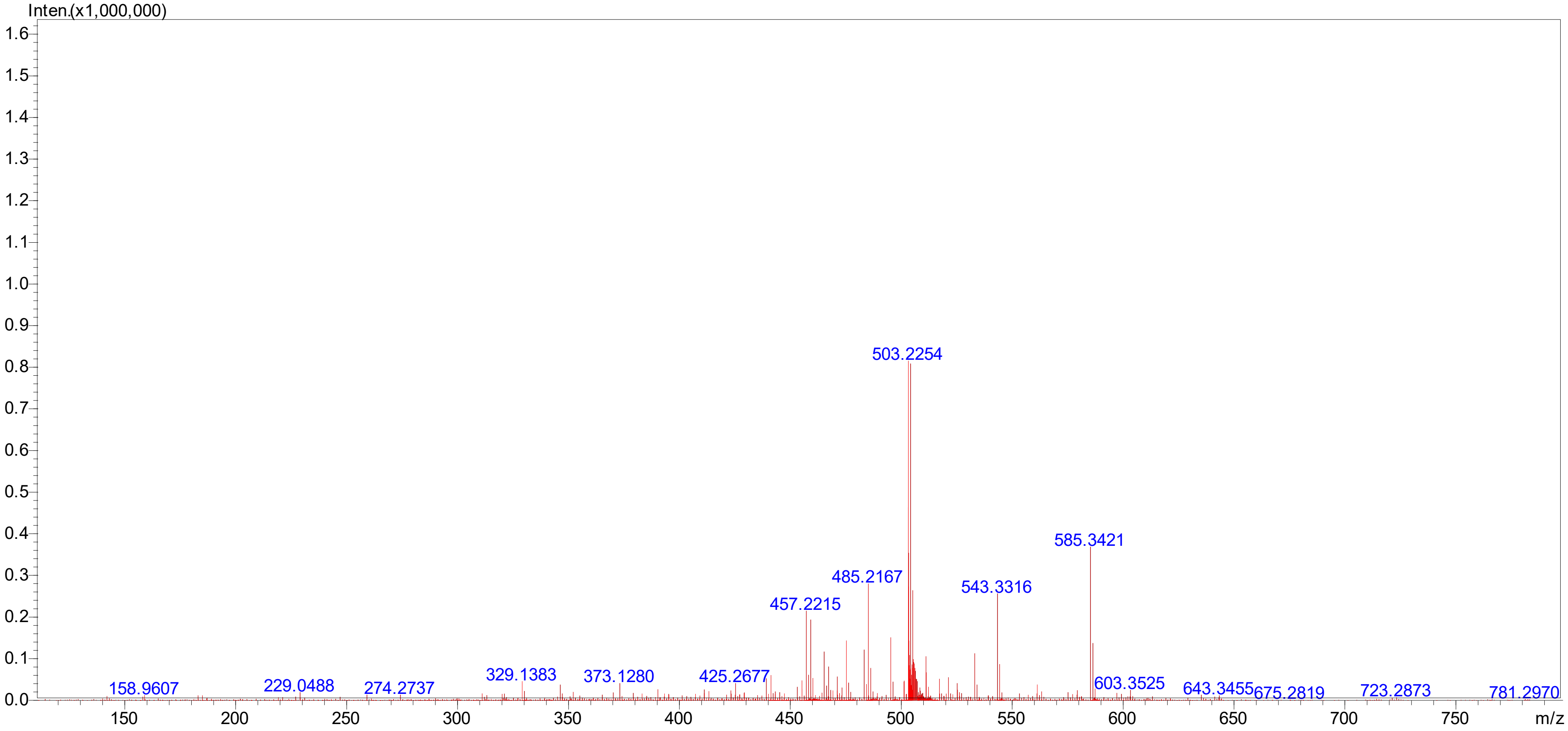

Figure S 18. HR-ESI-MS spectrum of compound **4**

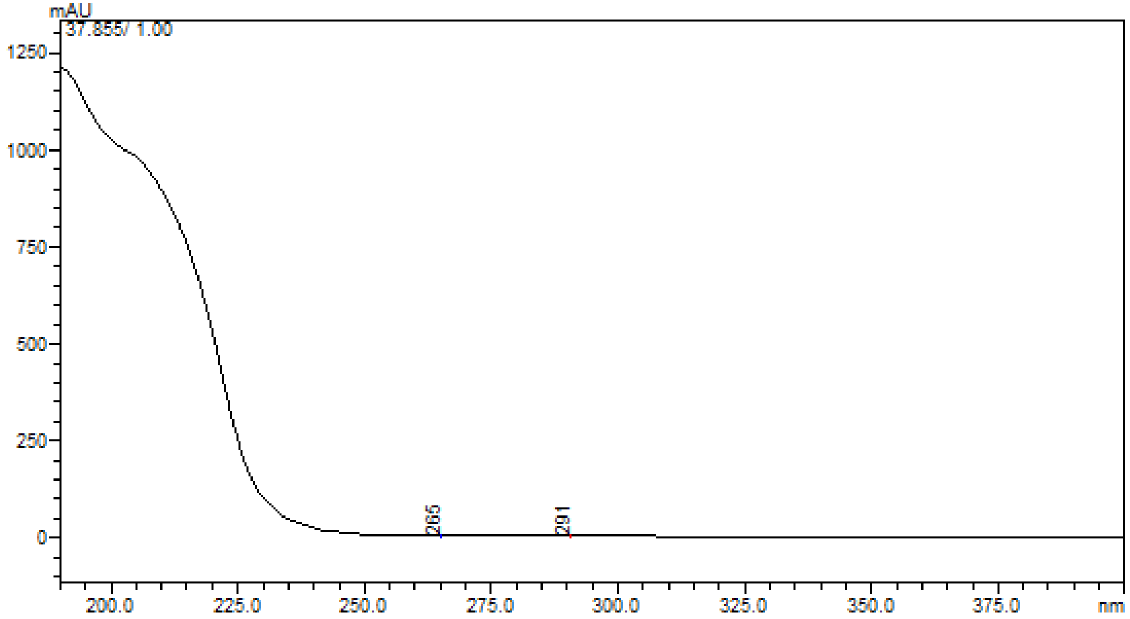

Figure S 19. UV spectrum of compound **4** in methanol

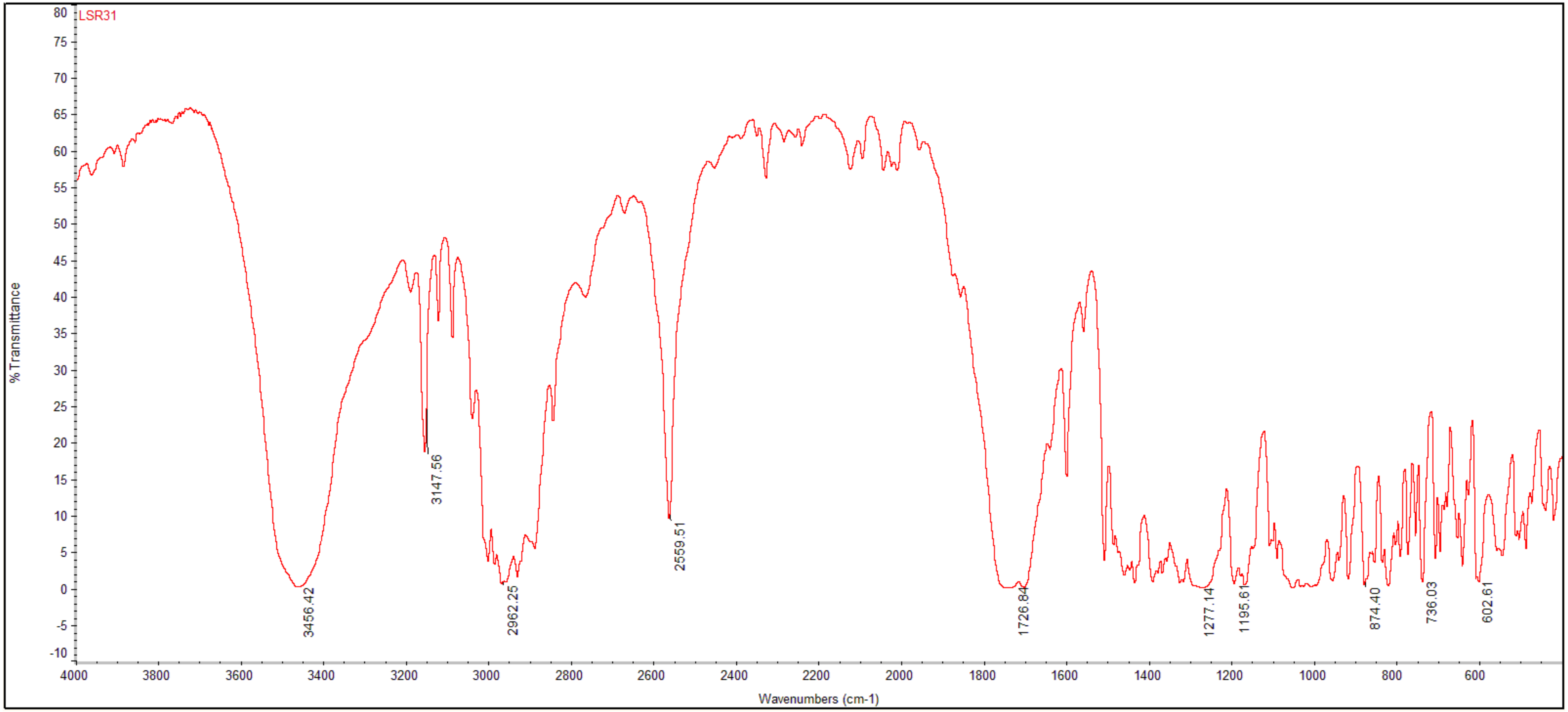

Figure S 20. IR spectrum (film on KBr plates) of compound **4**

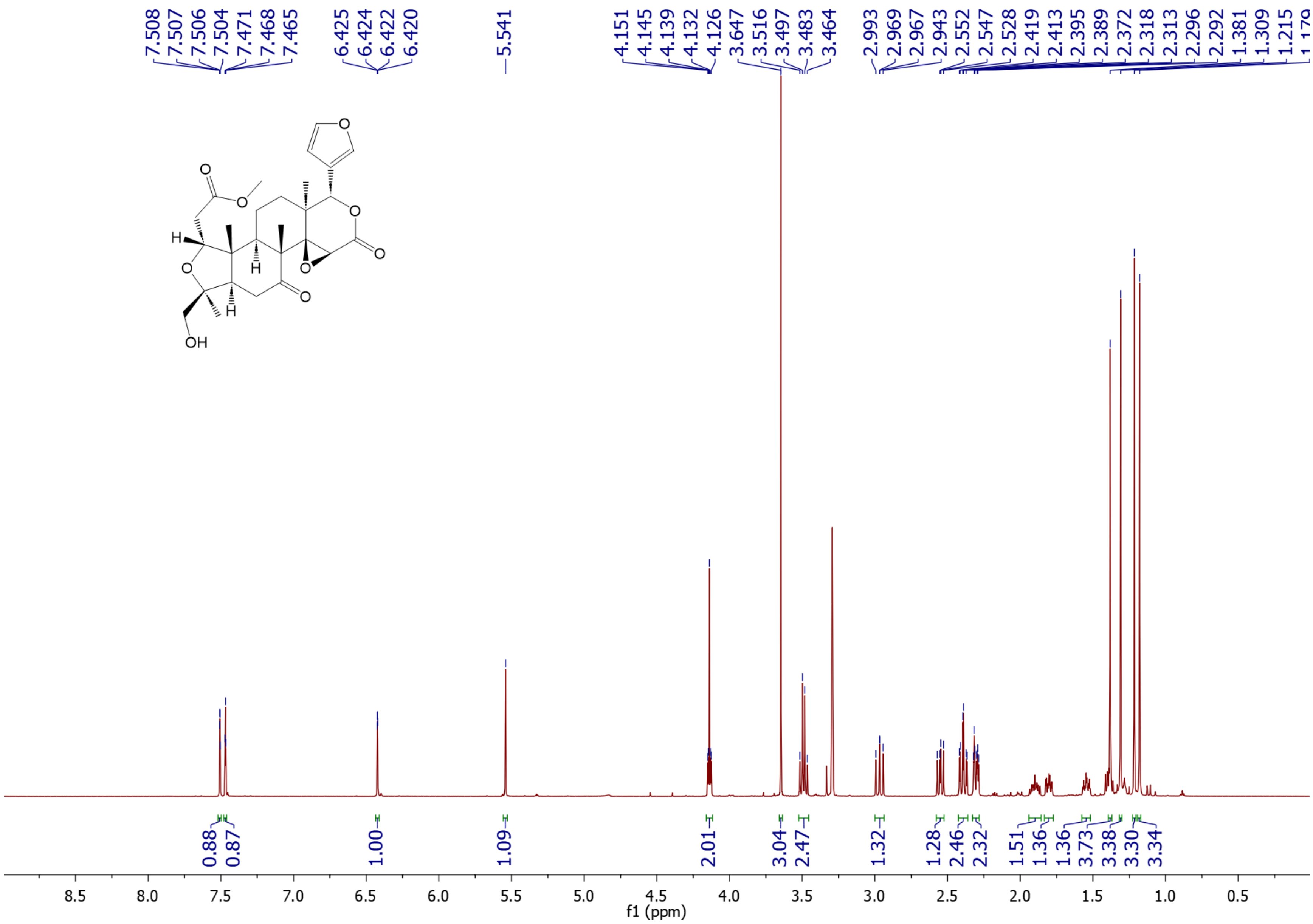

Figure S 21. ^{1}H-NMR (600 MHz) spectrum of compound **4** in CD_3OD

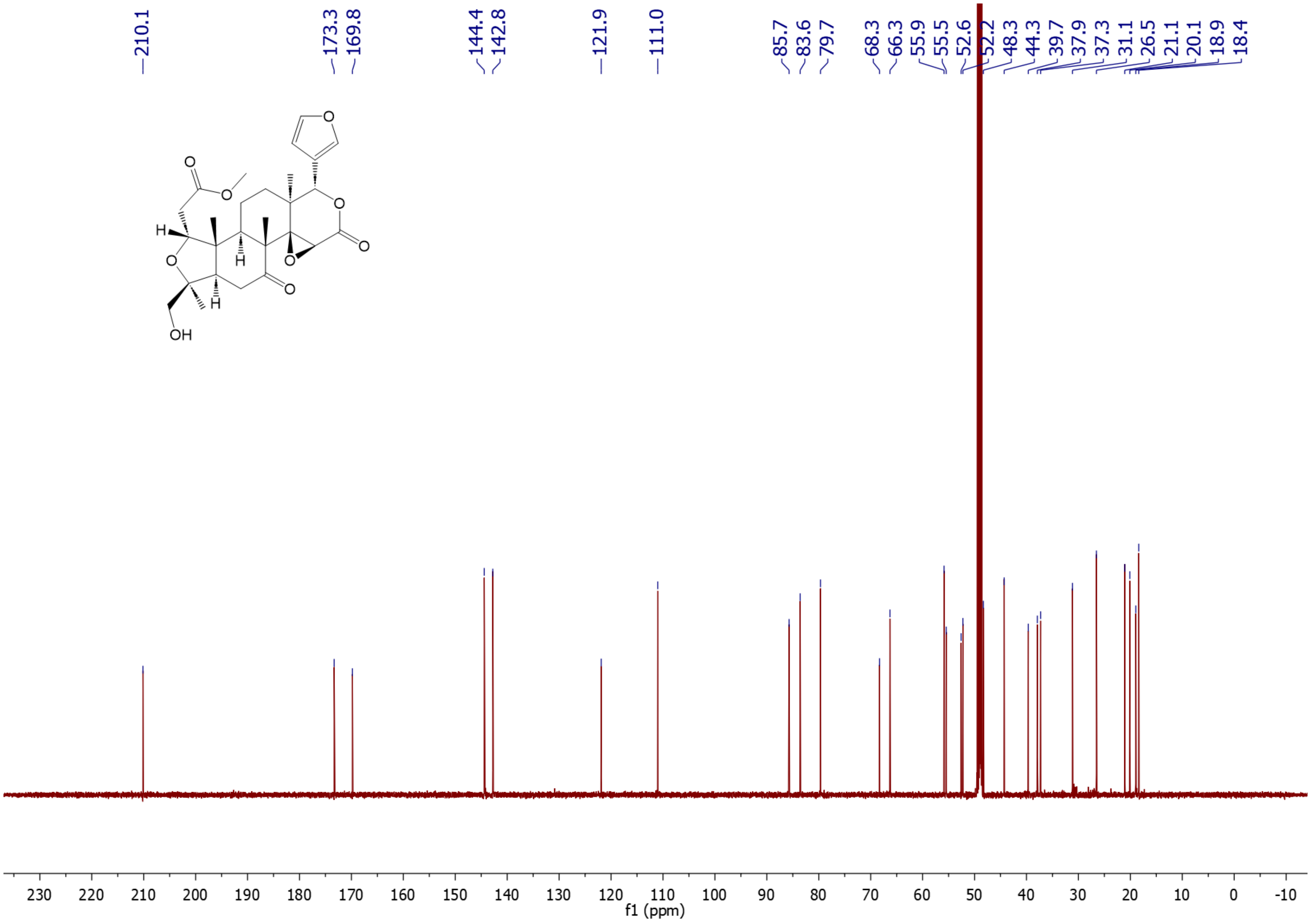

Figure S 22. ^{13}C-NMR (150 MHz) spectrum of compound **4** in CD_3OD

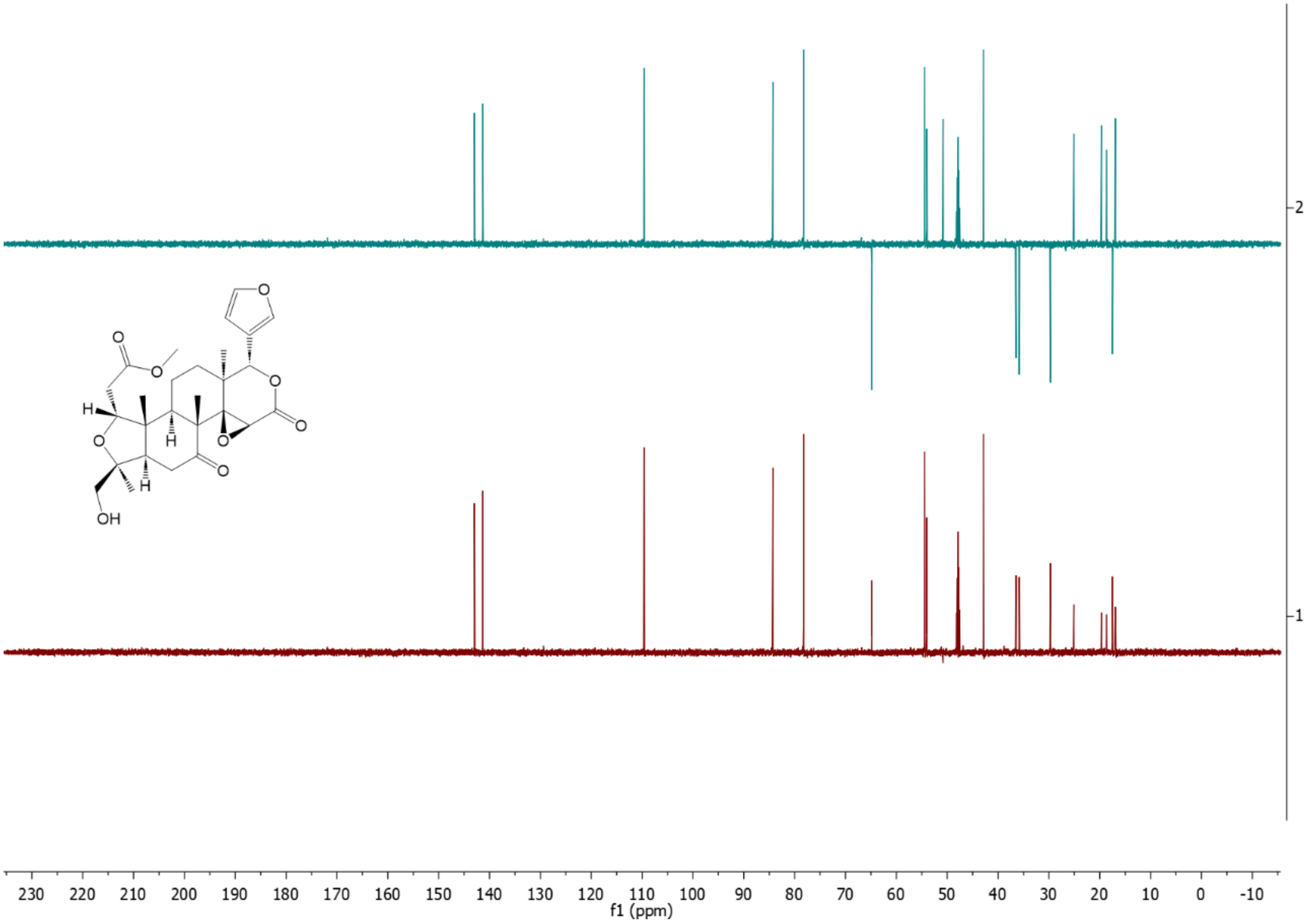

Figure S 23. DEPT-NMR (150 MHz) spectrum of compound **4** in CD_3OD

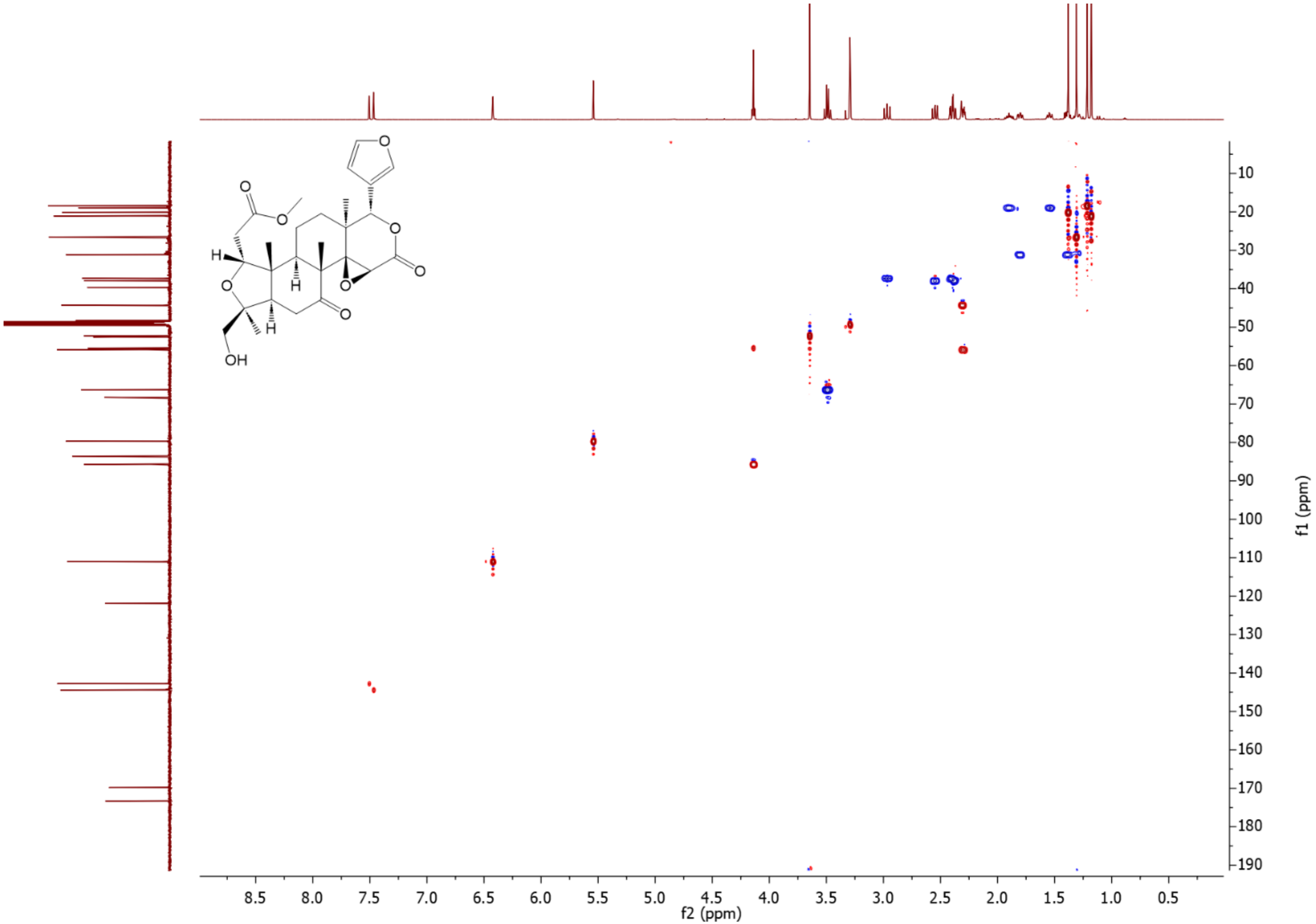

Figure S 24. HSQC spectrum of compound **4** in CD_3OD

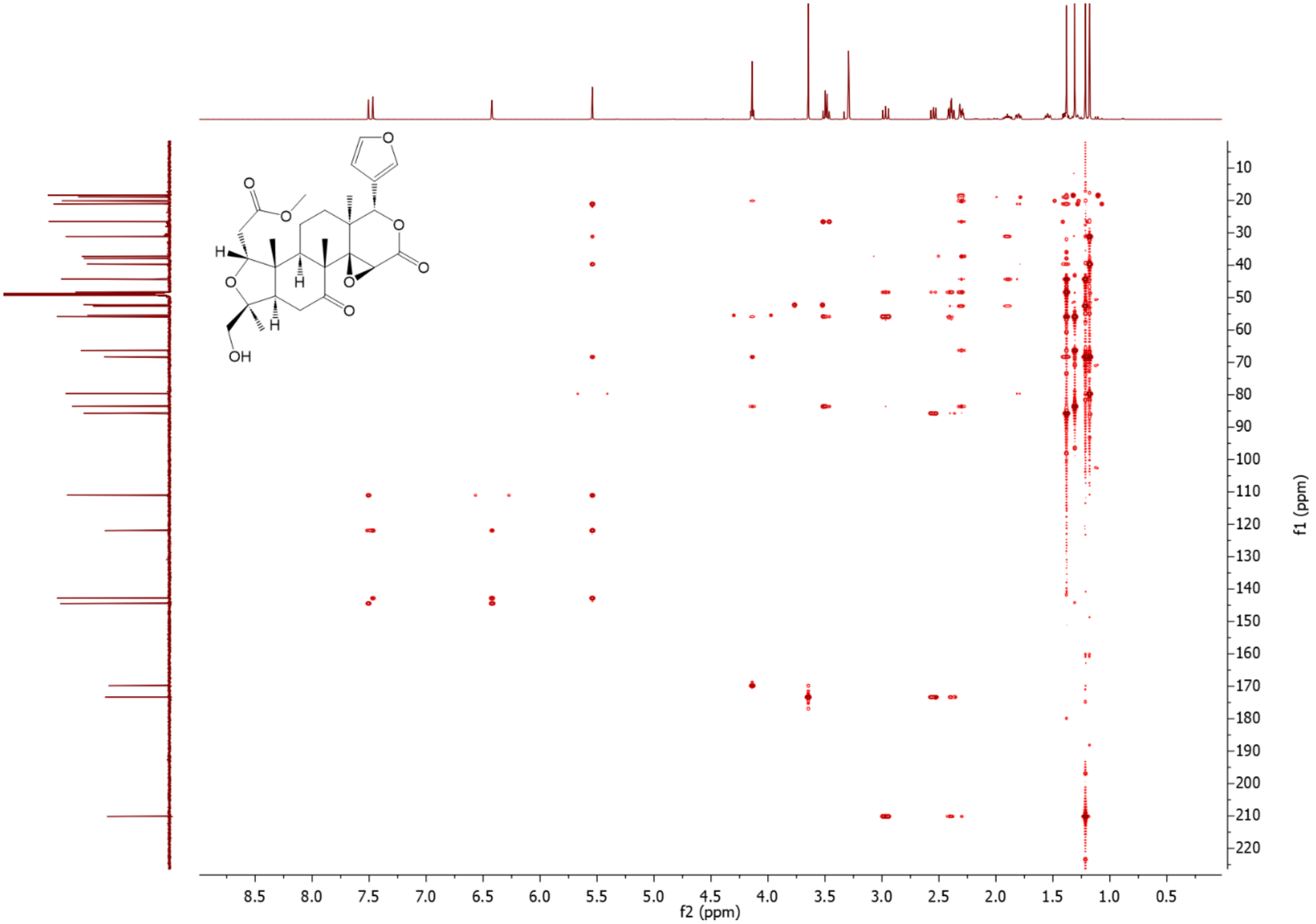

Figure S 25. HMBC spectrum of compound **4** in CD_3OD

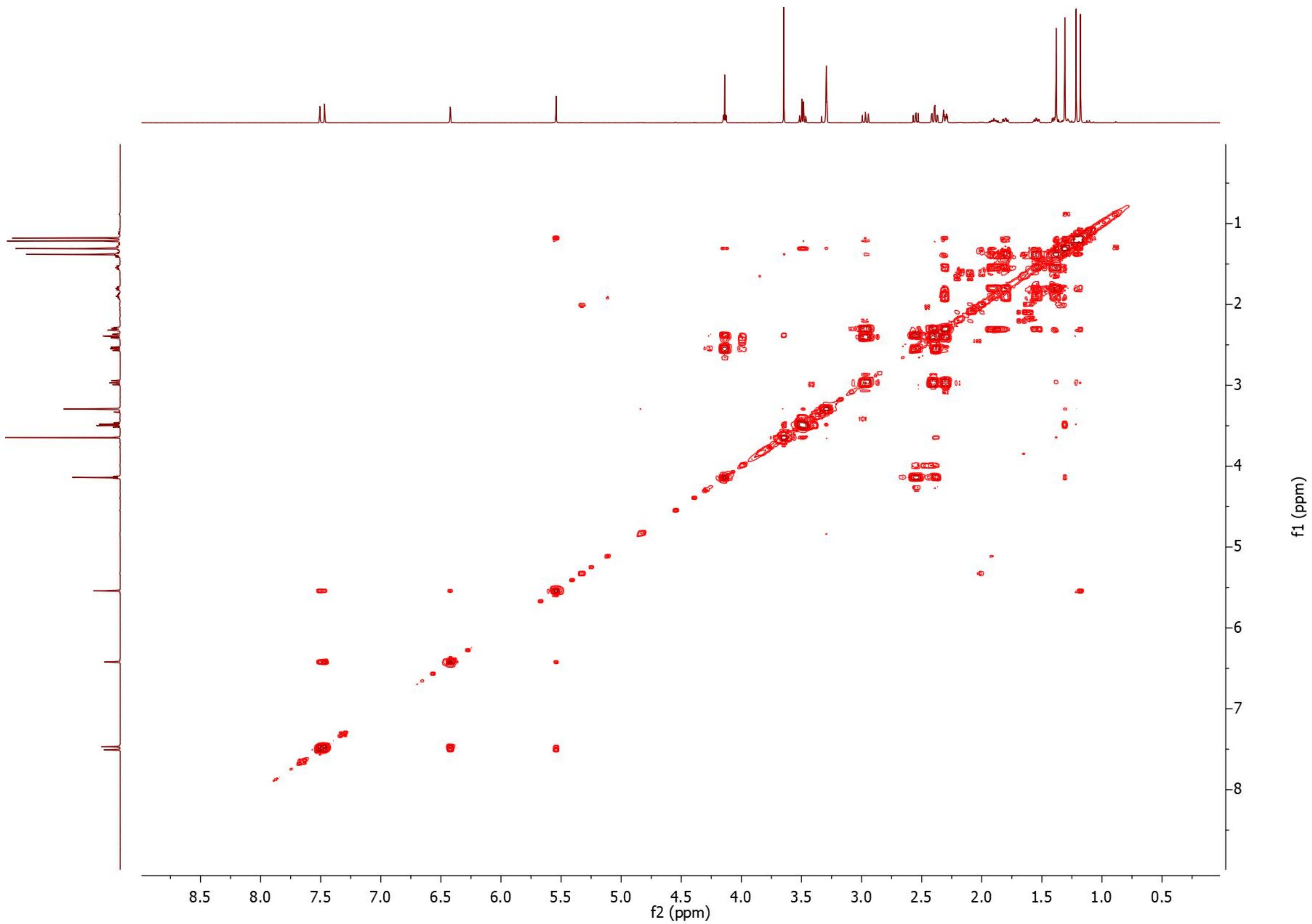

Figure S 26. ^{1}H-^{1}H-COSY spectrum of compound **4** in CD_3OD

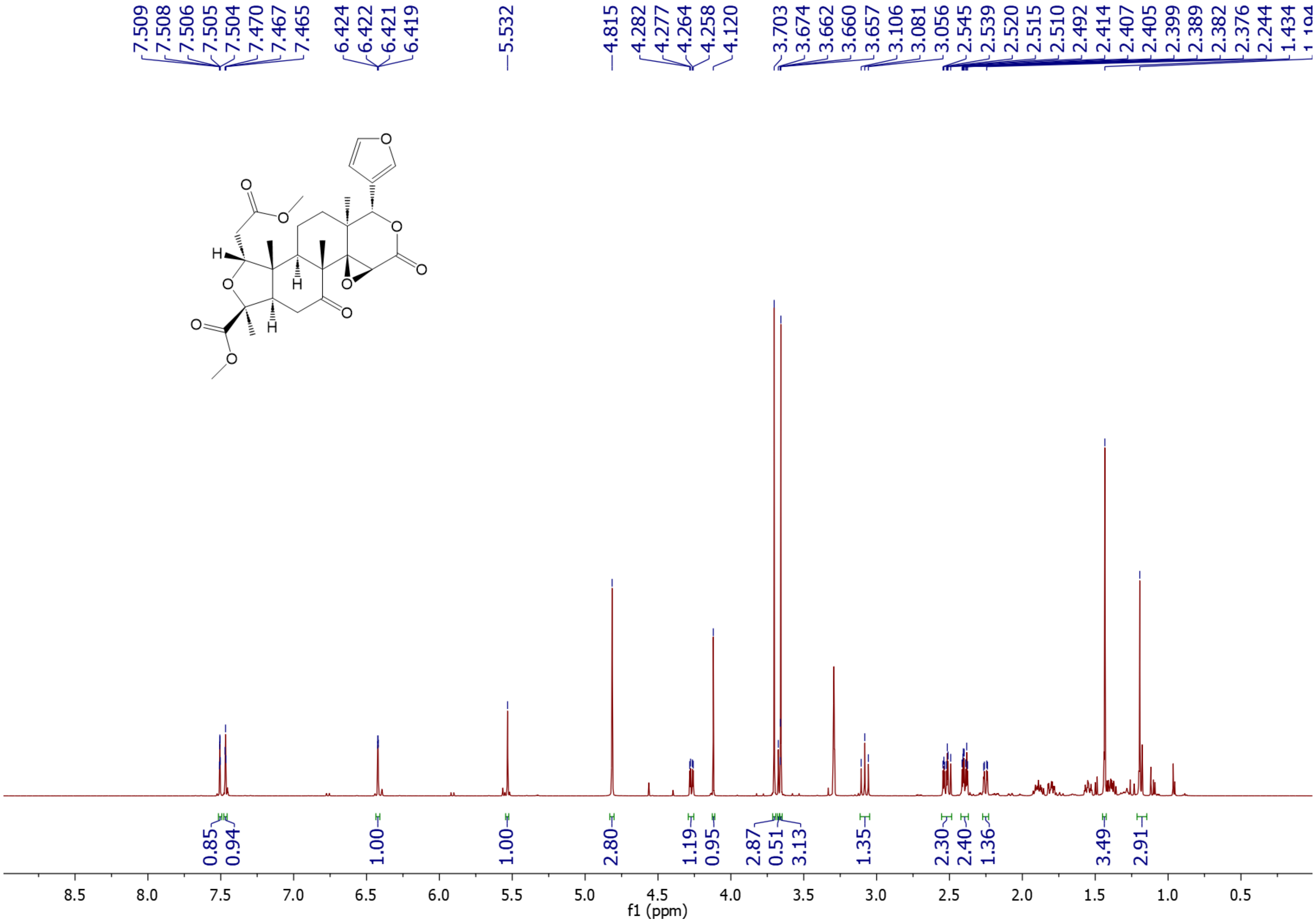

Figure S 27. ^{1}H-NMR (600 MHz) spectrum of compound **2** in CD_3OD

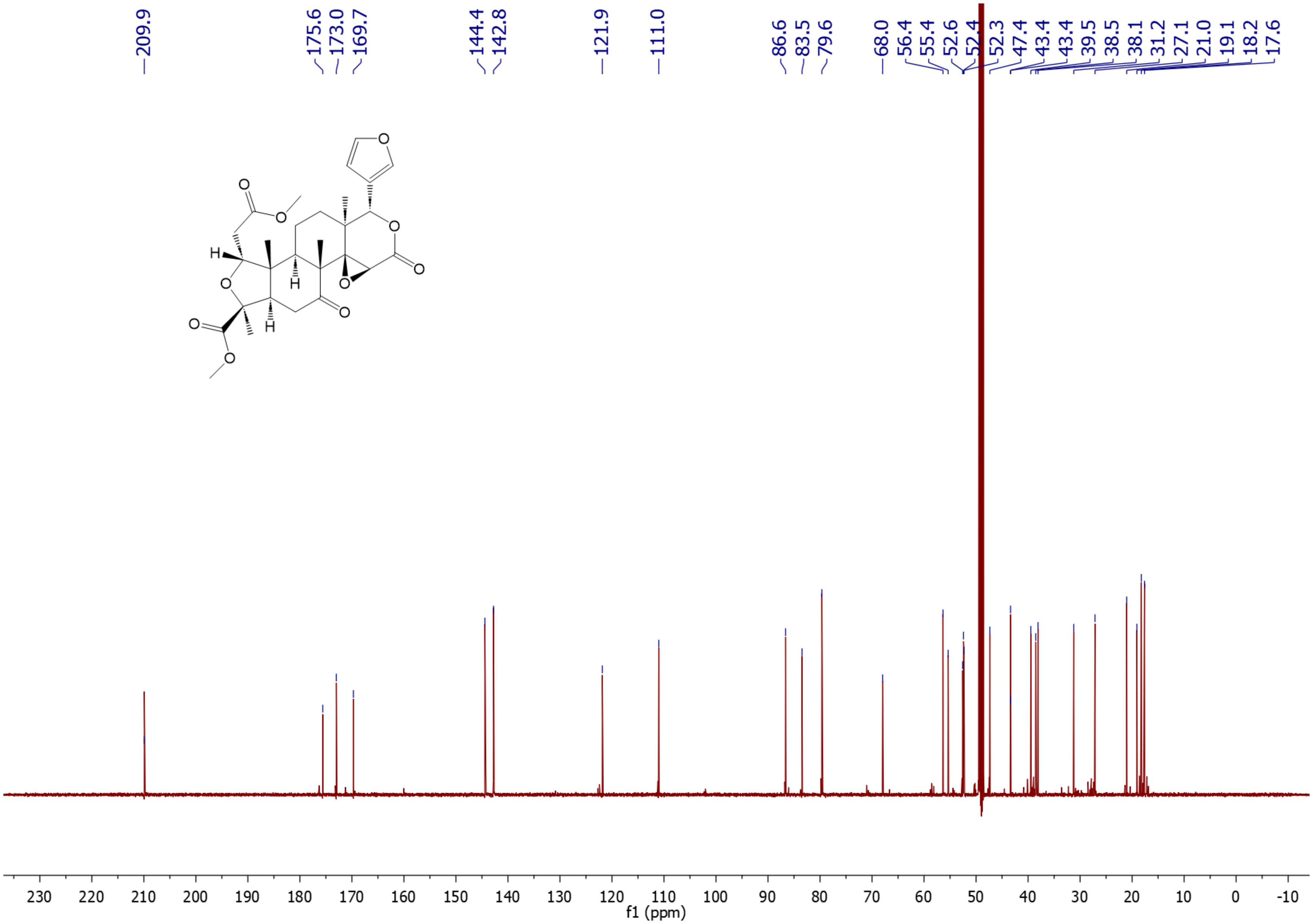

Figure S 28. ^{13}C-NMR (150 MHz) spectrum of compound **2** in CD_3OD

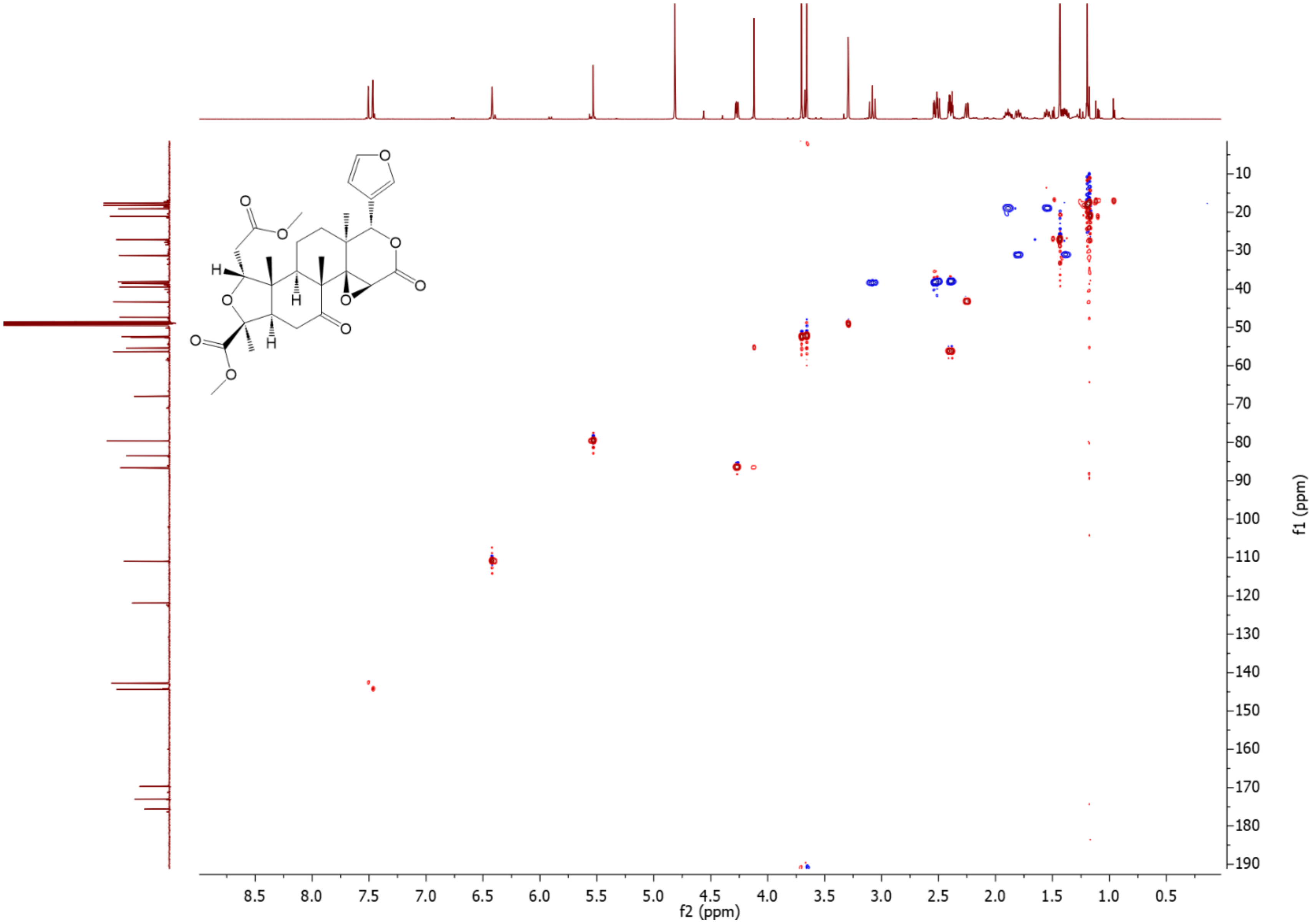

Figure S 29. HSQC spectrum of compound **2** in CD_3OD

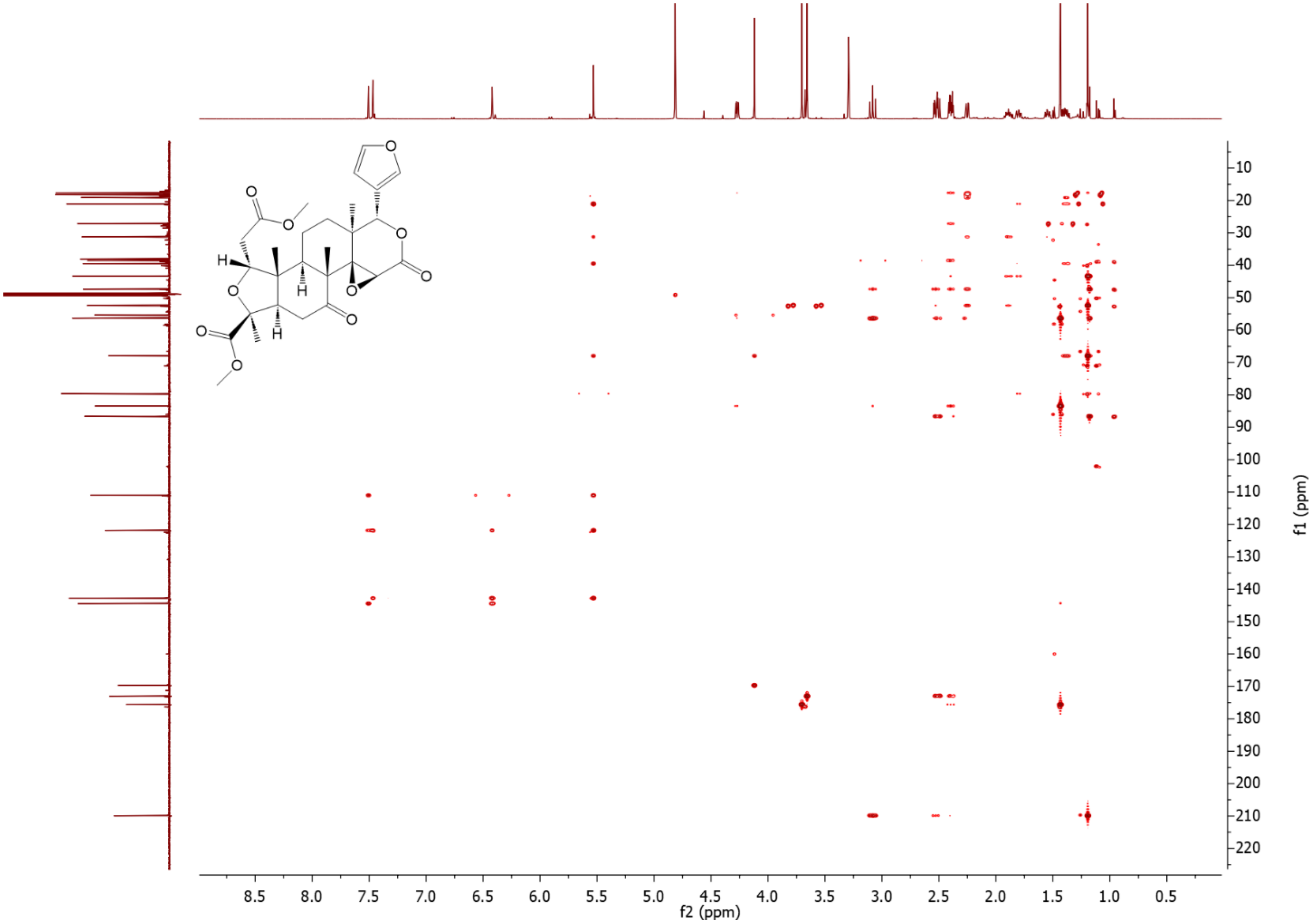

Figure S 30. HMBC spectrum of compound **2** in CD_3OD

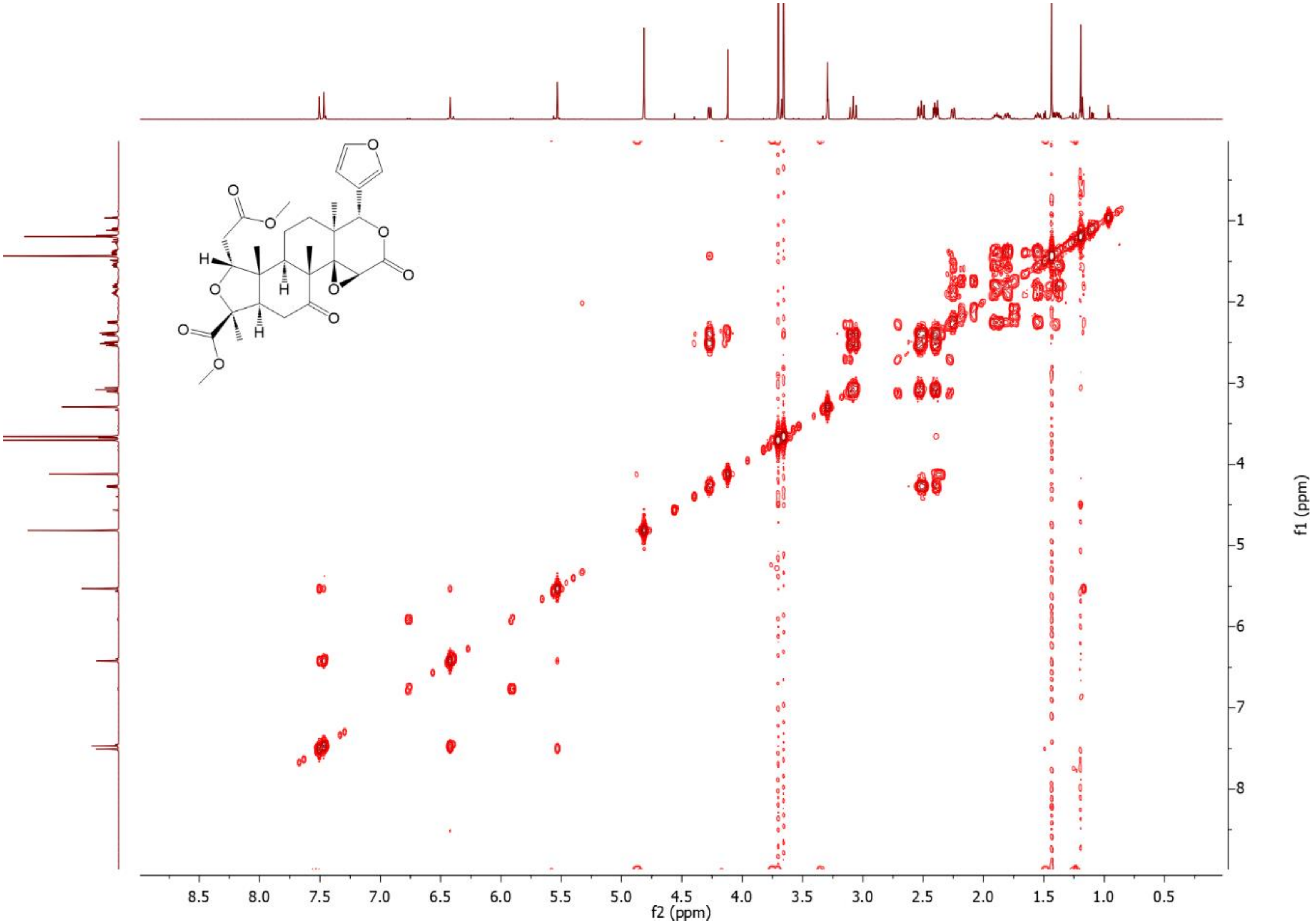

Figure S 31. ^{1}H-^{1}H-COSY spectrum of compound **2** in CD_3OD

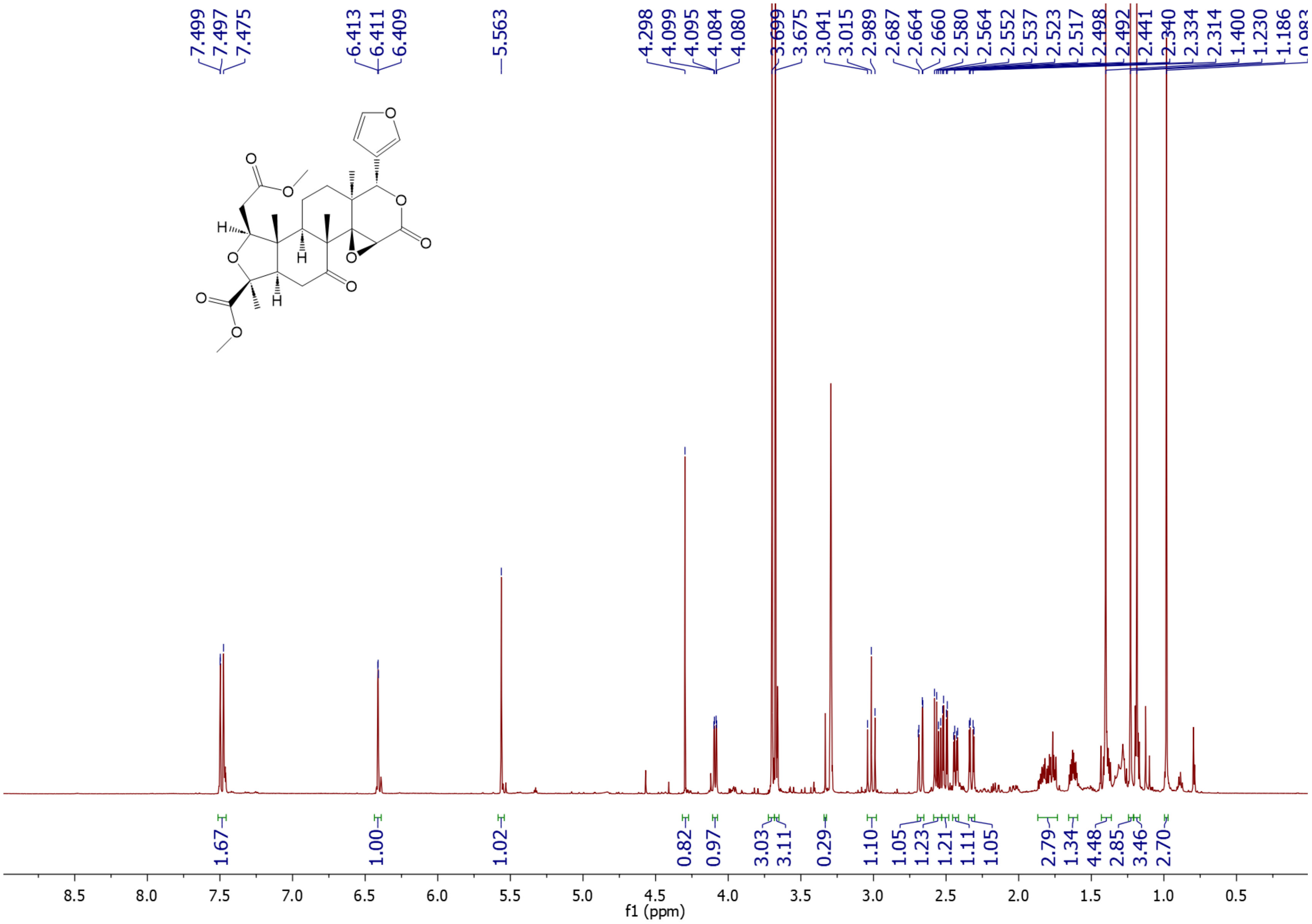

Figure S 32. ^{1}H-NMR (600 MHz) spectrum of compound **3** in CD_3OD

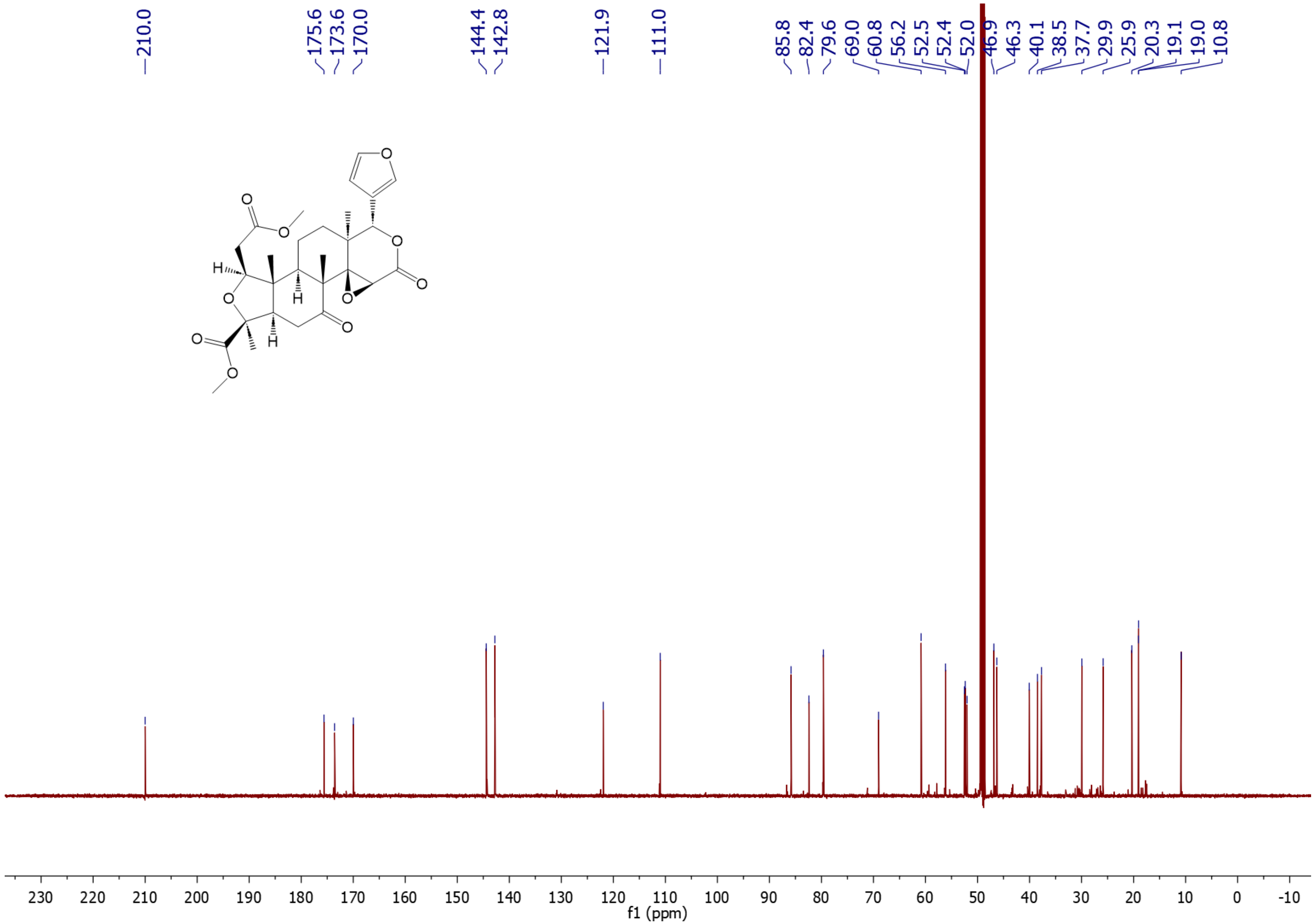

Figure S 33. ^{13}C-NMR (150 MHz) spectrum of compound **3** in CD_3OD

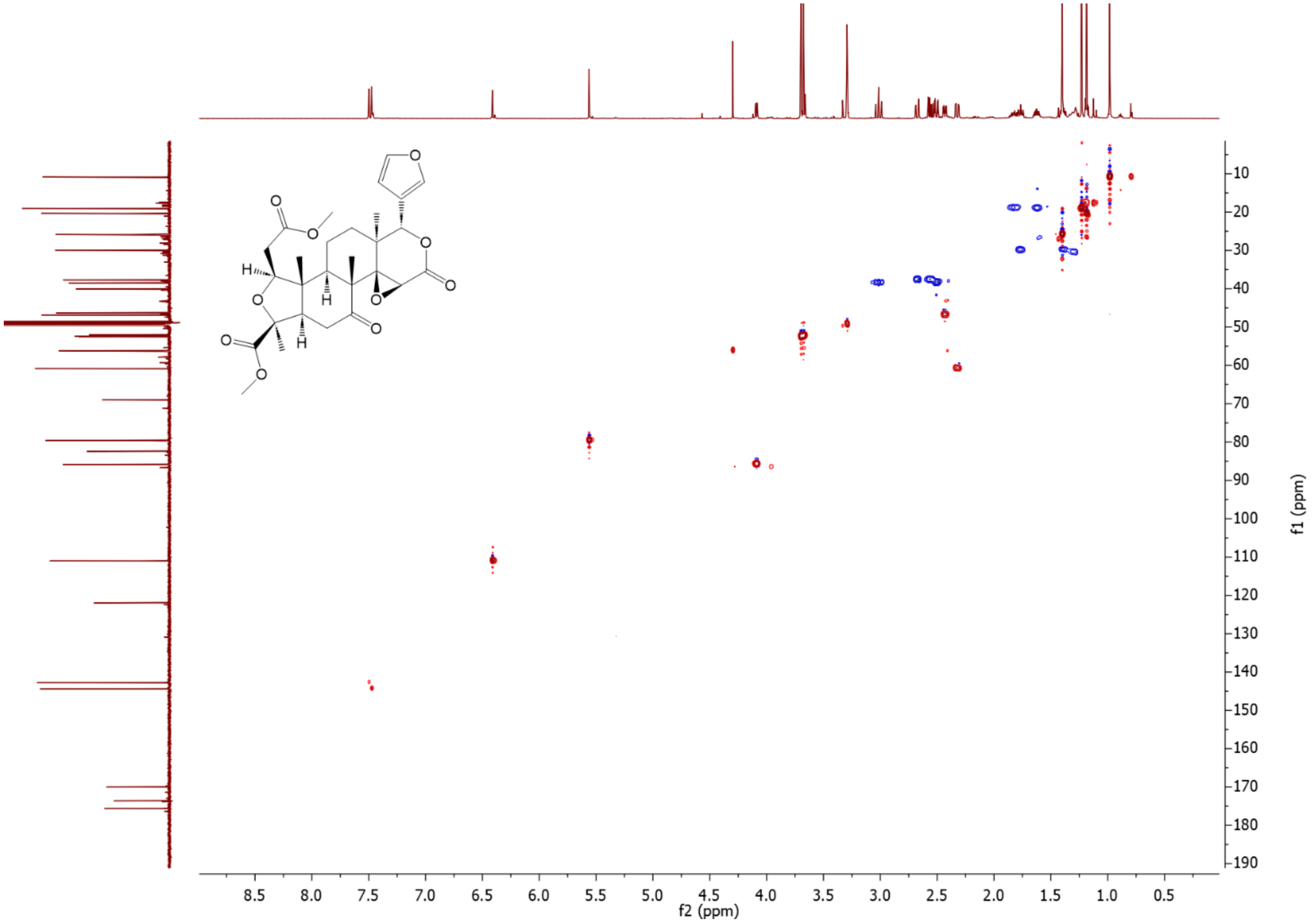

Figure S 34. HSQC spectrum of compound **3** in CD_3OD

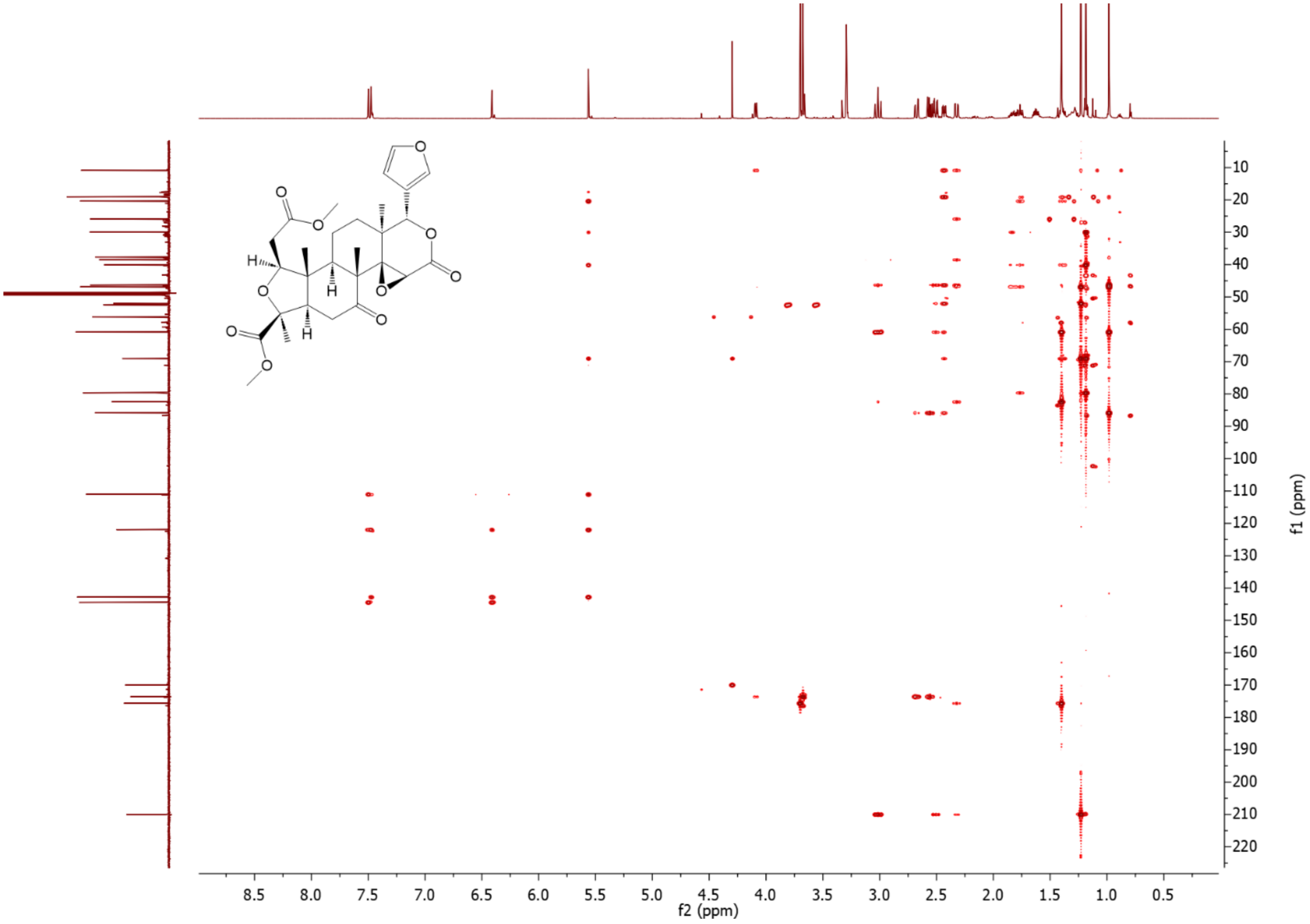

Figure S 35. HMBC spectrum of compound **3** in CD_3OD

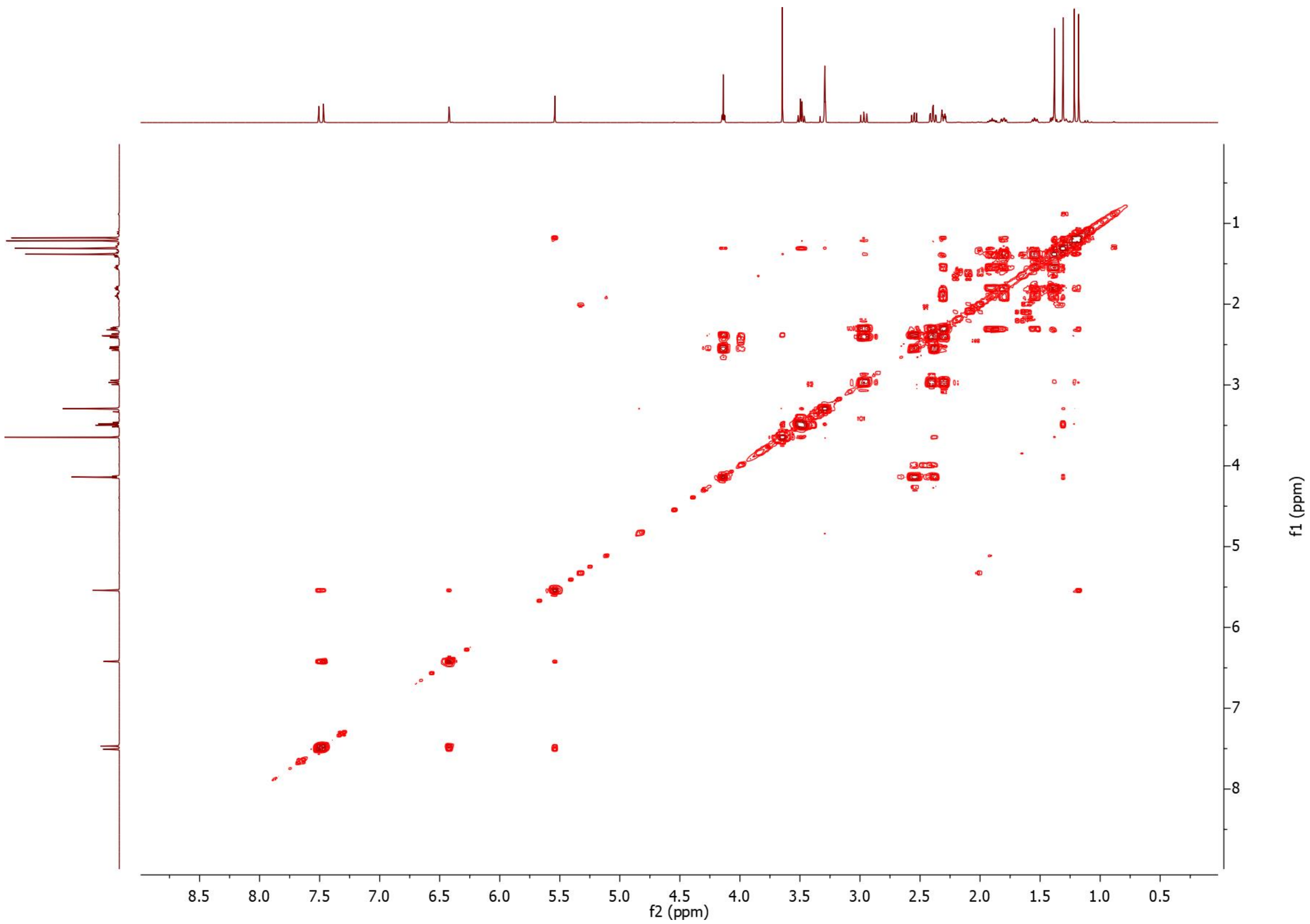

Figure S 36. ^1H-^1H-COSY spectrum of compound **3** in CD_3OD

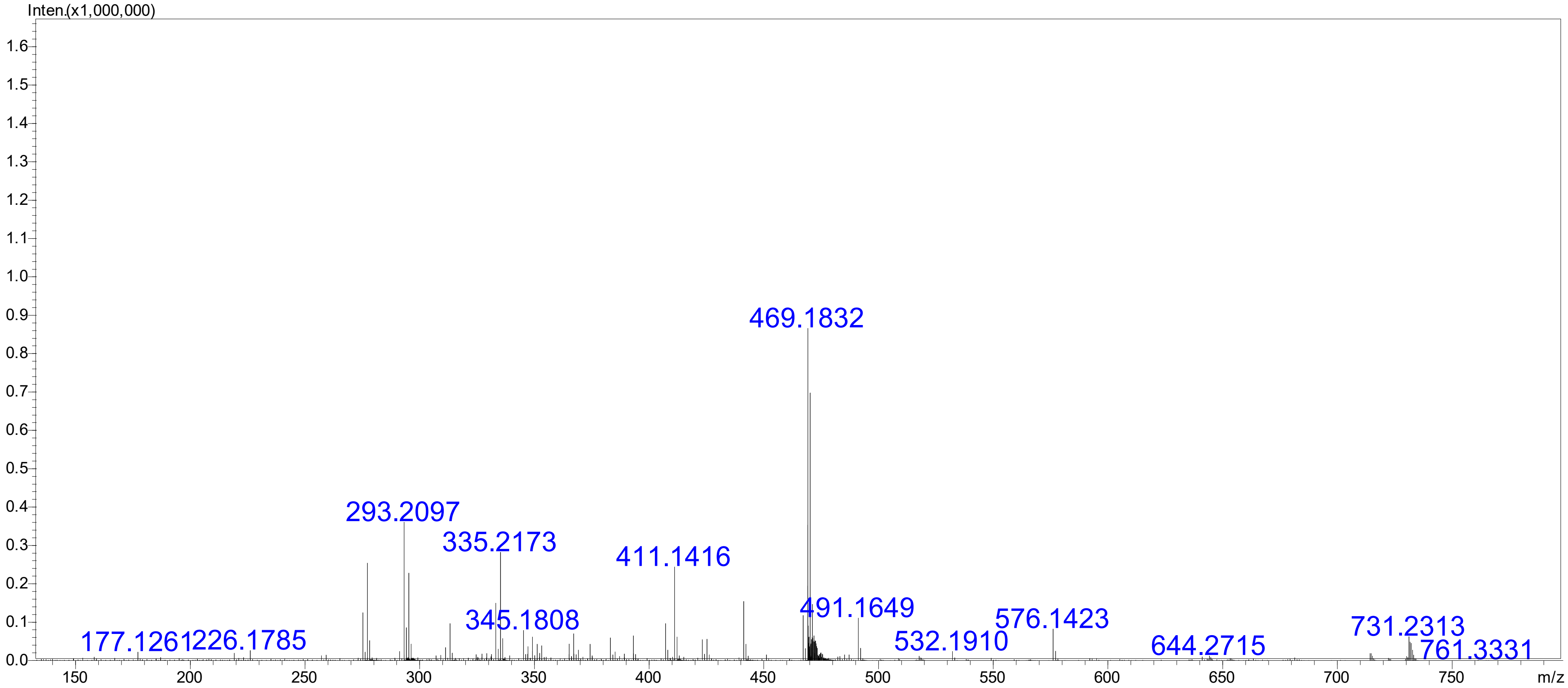

Figure S 37. HR-ESI-MS spectrum of compound **8**

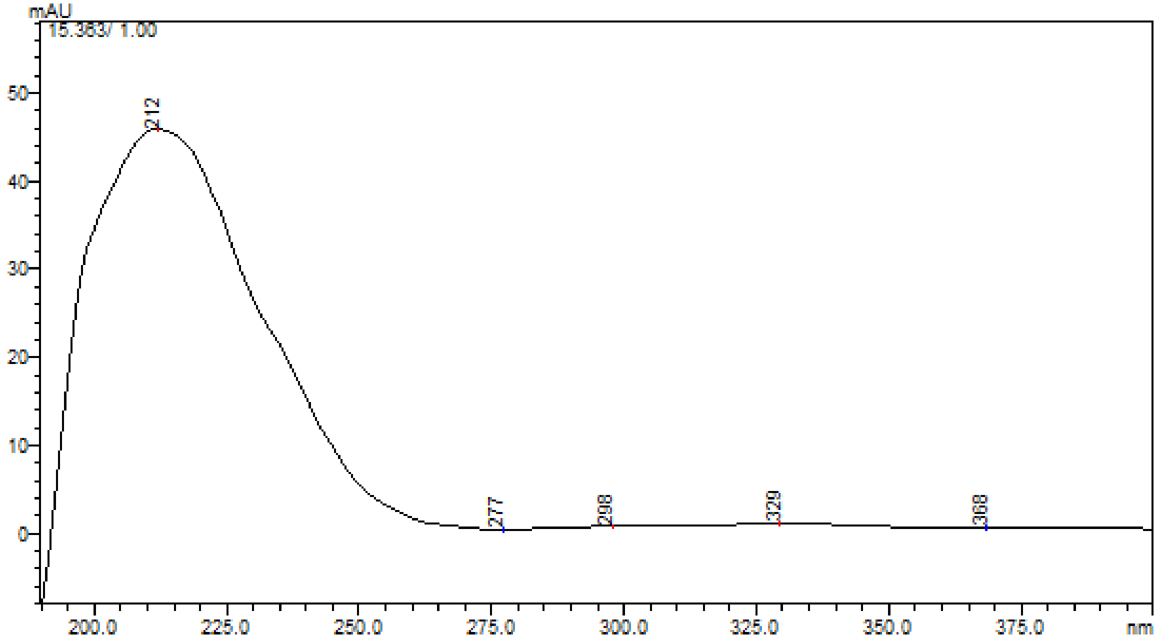

Figure S 38. UV spectrum of compound **8** in methanol

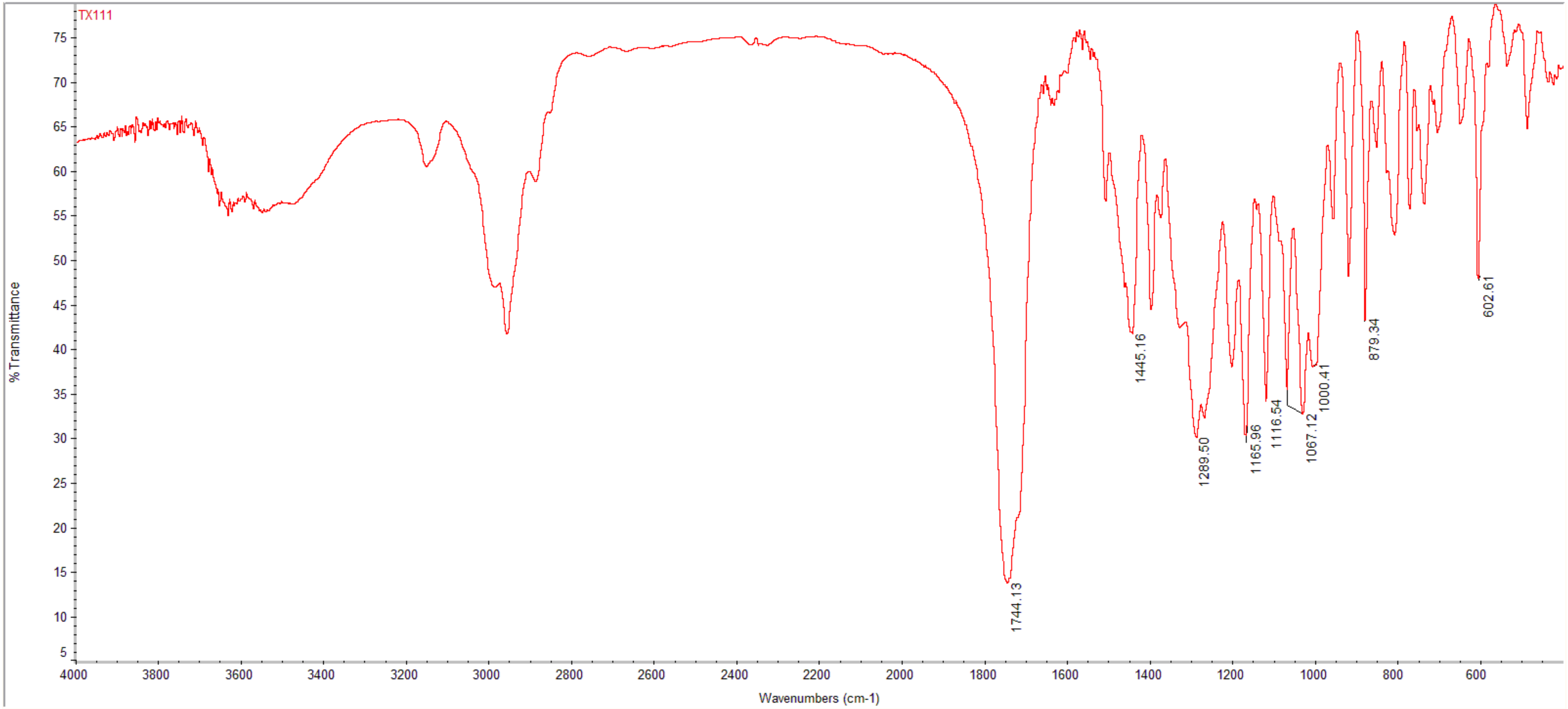

Figure S 39. IR spectrum (film on KBr plates) of compound **8**

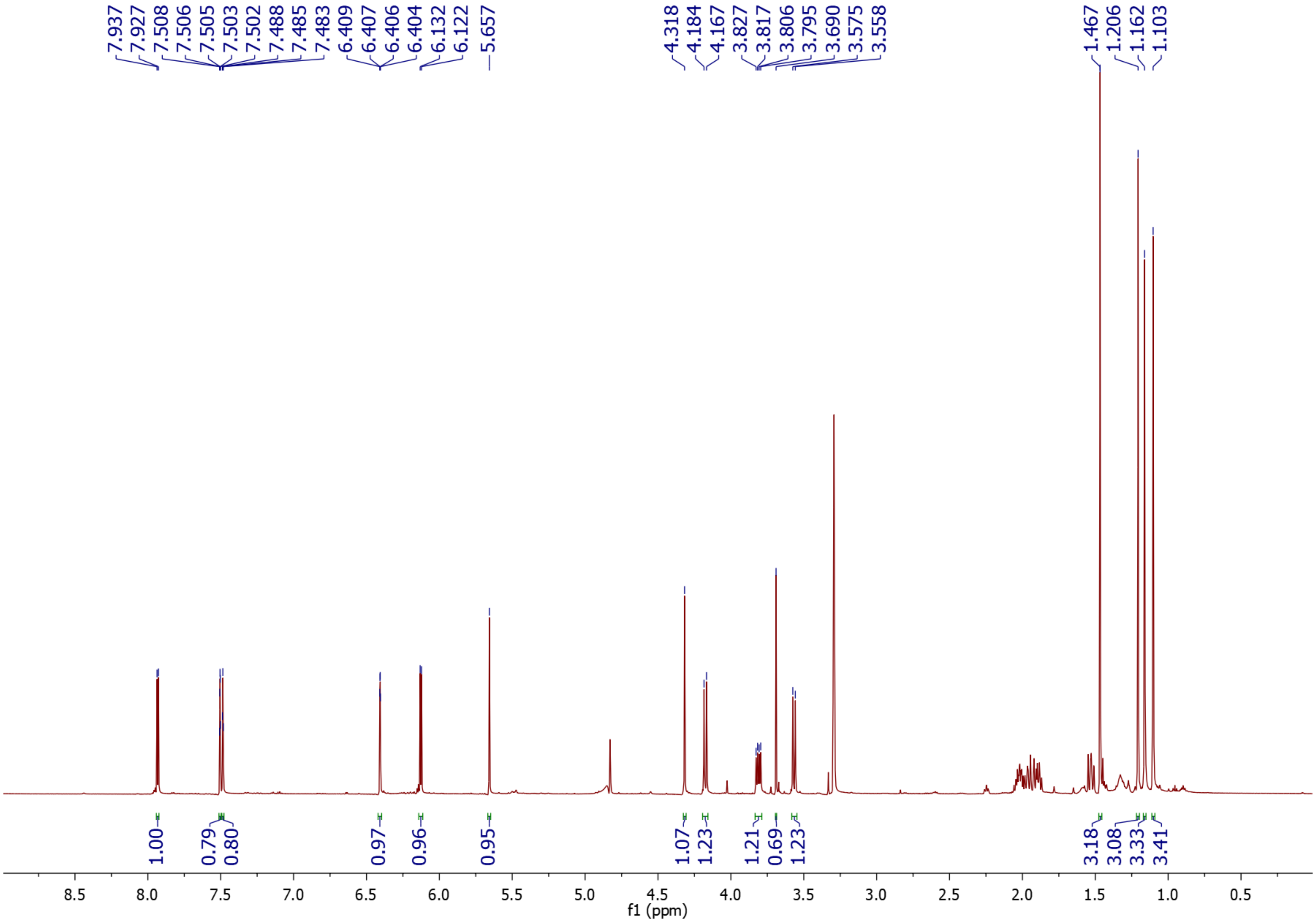

Figure S 40. ^{1}H-NMR (600 MHz) spectrum of compound **8** in CD_3OD

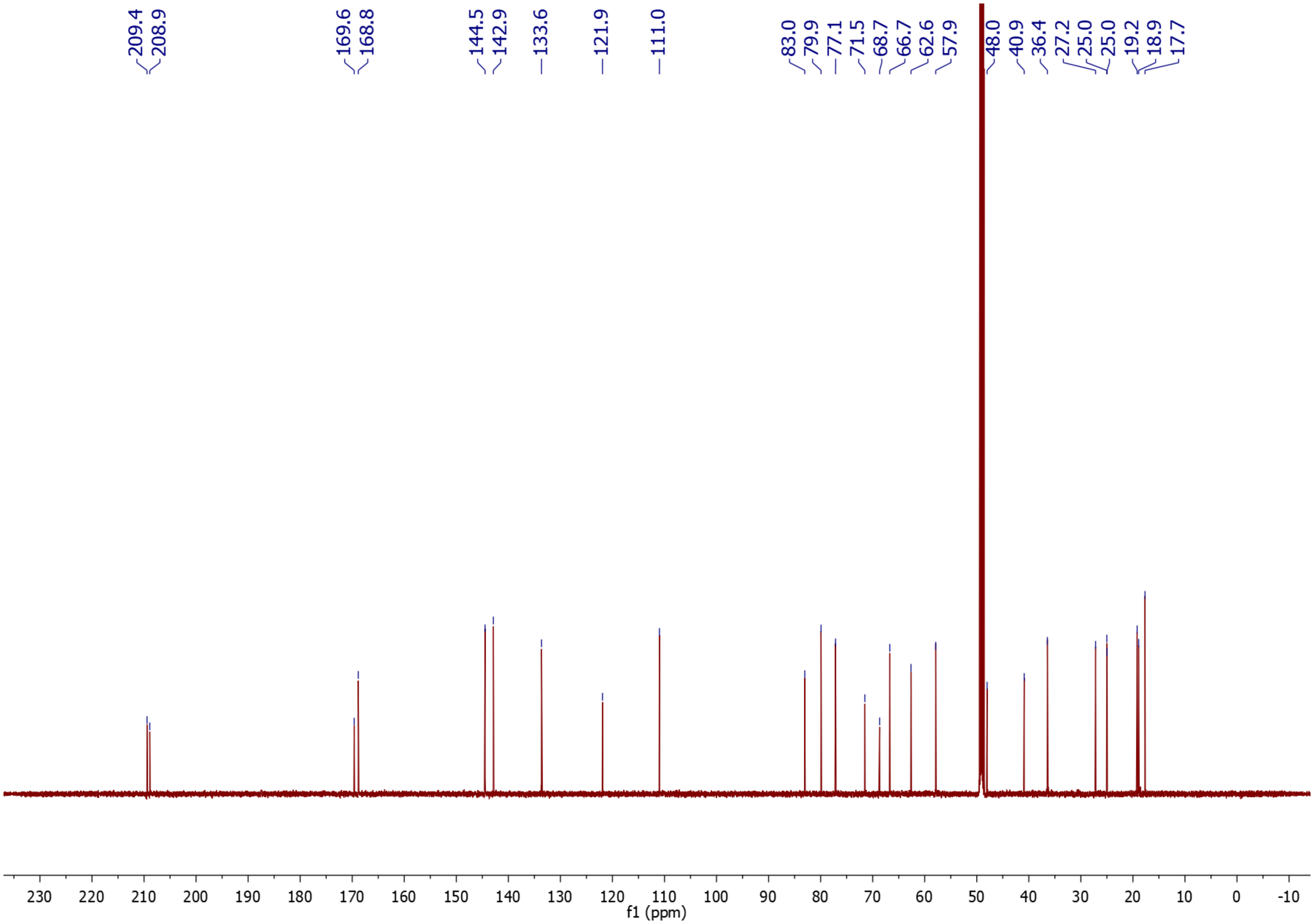

Figure S 41. ^{13}C-NMR (150 MHz) spectrum of compound **8** in CD_3OD

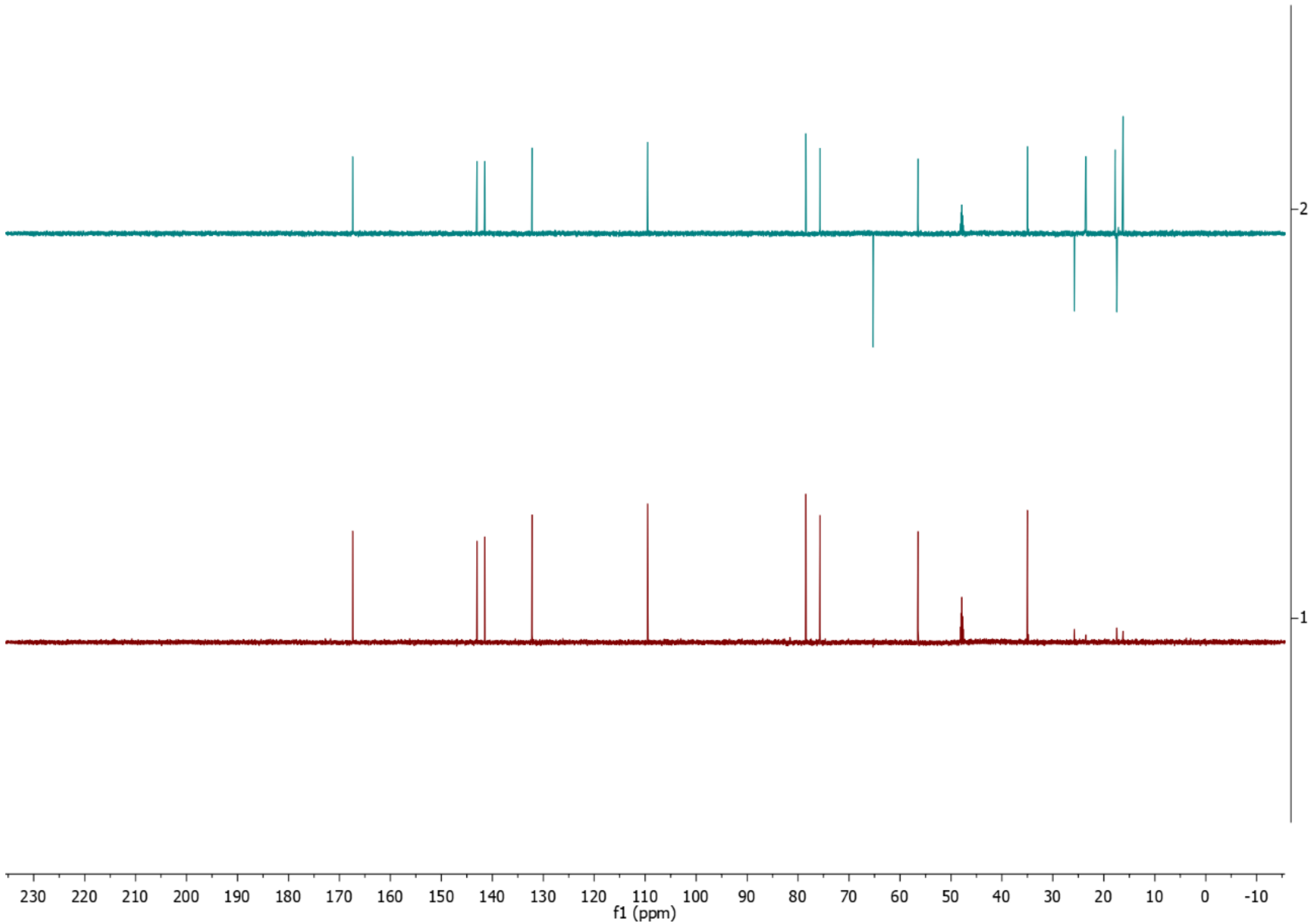

Figure S 42. DEPT-NMR (150 MHz) spectrum of compound **8** in CD_3OD

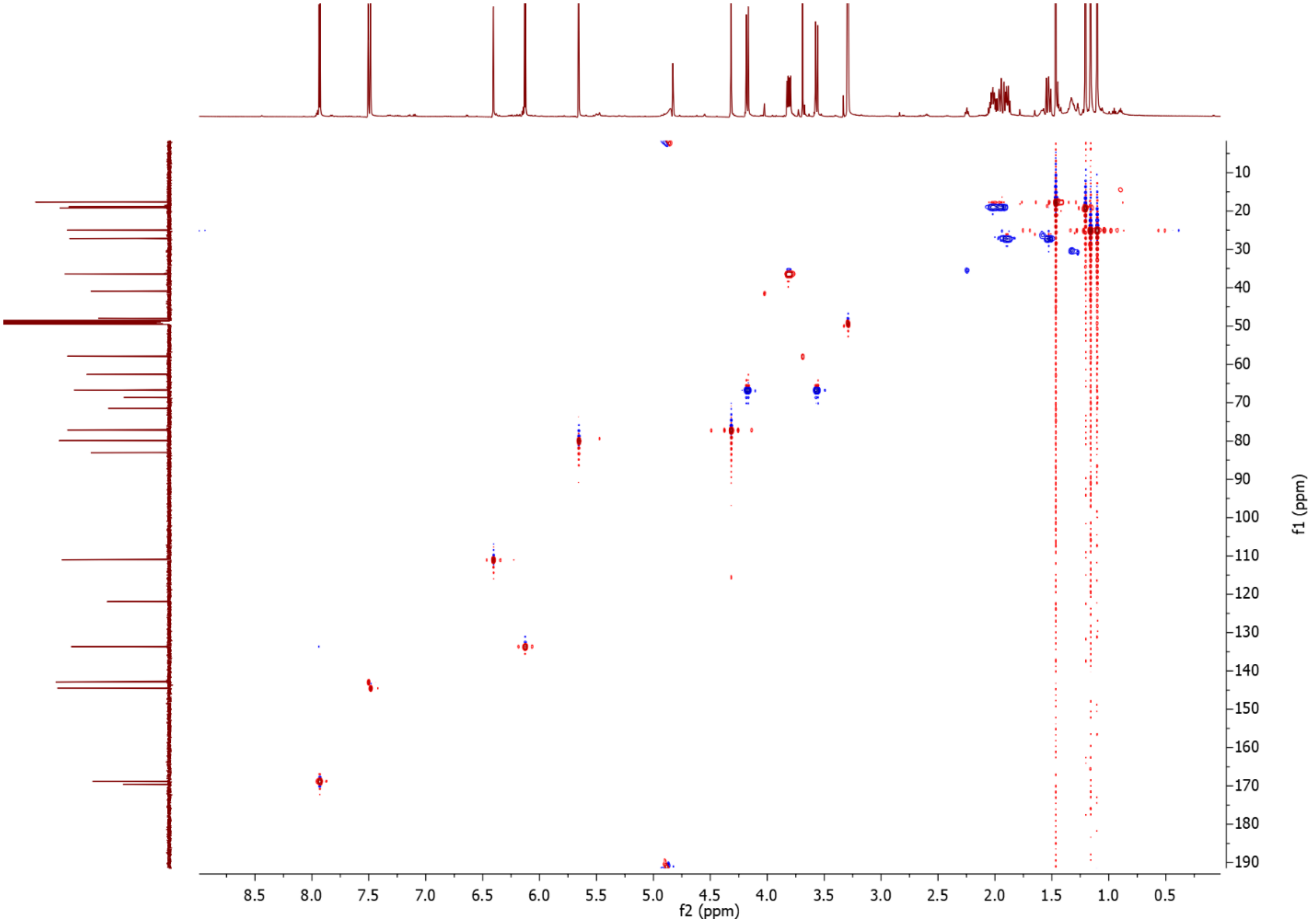

Figure S 43. HSQC spectrum of compound **8** in CD_3OD

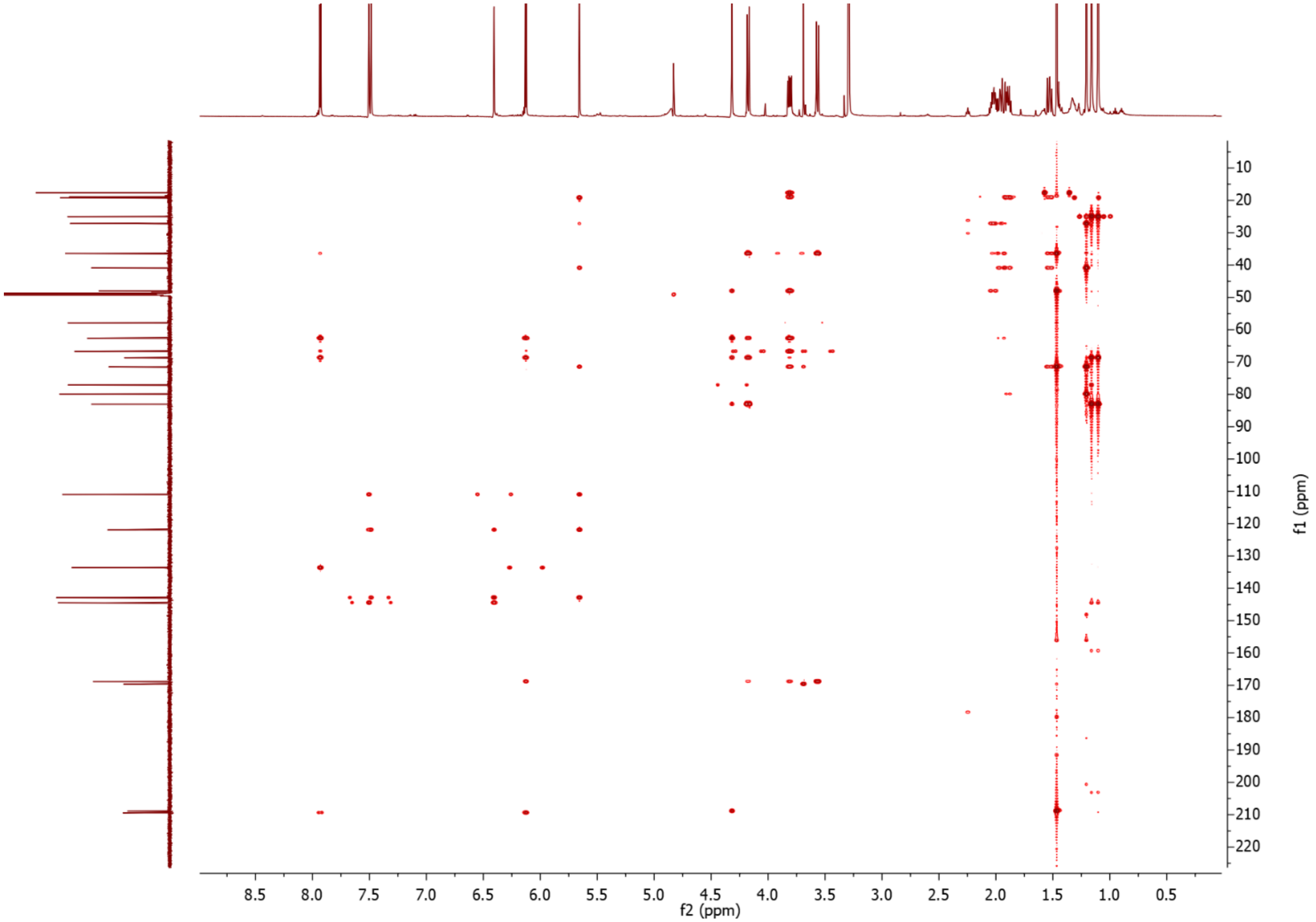

Figure S 44. HMBC spectrum of compound **8** in CD3OD

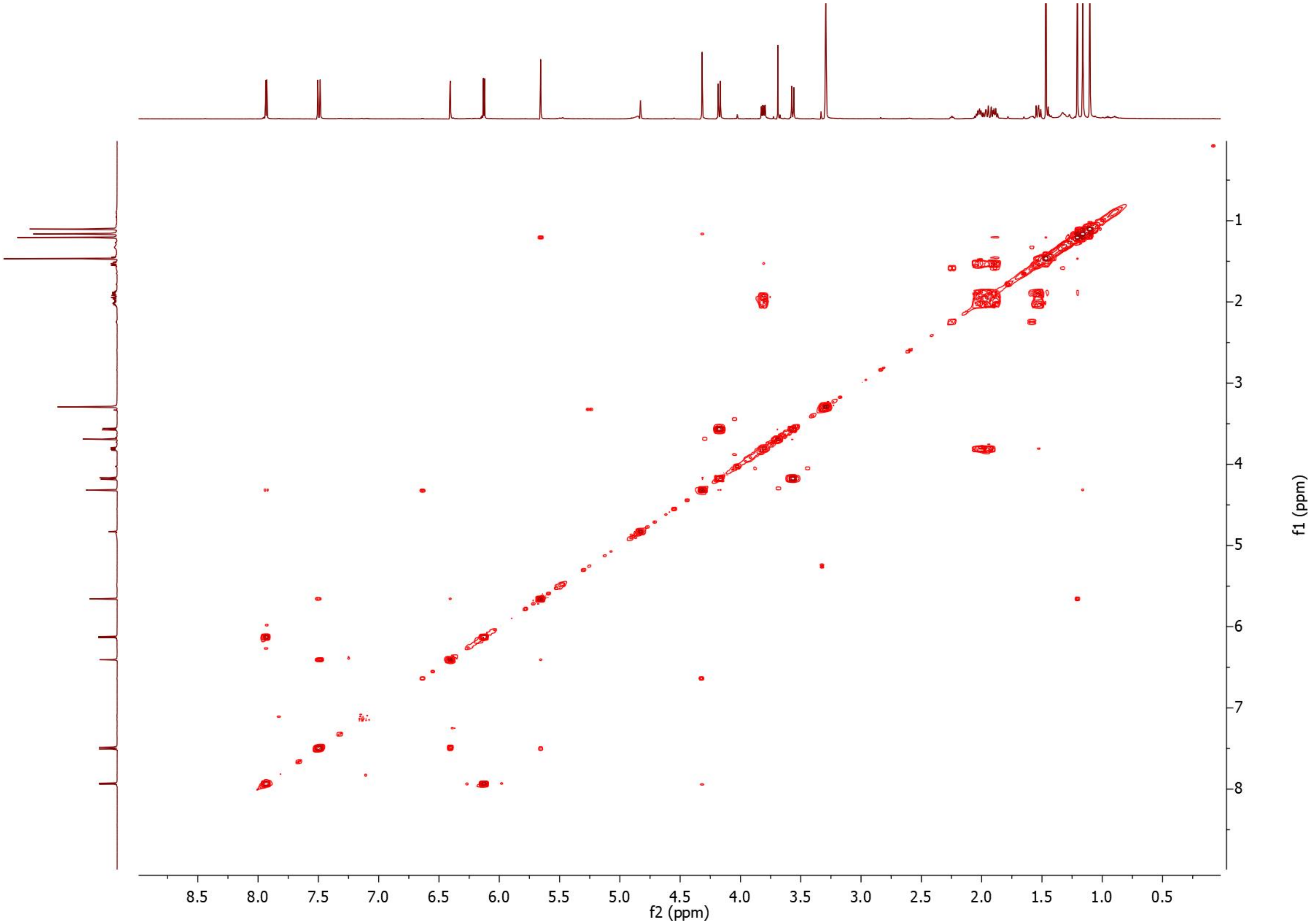

Figure S 45. ^{1}H-^{1}H-COSY spectrum of compound **8** in CD_3OD

Table S 1. Crystal data and experimental details for compound **4**

Crystal data

Empirical formula	C27 H34 O9	
Formula weight	502.54	
Crystal system	Orthorhombic	
Space group	$P2_12_12_1$	
Unit cell dimensions	a = 7.8196(2) Å	α= 90°.
	b = 10.6277(3) Å	β= 90°.
	c = 28.4548(8) Å	γ = 90°.
Volume	2364.72(11) $Å^3$	
Z	4	
F(000)	1072	
Density (calculated)	1.412 Mg/m^3	
Wavelength	1.54178 Å	
Cell parameters reflections used	9189	
Theta range for Cell parameters	3.11 to 72.11°.	
Absorption coefficient	0.877 mm^{-1}	
Temperature	100(2) K	
Crystal size	0.150 x 0.150 x 0.100 mm^3	
Data collection		
Diffractometer	Bruker AXS D8 VENTURE, PhotonIII_C28	
Absorption correction	Semi-empirical from equivalents	
Max. and min. transmission	1.0000 and 0.579373	
No. of measured reflections	24152	
No. of independent reflections	4641 [R(int) = 0.0448]	
No. of observed [I>2_igma(I)]	4610	

Completeness to theta = 67.679°	100.0 %
Theta range for data collection	3.106 to 72.100°.
Refinement	
Final R indices [I>2sigma(I)]	R1 = 0.0310, wR2 = 0.0816
R indices (all data)	R1 = 0.0311, wR2 = 0.0818
Goodness-of-fit on F^2	1.049
No. of reflections	4641
No. of parameters	349
No. of restraints	0
Absolute structure parameter	0.00(5)
Largest diff. peak and hole	0.203 and -0.276 e.$Å^{-3}$

Table S 2. Optical Rotation of compound **3**

3

[Data Information]
Creation Date 2025/4/26 02.35 p.m

[Measurement Information]
Instrument Name P-2000
Model Name P-2000
Serial No. A062461232
Polarizer Dichrom
Faraday Cell Flint Glass

Accessory RSC-200
Accessory S/N B045761260

Light Source Na
Monitor wavelength 589 nm
D.I.T. 5 sec
No. of cycle 5
Cycle interval 2 sec
Temp. Monitor Cell
Temp. Corr. Factor None
Aperture (S) 3.0 nm
Aperture (L) Auto
Mode Specific O.R.
Path Length 50 nm
Cencentration 1.83 w/v%
Water content of sample 0%
Factor 1

No.	Sample Name	Measurement Date	PMT Voltage[V]	Temperature[C]	Optical Rotation Monitor	Specific O.R.
1	3			24.00	0.0101	-49.5848
2	3	2025/4/26 02.35 p.m	312	24.00	0.0140	-49.8785
3	3	2025/4/26 02.35 p.m	399	24.00	0.0071	-49.8245
4	3	2025/4/26 02.35 p.m	389	24.00	0.0137	-49.8970
5	3	2025/4/26 02.35 p.m	356	24.00	0.0138	-49.7787
6	3	2025/4/26 02.35 p.m	382	24.00	0.0116	-49.6752

Table S 3. Optical Rotation of compound **4**

4

[Data Information]
Creation Date 2025/4/26 02.50 p.m

[Measurement Information]
Instrument Name P-2000
Model Name P-2000
Serial No. A062461232
Polarizer Dichrom
Faraday Cell Flint Glass

Accessory RSC-200
Accessory S/N B045761260

Light Source Na
Monitor wavelength 589 nm
D.I.T. 5 sec
No. of cycle 5
Cycle interval 2 sec
Temp. Monitor Cell
Temp. Corr. Factor None
Aperture (S) 3.0 nm
Aperture (L) Auto
Mode Specific O.R.
Path Length 50 nm
Cencentration 1.83 w/v%
Water content of sample 0%
Factor 1

No.	Sample Name	Measurement Date	PMT Voltage[V]	Temperature[C]	Optical Rotation Monitor	Specific O.R.
1	4			24.00	0.0124	-31.4691
2	4	2025/4/26 02.50 p.m	328	24.00	0.0091	-31.1887
3	4	2025/4/26 02.50 p.m	344	24.00	0.0077	-31.6874
4	4	2025/4/26 02.50 p.m	293	24.00	0.0074	-31.5258
5	4	2025/4/26 02.50 p.m	393	24.00	0.0028	-31.1547
6	4	2025/4/26 02.50 p.m	374	24.00	0.0088	-31.6896

Table S 4. Optical Rotation of compound **8**

8

[Data Information]
Creation Date 2025/4/26 03.24 p.m

[Measurement Information]
Instrument Name P-2000
Model Name P-2000
Serial No. A062461232
Polarizer Dichrom
Faraday Cell Flint Glass

Accessory RSC-200
Accessory S/N B045761260

Light Source Na
Monitor wavelength 589 nm
D.I.T. 5 sec
No. of cycle 5
Cycle interval 2 sec
Temp. Monitor Cell
Temp. Corr. Factor None
Aperture (S) 3.0 nm
Aperture (L) Auto
Mode Specific O.R.
Path Length 50 nm
Cencentration 1.83 w/v%
Water content of sample 0%
Factor 1

No.	Sample Name	Measurement Date	PMT Voltage[V]	Temperature[C]	Optical Rotation Monitor	Specific O.R.
1	8			24.00	0.0124	34.9097
2	8	2025/4/26 03.24 p.m	328	24.00	0.0091	34.2643
3	8	2025/4/26 03.24 p.m	344	24.00	0.0077	34.5217
4	8	2025/4/26 03.24 p.m	293	24.00	0.0074	34.6570
5	8	2025/4/26 03.24 p.m	393	24.00	0.0028	34.2718
6	8	2025/4/26 03.24 p.m	374	24.00	0.0088	34.4913

Table S 5. ECD calculation of compound **8**

A panel of relatively favored conformations in an energy range of 3 kcal/mol above the global minimum was obtained by performing the first conformational analysis of compound 8 using the GMMX fucntion searching algorithm via the MMFF94 molecular mechanics force field, obtained conformers were optimized using the Gaussian 16 software package's density functional theory (DFT) using the B3LYP/6-31G(d) level in gas phase. Time-dependent density functional theory (TDDFT) calculations at the M062X/def2TZVP//M062X/def2tzvp level in MeOH using the Polarizable Continuum Model (PCM) solvent model were used to theoretically calculate ECD for these dominating conformers. The Gaussian 16 software program was used to measure each conformer's energies, oscillator strengths, and rotational strengths. The oretical calculations of ECD spectra for each conformer were then approximated by the Gaussian distribution. The final ECD spectrum of the individual conformers was summed up because of Boltzmann-weighed population contribution by the Specdisc software.

Energy analyses of conformers

No.	3D conformers	Free energy			
		Energy (KCal/Mol)	ΔE	Bolt. Factor	Bolt. Distr. (%)
1	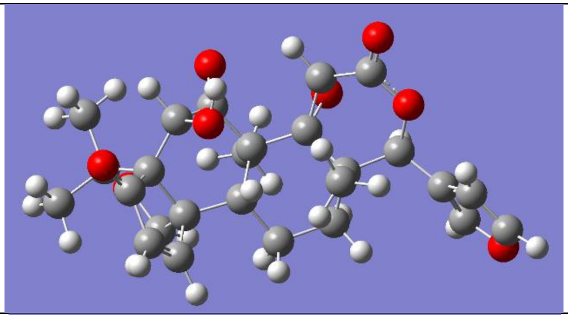	111.818	0.000	1	36.77
2	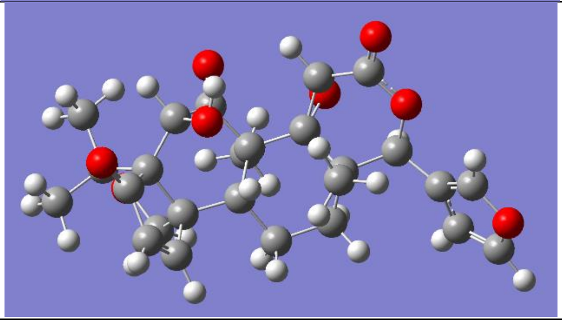	112.149	0.331	0.571779046	21.03

3	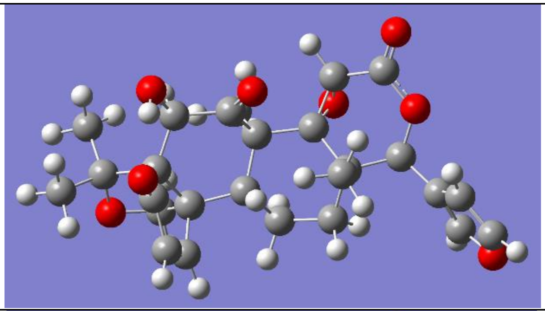	112.328	0.510	0.42261099	15.54
4	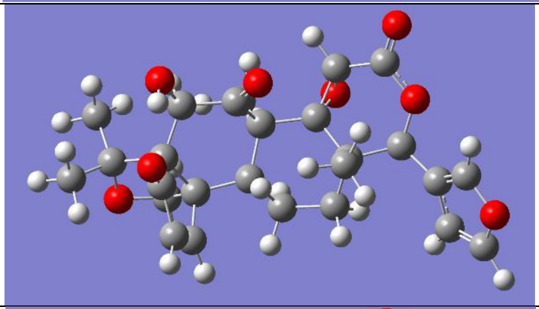	112.451	0.633	0.343341663	12.63
5	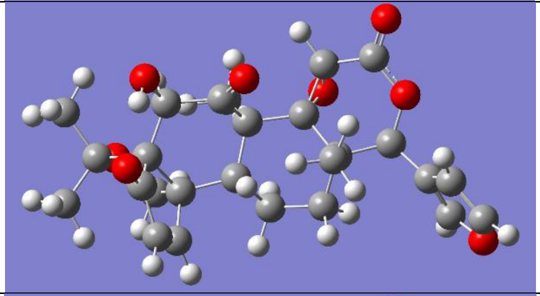	113.168	1.350	0.102292374	3.76
6	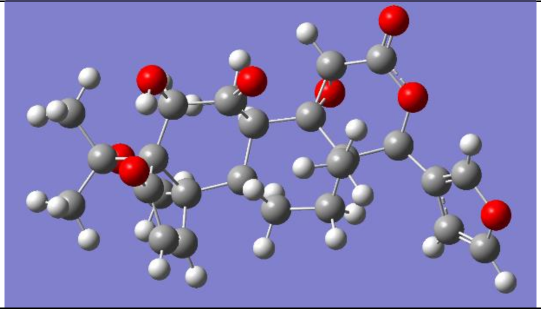	113.273	1.455	0.085670454	3.15

7	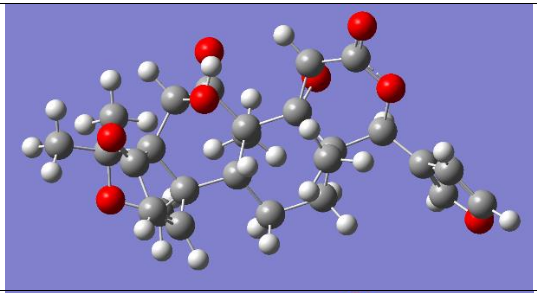	113.558	1.740	0.052941712	1.95
8	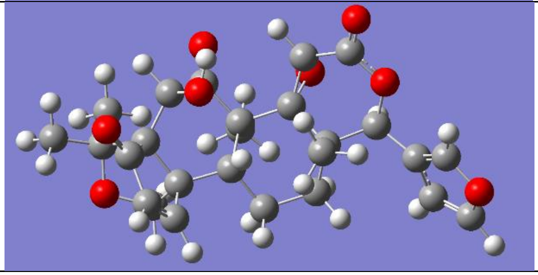	113.695	1.877	0.042006394	1.54

ECD Spectrum

X-Axis: Wavelength (nm)

Y-Axis: Δε (L mol-1 cm-1)

Y-Axis2: Rvel (10-40 esu2 cm2)

Peak information

#	X	Y	Y2
#	320.8000000000	4.4485957400	34.6873391359
#	311.5900000000	4.3189683400	34.6720015718
#	226.5800000000	-7.1585421400	-79.0288055085
#	219.5400000000	9.8911296600	112.6975927409
#	211.9700000000	-8.3565648600	-98.6133682599

#	202.3900000000	-0.6035186200	-7.4590587474
#	199.3300000000	32.9488742200	413.4760112812
#	198.2000000000	-4.6591791200	-58.8014670164
#	192.6600000000	-3.2930251200	-42.7548997124
#	190.5100000000	-6.5689296400	-86.2500265678
#	190.1000000000	5.7645409600	75.8516472955
#	188.0600000000	-5.3366657200	-70.9832651918
#	186.6600000000	2.5821009400	34.6022515236
#	185.0700000000	4.7877932600	64.7115449475
#	182.6200000000	5.3197897000	72.8666041374
#	181.2100000000	-6.8989030000	-95.2314249523
#	177.5600000000	14.5785403000	205.3767654686
#	176.0700000000	-0.5978234000	-8.4931726069
#	175.4700000000	12.6032772800	179.6648087421
#	172.9100000000	-4.6398922400	-67.1228190581
#	171.6000000000	-10.0771502200	-146.8935929201
#	169.6700000000	8.1779865200	120.5656897074
#	167.5300000000	1.0201082400	15.2312684123
#	167.1400000000	2.5252885000	37.7931427215
#	165.9100000000	11.3890424000	171.7105782907
#	165.2800000000	-3.6442769400	-55.1535502287
#	164.6600000000	-15.2678016800	-231.9374101201
#	164.5000000000	-13.1731486800	-200.3115980388

#	163.6500000000	0.1601649600	2.4481267783
#	162.2100000000	6.1456664800	94.7706300459
#	320.8500000000	2.9081802000	22.6726181657
#	311.6200000000	2.6499305600	21.2711796140
#	226.9100000000	-4.5820439600	-50.5112388290
#	219.5500000000	6.0442843600	68.8642546005
#	212.3700000000	-5.6457182800	-66.4979780870
#	201.4400000000	2.0413821200	25.3490097356
#	198.2700000000	6.5930128800	83.1781624813
#	196.4900000000	-4.5374525200	-57.7635725228
#	195.1500000000	-2.6255781200	-33.6541629611
#	190.6000000000	-2.7492657600	-36.0808060681
#	190.1800000000	2.1547642800	28.3411408780
#	187.9400000000	-3.4251389600	-45.5870382783
#	186.4800000000	3.4060377200	45.6877317344
#	184.8300000000	3.5281281200	47.7479014188
#	182.2800000000	1.7873772000	24.5278565860
#	181.1400000000	-3.9471738800	-54.5072534756
#	177.1900000000	12.9336618400	182.5848327675
#	176.0500000000	3.4354444400	48.8123036507
#	175.1100000000	0.6700312400	9.5712025792
#	172.8300000000	-1.7156764400	-24.8312545478
#	171.6000000000	-5.9649912400	-86.9510700795

#	169.3900000000	1.8054282000	26.6609031988
#	168.5100000000	1.2363294000	18.3523178671
#	167.5300000000	5.2974105600	79.0958047055
#	166.4900000000	10.9132188800	163.9634760188
#	165.9200000000	-1.1631845600	-17.5360709572
#	165.4700000000	-6.6772508800	-100.9394261726
#	163.9000000000	-5.5678692400	-84.9752553385
#	163.7500000000	-0.8380040000	-12.8010969680
#	162.0500000000	17.9396964400	276.9162610170
#	306.6000000000	-0.8705190000	-7.1021282150
#	288.9800000000	-1.1734222500	-10.1570814842
#	224.6300000000	-1.6753035000	-18.6555536826
#	217.8500000000	0.4200885000	4.8235372078
#	214.7400000000	8.5240935000	99.2927777547
#	207.9400000000	-1.1522745000	-13.8611835900
#	202.9800000000	0.0918937500	1.1324398167
#	196.1700000000	3.3674842500	42.9393420428
#	193.4800000000	-1.3599105000	-17.5815294594
#	187.9100000000	-1.0621845000	-14.1394485353
#	179.8000000000	-4.5193200000	-62.8732355777
#	178.9800000000	-1.1642182500	-16.2709272599
#	178.4600000000	1.0180852500	14.2700554666
#	178.2100000000	0.3441067500	4.8299599102

#	177.9300000000	1.8784642500	26.4080394344
#	175.3700000000	-0.4381942500	-6.2501980184
#	174.0700000000	1.0783402500	15.4958102968
#	172.1300000000	-0.9568650000	-13.9051764744
#	169.6700000000	0.4415385000	6.5094743865
#	169.3700000000	-0.0605475000	-0.8942153749
#	167.2400000000	-0.6084487500	-9.1005208445
#	166.8600000000	-2.2822020000	-34.2124555976
#	166.5100000000	-0.3573960000	-5.3689798140
#	165.5900000000	3.8543505000	58.2236601731
#	165.0600000000	-4.6185945000	-69.9923213826
#	164.5100000000	-0.5711550000	-8.6844853138
#	164.1000000000	0.7965652500	12.1421384286
#	162.6400000000	-2.2367670000	-34.4013737454
#	158.6700000000	-1.6102807500	-25.3857023661
#	158.4500000000	-2.4230602500	-38.2520199830
#	306.6200000000	-0.4187183000	-3.4158899233
#	289.0300000000	-0.5738982000	-4.9667733721
#	224.6100000000	-0.9242691000	-10.2932317413
#	218.0400000000	0.0591495000	0.6785741461
#	214.7800000000	4.2132022000	49.0682838190
#	207.9900000000	-0.4709118000	-5.6634298110
#	204.2000000000	-0.4333071000	-5.3078968185

#	197.3200000000	-0.2673971000	-3.3897525062
#	193.5700000000	-0.6927659000	-8.9522222717
#	191.3000000000	-0.8466580000	-11.0707093945
#	179.6700000000	-2.2182120000	-30.8823177390
#	179.4700000000	0.0415104000	0.5785587417
#	178.7800000000	-0.6505740000	-9.1024892413
#	178.5200000000	0.7824983000	10.9642499857
#	177.6500000000	0.4582829000	6.4528392335
#	175.5900000000	0.0791386000	1.1273817201
#	174.7300000000	0.4168806000	5.9679697439
#	172.2000000000	-0.3129542000	-4.5460063880
#	169.9900000000	1.2487336000	18.3750626314
#	169.5400000000	-0.2404473000	-3.5475631111
#	168.6200000000	-0.5680138000	-8.4262086034
#	167.3200000000	1.1958962000	17.8783746706
#	166.6700000000	-2.5945269000	-38.9388522991
#	165.8900000000	0.4102113000	6.1854288673
#	165.4000000000	3.3672163000	50.9234611823
#	164.5800000000	0.3498539000	5.3173110468
#	164.3000000000	-1.7165528000	-26.1337665652
#	164.1700000000	1.2184327000	18.5647947542
#	158.7900000000	1.8954348000	29.8585082051
#	158.5300000000	-1.7810697000	-28.1029467288

#	310.6600000000	-0.3072969900	-2.4743171926
#	287.5400000000	-1.4581967400	-12.6852858767
#	224.9300000000	10.8427047300	120.5792705667
#	224.4500000000	-6.4500579000	-71.8830354128
#	221.6300000000	1.6215685200	18.3016043591
#	206.6600000000	0.2313639900	2.8004108051
#	200.5700000000	-2.2208858100	-27.6976331709
#	198.3100000000	0.4680947700	5.9043429962
#	196.1800000000	4.5770739300	58.3600427712
#	190.1500000000	-1.5200885700	-19.9965451505
#	187.8800000000	-1.0380813300	-13.8208016166
#	181.7900000000	-0.1563318900	-2.1510969928
#	179.3600000000	-0.3092661000	-4.3130960215
#	178.1000000000	1.6068838500	22.5685143361
#	175.4500000000	-0.9557133300	-13.6256326253
#	174.9800000000	-1.0635896700	-15.2043585582
#	173.8900000000	0.3585839400	5.1582053992
#	172.3600000000	-2.0093802300	-29.1613781046
#	170.2000000000	1.3113629100	19.2728412773
#	170.0200000000	0.3239636400	4.7662702401
#	169.3200000000	0.3223162800	4.7616381228
#	167.1500000000	5.1079871700	76.4409051938
#	166.3000000000	-1.4240783700	-21.4202258323

#	165.3200000000	-0.1221491700	-1.8481938812
#	165.1800000000	-4.2652595700	-64.5907620884
#	164.6000000000	-1.4142971700	-21.4928122964
#	162.6000000000	0.0497425500	0.7652263360
#	162.4500000000	1.3173989400	20.2852329029
#	162.0600000000	2.1663684900	33.4378927160
#	160.2300000000	0.5283778500	8.2486556735
#	310.7100000000	-0.2177069400	-1.7526671508
#	287.5900000000	-1.0302811200	-8.9611627516
#	224.9200000000	6.7864797000	75.4742635287
#	224.4200000000	-4.3660960200	-48.6647087931
#	221.8300000000	1.6160958000	18.2233923981
#	207.9500000000	-0.6288678000	-7.5645454703
#	200.4900000000	-1.2567733200	-15.6800210384
#	198.2800000000	1.1215195200	14.1484977810
#	197.3700000000	-1.7361847800	-22.0037756378
#	191.2800000000	-1.5825229200	-20.6948770229
#	190.2300000000	-0.8868924900	-11.6620358971
#	181.7500000000	-0.2496671100	-3.4361278857
#	179.8000000000	0.0674041500	0.9377333320
#	177.7300000000	0.8289151200	11.6662619865
#	176.2900000000	-1.0212285600	-14.4903100617
#	175.5700000000	-0.2753368200	-3.9228019949

#	173.9400000000	-0.0779001300	-1.1202659116
#	172.4500000000	-1.0472101200	-15.1898342943
#	172.0500000000	1.0132630200	14.7316000757
#	170.1300000000	0.2952684900	4.3412880768
#	169.2600000000	0.0314523000	0.4648153667
#	167.6700000000	2.8024444800	41.8084490952
#	166.3200000000	-1.8397457100	-27.6691442152
#	165.8500000000	6.1709858100	93.0725207340
#	165.2100000000	1.3164257700	19.9316131094
#	164.5900000000	-2.5737069600	-39.1145245984
#	164.2000000000	0.5705518500	8.6916928267
#	162.6400000000	3.0444757200	46.8238967683
#	161.9600000000	1.5401826000	23.7873930676
#	160.1600000000	0.3006679500	4.6958636782
#	318.0100000000	0.2150010000	1.6911499124
#	310.2000000000	0.2523231000	2.0346870576
#	226.6200000000	-0.5574096000	-6.1525990384
#	215.9600000000	1.4763249000	17.0997999770
#	207.4800000000	-0.4958928000	-5.9785238729
#	200.9100000000	0.8274069000	10.3014886656
#	200.1000000000	0.2412639000	3.0159744608
#	198.8500000000	1.1490768000	14.4545929222
#	192.7300000000	-0.5049054000	-6.5530442190

#	189.0000000000	-1.0354662000	-13.7042895910
#	187.0000000000	0.6906627000	9.2386135274
#	186.2300000000	0.0050598000	0.0679619939
#	185.0100000000	0.0545049000	0.7369240809
#	184.3400000000	-0.2572668000	-3.4909736105
#	179.0500000000	2.0607129000	28.7889329892
#	177.8700000000	0.2937465000	4.1309734144
#	176.5600000000	-0.2406861000	-3.4098956907
#	175.6400000000	-0.0333369000	-0.4747710052
#	174.0400000000	-0.8836884000	-12.7008411380
#	172.1400000000	-1.0070541000	-14.6336750728
#	170.7000000000	-0.1250775000	-1.8328548600
#	169.3600000000	0.4199283000	6.2022133746
#	167.4700000000	2.8719414000	42.8964110517
#	167.2500000000	-0.2220507000	-3.3209965509
#	166.4300000000	-0.1888029000	-2.8376540776
#	165.6000000000	-2.2215465000	-33.5565636004
#	164.9800000000	0.5326857000	8.0764803986
#	164.4400000000	-1.6739514000	-25.4634814312
#	163.2900000000	0.0586872000	0.8990134826
#	161.2000000000	1.4443110000	22.4118695424
#	318.0900000000	0.3607485000	2.8368535886
#	310.2000000000	0.3713267500	2.9943105977

#	226.8700000000	-0.9008470000	-9.9324495987
#	215.9500000000	2.2818165000	26.4307758878
#	207.8900000000	-1.0262560000	-12.3482244347
#	200.9100000000	1.8361147500	22.8602339258
#	198.8200000000	-0.1639905000	-2.0631985549
#	196.4300000000	-0.3871070000	-4.9295311174
#	195.2000000000	-0.9751242500	-12.4957563348
#	189.2800000000	-1.4091130000	-18.6218789269
#	186.8800000000	0.1097817500	1.4694327956
#	186.2300000000	0.7878097500	10.5816675391
#	184.5100000000	-0.9523655000	-12.9111863663
#	183.9900000000	1.1070655000	15.0508664389
#	178.9900000000	3.3612570000	46.9737634056
#	177.9900000000	0.1868427500	2.6258085134
#	176.2800000000	-0.3403315000	-4.8292702596
#	175.1500000000	0.0524237500	0.7486870938
#	173.8900000000	-1.7822290000	-25.6372420093
#	172.2400000000	-1.5154692500	-22.0087570427
#	170.6300000000	-0.5910050000	-8.6639945159
#	169.2100000000	0.7679027500	11.3517443472
#	167.6400000000	1.4003707500	20.8952601792
#	167.4500000000	-0.3346577500	-4.9991726923
#	167.1600000000	3.6028227500	53.9129304132

#	166.1300000000	1.7582165000	26.4732152410
#	165.4000000000	-3.0364252500	-45.9208050731
#	164.6700000000	-0.4107327500	-6.2391762167
#	163.9400000000	-1.0806857500	-16.4890979751
#	160.8900000000	4.4484792500	69.1615824925

Spectra

#	X	Y	DY/DX
	165.9500000000	10.1575568734	0.1927418793
	166.4260060000	10.3831286525	0.2169900559
	166.9020120000	10.6156793038	0.2315558914
	167.3780180000	10.8561254680	0.2397037629
	167.8540240000	11.1049038463	0.2464184551
	168.3300300000	11.3619349779	0.2575739637
	168.8060360000	11.6265994836	0.2789969086
	169.2820420000	11.8977275887	0.3155508971
	169.7580480000	12.1736026254	0.3703582709
	170.2340540000	12.4519790247	0.4442514148
	170.7100600000	12.7301150467	0.5355116256
	171.1860660000	13.0048201478	0.6399147610
	171.6620720000	13.2725164673	0.7510648847
	172.1380780000	13.5293134556	0.8609645966
	172.6140840000	13.7710941787	0.9607471661

173.0900900000	13.9936113586	1.0414829346
173.5660960000	14.1925907782	1.0949710980
174.0421020000	14.3638393130	1.1144368585
174.5181080000	14.5033545874	1.0950708932
174.9941140000	14.6074330990	1.0343702551
175.4701200000	14.6727736286	0.9322640814
175.9461260000	14.6965728480	0.7910308751
176.4221320000	14.6766102668	0.6150341559
176.8981380000	14.6113199837	0.4103181818
177.3741440000	14.4998471384	0.1841142493
177.8501500000	14.3420874527	-0.0556893631
178.3261560000	14.1387087910	-0.3010638849
178.8021620000	13.8911542352	-0.5443008009
179.2781680000	13.6016267305	-0.7783855992
179.7541740000	13.2730558952	-0.9972657769
180.2301800000	12.9090480837	-1.1960069722
180.7061860000	12.5138212275	-1.3708424946
181.1821920000	12.0921263498	-1.5191307434
181.6581980000	11.6491579375	-1.6392414367
182.1342040000	11.1904555712	-1.7303949917
182.6102100000	10.7217993430	-1.7924799081
183.0862160000	10.2491016532	-1.8258710135
183.5622220000	9.7782979566	-1.8312674974

184.0382280000	9.3152389517	-1.8095644726
184.5142340000	8.8655865696	-1.7617660546
184.9902400000	8.4347159282	-1.6889422704
185.4662460000	8.0276251947	-1.5922270317
185.9422520000	7.6488550380	-1.4728503160
186.4182580000	7.3024190679	-1.3321948243
186.8942640000	6.9917463638	-1.1718658089
187.3702700000	6.7196368819	-0.9937624436
187.8462760000	6.4882302259	-0.8001398911
188.3222820000	6.2989879569	-0.5936528848
188.7982880000	6.1526893258	-0.3773739305
189.2742940000	6.0494400314	-0.1547818736
189.7503000000	5.9886933468	0.0702806839
190.2263060000	5.9692827260	0.2936800180
190.7023120000	5.9894647904	0.5110905600
191.1783180000	6.0469714260	0.7181155318
191.6543240000	6.1390695774	0.9104222910
192.1303300000	6.2626272203	1.0838849254
192.6063360000	6.4141839277	1.2347263595
193.0823420000	6.5900244113	1.3596524646
193.5583480000	6.7862534263	1.4559713247
194.0343540000	6.9988704704	1.5216918227
194.5103600000	7.2238427776	1.5555969893

194.9863660000	7.4571752163	1.5572889922
195.4623720000	7.6949758281	1.5272041548
195.9383780000	7.9335159009	1.4665978855
196.4143840000	8.1692836390	1.3775008042
196.8903900000	8.3990306792	1.2626485902
197.3663960000	8.6198108958	1.1253891235
197.8424020000	8.8290111307	0.9695712821
198.3184080000	9.0243736850	0.7994203059
198.7944140000	9.2040105903	0.6194049093
199.2704200000	9.3664098610	0.4341013559
199.7464260000	9.5104340886	0.2480594996
200.2224320000	9.6353118856	0.0656753913
200.6984380000	9.7406228124	-0.1089255160
201.1744440000	9.8262765225	-0.2719912212
201.6504500000	9.8924869413	-0.4202295104
202.1264560000	9.9397423507	-0.5508759934
202.6024620000	9.9687722830	-0.6617428595
203.0784680000	9.9805121358	-0.7512481216
203.5544740000	9.9760664109	-0.8184258108
204.0304800000	9.9566714425	-0.8629181769
204.5064860000	9.9236584364	-0.8849514546
204.9824920000	9.8784175702	-0.8852971349
205.4584980000	9.8223638339	-0.8652209637

205.9345040000	9.7569051958	-0.8264220555
206.4105100000	9.6834135903	-0.7709645844
206.8865160000	9.6031991224	-0.7012044978
207.3625220000	9.5174877846	-0.6197136113
207.8385280000	9.4274028835	-0.5292032940
208.3145340000	9.3339502776	-0.4324497583
208.7905400000	9.2380074379	-0.3322227440
209.2665460000	9.1403162576	-0.2312191337
209.7425520000	9.0414794663	-0.1320027777
210.2185580000	8.9419604334	-0.0369515439
210.6945640000	8.8420860941	0.0517876550
211.1705700000	8.7420526837	0.1323353535
211.6465760000	8.6419339334	0.2031072823
212.1225820000	8.5416913548	0.2628333346
212.5985880000	8.4411862287	0.3105679144
213.0745940000	8.3401929087	0.3456920197
213.5506000000	8.2384130534	0.3679075728
214.0266060000	8.1354904164	0.3772246585
214.5026120000	8.0310258389	0.3739424418
214.9786180000	7.9245921193	0.3586246377
215.4546240000	7.8157484587	0.3320704686
215.9306300000	7.7040542203	0.2952820958
216.4066360000	7.5890817736	0.2494295242

216.8826420000	7.4704282316	0.1958139809
217.3586480000	7.3477259305	0.1358307344
217.8346540000	7.2206515363	0.0709322760
218.3106600000	7.0889337009	0.0025927135
218.7866660000	6.9523592266	-0.0677258610
219.2626720000	6.8107777281	-0.1386043629
219.7386780000	6.6641048145	-0.2086945178
220.2146840000	6.5123238365	-0.2767430389
220.6906900000	6.3554862728	-0.3416118702
221.1666960000	6.1937108445	-0.4022943045
221.6427020000	6.0271814655	-0.4579269069
222.1187080000	5.8561441499	-0.5077972839
222.5947140000	5.6809030043	-0.5513478437
223.0707200000	5.5018154412	-0.5881757815
223.5467260000	5.3192867511	-0.6180296028
224.0227320000	5.1337641714	-0.6408025588
224.4987380000	4.9457305872	-0.6565234173
224.9747440000	4.7556979937	-0.6653450209
225.4507500000	4.5642008452	-0.6675311046
225.9267560000	4.3717894036	-0.6634418420
226.4027620000	4.1790231927	-0.6535185833
226.8787680000	3.9864646530	-0.6382682205
227.3547740000	3.7946730787	-0.6182475863

227.8307800000	3.6041989096	-0.5940482497
228.3067860000	3.4155784357	-0.5662820269
228.7827920000	3.2293289640	-0.5355674738
229.2587980000	3.0459444811	-0.5025175751
229.7348040000	2.8658918388	-0.4677287907
230.2108100000	2.6896074766	-0.4317715702
230.6868160000	2.5174946862	-0.3951823965
231.1628220000	2.3499214143	-0.3584573731
231.6388280000	2.1872185934	-0.3220473324
232.1148340000	2.0296789805	-0.2863544049
232.5908400000	1.8775564825	-0.2517299602
233.0668460000	1.7310659357	-0.2184738072
233.5428520000	1.5903833100	-0.1868345256
234.0188580000	1.4556462995	-0.1570107843
234.4948640000	1.3269552622	-0.1291535010
234.9708700000	1.2043744698	-0.1033686899
235.4468760000	1.0879336263	-0.0797208520
235.9228820000	0.9776296170	-0.0582367644
236.3988880000	0.8734284468	-0.0389095353
236.8748940000	0.7752673313	-0.0217028036
237.3509000000	0.6830569029	-0.0065549730
237.8269060000	0.5966834981	0.0066166151
238.3029120000	0.5160114932	0.0179116492

238.7789180000	0.4408856597	0.0274430247
239.2549240000	0.3711335108	0.0353332803
239.7309300000	0.3065676163	0.0417113342
240.2069360000	0.2469878630	0.0467095458
240.6829420000	0.1921836438	0.0504611180
241.1589480000	0.1419359573	0.0530978454
241.6349540000	0.0960194070	0.0547482046
242.1109600000	0.0542040884	0.0555357754
242.5869660000	0.0162573553	0.0555779772
243.0629720000	-0.0180545386	0.0549850979
243.5389780000	-0.0489649287	0.0538595920
244.0149840000	-0.0767053601	0.0522956202
244.4909900000	-0.1015043386	0.0503788032
244.9669960000	-0.1235862137	0.0481861611
245.4430020000	-0.1431701874	0.0457862103
245.9190080000	-0.1604694473	0.0432391921
246.3950140000	-0.1756904184	0.0405974061
246.8710200000	-0.1890321277	0.0379056273
247.3470260000	-0.2006856754	0.0352015832
247.8230320000	-0.2108338060	0.0325164731
248.2990380000	-0.2196505729	0.0298755136
248.7750440000	-0.2273010878	0.0272984945
249.2510500000	-0.2339413487	0.0248003339

249.7270560000	-0.2397181390	0.0223916226
250.2030620000	-0.2447689903	0.0200791496
250.6790680000	-0.2492222014	0.0178664031
251.1550740000	-0.2531969082	0.0157540419
251.6310800000	-0.2568031960	0.0137403351
252.1070860000	-0.2601422504	0.0118215675
252.5830920000	-0.2633065390	0.0099924112
253.0590980000	-0.2663800205	0.0082462621
253.5351040000	-0.2694383741	0.0065755442
254.0111100000	-0.2725492478	0.0049719807
254.4871160000	-0.2757725183	0.0034268370
254.9631220000	-0.2791605622	0.0019311336
255.4391280000	-0.2827585320	0.0004758357
255.9151340000	-0.2866046375	-0.0009479819
256.3911400000	-0.2907304281	-0.0023489905
256.8671460000	-0.2951610741	-0.0037354811
257.3431520000	-0.2999156473	-0.0051152557
257.8191580000	-0.3050073965	-0.0064955327
258.2951640000	-0.3104440202	-0.0078828647
258.7711700000	-0.3162279332	-0.0092830688
259.2471760000	-0.3223565278	-0.0107011664
259.7231820000	-0.3288224289	-0.0121413318
260.1991880000	-0.3356137434	-0.0136068498

260.6751940000	-0.3427143028	-0.0151000797
261.1512000000	-0.3501039008	-0.0166224278
261.6272060000	-0.3577585245	-0.0181743250
262.1032120000	-0.3656505807	-0.0197552122
262.5792180000	-0.3737491167	-0.0213635316
263.0552240000	-0.3820200381	-0.0229967242
263.5312300000	-0.3904263207	-0.0246512343
264.0072360000	-0.3989282205	-0.0263225208
264.4832420000	-0.4074834799	-0.0280050746
264.9592480000	-0.4160475321	-0.0296924434
265.4352540000	-0.4245737023	-0.0313772641
265.9112600000	-0.4330134089	-0.0330513009
266.3872660000	-0.4413163615	-0.0347054922
266.8632720000	-0.4494307599	-0.0363300036
267.3392780000	-0.4573034909	-0.0379142885
267.8152840000	-0.4648803253	-0.0394471551
268.2912900000	-0.4721061142	-0.0409168402
268.7672960000	-0.4789249856	-0.0423110896
269.2433020000	-0.4852805395	-0.0436172435
269.7193080000	-0.4911160443	-0.0448223277
270.1953140000	-0.4963746311	-0.0459131492
270.6713200000	-0.5009994885	-0.0468763957
271.1473260000	-0.5049340560	-0.0476987382

271.6233320000	-0.5081222161	-0.0483669361
272.0993380000	-0.5105084855	-0.0488679436
272.5753440000	-0.5120382037	-0.0491890166
273.0513500000	-0.5126577200	-0.0493178194
273.5273560000	-0.5123145769	-0.0492425301
274.0033620000	-0.5109576911	-0.0489519433
274.4793680000	-0.5085375305	-0.0484355707
274.9553740000	-0.5050062867	-0.0476837365
275.4313800000	-0.5003180428	-0.0466876686
275.9073860000	-0.4944289362	-0.0454395839
276.3833920000	-0.4872973153	-0.0439327673
276.8593980000	-0.4788838896	-0.0421616437
277.3354040000	-0.4691518734	-0.0401218419
277.8114100000	-0.4580671218	-0.0378102509
278.2874160000	-0.4455982582	-0.0352250668
278.7634220000	-0.4317167948	-0.0323658319
279.2394280000	-0.4163972430	-0.0292334633
279.7154340000	-0.3996172147	-0.0258302728
280.1914400000	-0.3813575150	-0.0221599777
280.6674460000	-0.3616022237	-0.0182277010
281.1434520000	-0.3403387669	-0.0140399638
281.6194580000	-0.3175579788	-0.0096046668
282.0954640000	-0.2932541515	-0.0049310648

282.5714700000	-0.2674250749	-0.0000297307
283.0474760000	-0.2400720653	0.0050874873
283.5234820000	-0.2111999822	0.0104075168
283.9994880000	-0.1808172348	0.0159161196
284.4754940000	-0.1489357774	0.0215979572
284.9515000000	-0.1155710923	0.0274366617
285.4275060000	-0.0807421639	0.0334149129
285.9035120000	-0.0444714397	0.0395145207
286.3795180000	-0.0067847825	0.0457165111
286.8555240000	0.0322885889	0.0520012164
287.3315300000	0.0727161690	0.0583483692
287.8075360000	0.1144622441	0.0647371976
288.2835420000	0.1574879812	0.0711465236
288.7595480000	0.2017515251	0.0775548618
289.2355540000	0.2472081048	0.0839405199
289.7115600000	0.2938101476	0.0902816985
290.1875660000	0.3415074015	0.0965565911
290.6635720000	0.3902470641	0.1027434827
291.1395780000	0.4399739187	0.1088208475
291.6155840000	0.4906304766	0.1147674447
292.0915900000	0.5421571245	0.1205624116
292.5675960000	0.5944922777	0.1261853546
293.0436020000	0.6475725365	0.1316164368

293.5196080000	0.7013328479	0.1368364620
293.9956140000	0.7557066694	0.1418269549
294.4716200000	0.8106261362	0.1465702379
294.9476260000	0.8660222295	0.1510495026
295.4236320000	0.9218249478	0.1552488771
295.8996380000	0.9779634773	0.1591534886
296.3756440000	1.0343663636	0.1627495204
296.8516500000	1.0909616833	0.1660242644
297.3276560000	1.1476772139	0.1689661671
297.8036620000	1.2044406031	0.1715648714
298.2796680000	1.2611795359	0.1738112514
298.7556740000	1.3178218991	0.1756974424
299.2316800000	1.3742959432	0.1772168646
299.7076860000	1.4305304407	0.1783642414
300.1836920000	1.4864548412	0.1791356107
300.6596980000	1.5419994213	0.1795283323
301.1357040000	1.5970954305	0.1795410873
301.6117100000	1.6516752325	0.1791738732
302.0877160000	1.7056724403	0.1784279929
302.5637220000	1.7590220464	0.1773060381
303.0397280000	1.8116605469	0.1758118669
303.5157340000	1.8635260592	0.1739505766
303.9917400000	1.9145584340	0.1717284717

304.4677460000	1.9646993597	0.1691530255
304.9437520000	2.0138924613	0.1662328392
305.4197580000	2.0620833918	0.1629775946
305.8957640000	2.1092199160	0.1593980037
306.3717700000	2.1552519888	0.1555057544
306.8477760000	2.2001318247	0.1513134519
307.3237820000	2.2438139617	0.1468345581
307.7997880000	2.2862553166	0.1420833268
308.2757940000	2.3274152345	0.1370747370
308.7518000000	2.3672555299	0.1318244244
309.2278060000	2.4057405223	0.1263486100
309.7038120000	2.4428370633	0.1206640283
310.1798180000	2.4785145580	0.1147878541
310.6558240000	2.5127449794	0.1087376281
311.1318300000	2.5455028757	0.1025311832
311.6078360000	2.5767653721	0.0961865699
312.0838420000	2.6065121659	0.0897219830
312.5598480000	2.6347255154	0.0831556884
313.0358540000	2.6613902233	0.0765059516
313.5118600000	2.6864936146	0.0697909672
313.9878660000	2.7100255089	0.0630287910
314.4638720000	2.7319781872	0.0562372735
314.9398780000	2.7523463548	0.0494339960

315.4158840000	2.7711270987	0.0426362099
315.8918900000	2.7883198409	0.0358607781
316.3678960000	2.8039262875	0.0291241206
316.8439020000	2.8179503744	0.0224421625
317.3199080000	2.8303982089	0.0158302863
317.7959140000	2.8412780078	0.0093032870
318.2719200000	2.8506000335	0.0028753326
318.7479260000	2.8583765262	-0.0034400733
319.2239320000	2.8646216343	-0.0096301238
319.6999380000	2.8693513422	-0.0156827370
320.1759440000	2.8725833966	-0.0215865807
320.6519500000	2.8743372303	-0.0273310924
321.1279560000	2.8746338855	-0.0329064963
321.6039620000	2.8734959351	-0.0383038148
322.0799680000	2.8709474040	-0.0435148775
322.5559740000	2.8670136880	-0.0485323256
323.0319800000	2.8617214746	-0.0533496129
323.5079860000	2.8550986612	-0.0579610025
323.9839920000	2.8471742747	-0.0623615617
324.4599980000	2.8379783909	-0.0665471513
324.9360040000	2.8275420541	-0.0705144142
325.4120100000	2.8158971972	-0.0742607591
325.8880160000	2.8030765631	-0.0777843426

326.3640220000	2.7891136260	-0.0810840484
326.8400280000	2.7740425147	-0.0841594643
327.3160340000	2.7578979365	-0.0870108567
327.7920400000	2.7407151028	-0.0896391439
328.2680460000	2.7225296561	-0.0920458672
328.7440520000	2.7033775981	-0.0942331605
329.2200580000	2.6832952207	-0.0962037187
329.6960640000	2.6623190374	-0.0979607657
330.1720700000	2.6404857174	-0.0995080205
330.6480760000	2.6178320217	-0.1008496635
331.1240820000	2.5943947413	-0.1019903019
331.6000880000	2.5702106369	-0.1029349352
332.0760940000	2.5453163815	-0.1036889201
332.5521000000	2.5197485049	-0.1042579366
333.0281060000	2.4935433408	-0.1046479530
333.5041120000	2.4667369757	-0.1048651920
333.9801180000	2.4393652002	-0.1049160980
334.4561240000	2.4114634635	-0.1048073038
334.9321300000	2.3830668289	-0.1045455989
335.4081360000	2.3542099331	-0.1041378990
335.8841420000	2.3249269464	-0.1035912158
336.3601480000	2.2952515364	-0.1029126283
336.8361540000	2.2652168339	-0.1021092555

337.3121600000	2.2348554000	-0.1011882298
337.7881660000	2.2041991974	-0.1001566725
338.2641720000	2.1732795621	-0.0990216697
338.7401780000	2.1421271784	-0.0977902507
339.2161840000	2.1107720559	-0.0964693666
339.6921900000	2.0792435087	-0.0950658717
340.1681960000	2.0475701361	-0.0935865050
340.6442020000	2.0157798063	-0.0920378741
341.1202080000	1.9838996412	-0.0904264402
341.5962140000	1.9519560039	-0.0887585045
342.0722200000	1.9199744877	-0.0870401960
342.5482260000	1.8879799068	-0.0852774609
343.0242320000	1.8559962889	-0.0834760529
343.5002380000	1.8240468698	-0.0816415252
343.9762440000	1.7921540893	-0.0797792236
344.4522500000	1.7603395885	-0.0778942809
344.9282560000	1.7286242091	-0.0759916123
345.4042620000	1.6970279939	-0.0740759119
345.8802680000	1.6655701885	-0.0721516507
346.3562740000	1.6342692447	-0.0702230748
346.8322800000	1.6031428249	-0.0682942054
347.3082860000	1.5722078074	-0.0663688389
347.7842920000	1.5414802935	-0.0644505486

348.2602980000	1.5109756152	-0.0625426865
348.7363040000	1.4807083437	-0.0606483860
349.2123100000	1.4506922994	-0.0587705656
349.6883160000	1.4209405620	-0.0569119326
350.1643220000	1.3914654825	-0.0550749877
350.6403280000	1.3622786943	-0.0532620303
351.1163340000	1.3333911270	-0.0514751634
351.5923400000	1.3048130187	-0.0497162998
352.0683460000	1.2765539309	-0.0479871680
352.5443520000	1.2486227621	-0.0462893187
353.0203580000	1.2210277631	-0.0446241314
353.4963640000	1.1937765523	-0.0429928209
353.9723700000	1.1668761312	-0.0413964448
354.4483760000	1.1403329003	-0.0398359100
354.9243820000	1.1141526754	-0.0383119799
355.4003880000	1.0883407041	-0.0368252817
355.8763940000	1.0629016823	-0.0353763135
356.3524000000	1.0378397709	-0.0339654512
356.8284060000	1.0131586128	-0.0325929555
357.3044120000	0.9888613496	-0.0312589790
357.7804180000	0.9649506385	-0.0299635733
358.2564240000	0.9414286694	-0.0287066949
358.7324300000	0.9182971818	-0.0274882127

359.2084360000	0.8955574812	-0.0263079137
359.6844420000	0.8732104564	-0.0251655095
360.1604480000	0.8512565955	-0.0240606426
360.6364540000	0.8296960027	-0.0229928917
361.1124600000	0.8085284143	-0.0219617776
361.5884660000	0.7877532150	-0.0209667690
362.0644720000	0.7673694538	-0.0200072869
362.5404780000	0.7473758593	-0.0190827104
363.0164840000	0.7277708552	-0.0181923809
363.4924900000	0.7085525755	-0.0173356068
363.9684960000	0.6897188795	-0.0165116680
364.4445020000	0.6712673659	-0.0157198198
364.9205080000	0.6531953872	-0.0149592966
365.3965140000	0.6355000640	-0.0142293159
365.8725200000	0.6181782982	-0.0135290815
366.3485260000	0.6012267861	-0.0128577867
366.8245320000	0.5846420322	-0.0122146172
367.3005380000	0.5684203608	-0.0115987543
367.7765440000	0.5525579288	-0.0110093769
368.2525500000	0.5370507378	-0.0104456645
368.7285560000	0.5218946451	-0.0099067988
369.2045620000	0.5070853753	-0.0093919662
369.6805680000	0.4926185311	-0.0089003596

370.1565740000	0.4784896038	-0.0084311797
370.6325800000	0.4646939833	-0.0079836371
371.1085860000	0.4512269681	-0.0075569531
371.5845920000	0.4380837748	-0.0071503616
372.0605980000	0.4252595467	-0.0067631095
372.5366040000	0.4127493634	-0.0063944581
373.0126100000	0.4005482484	-0.0060436840
373.4886160000	0.3886511775	-0.0057100796
373.9646220000	0.3770530869	-0.0053929535
374.4406280000	0.3657488798	-0.0050916318
374.9166340000	0.3547334343	-0.0048054577
375.3926400000	0.3440016096	-0.0045337925
375.8686460000	0.3335482530	-0.0042760151
376.3446520000	0.3233682054	-0.0040315230
376.8206580000	0.3134563076	-0.0037997319
377.2966640000	0.3038074059	-0.0035800758
377.7726700000	0.2944163571	-0.0033720072
378.2486760000	0.2852780337	-0.0031749967
378.7246820000	0.2763873284	-0.0029885333
379.2006880000	0.2677391588	-0.0028121237
379.6766940000	0.2593284716	-0.0026452926
380.1527000000	0.2511502463	-0.0024875822
380.6287060000	0.2431994992	-0.0023385519

381.1047120000	0.2354712866	-0.0021977783
381.5807180000	0.2279607082	-0.0020648543
382.0567240000	0.2206629100	-0.0019393893
382.5327300000	0.2135730874	-0.0018210087
383.0087360000	0.2066864873	-0.0017093533
383.4847420000	0.1999984111	-0.0016040790
383.9607480000	0.1935042162	-0.0015048566
384.4367540000	0.1871993186	-0.0014113711
384.9127600000	0.1810791946	-0.0013233216
385.3887660000	0.1751393824	-0.0012404203
385.8647720000	0.1693754834	-0.0011623929
386.3407780000	0.1637831639	-0.0010889774
386.8167840000	0.1583581562	-0.0010199242
387.2927900000	0.1530962596	-0.0009549953
387.7687960000	0.1479933413	-0.0008939641
388.2448020000	0.1430453370	-0.0008366150
388.7208080000	0.1382482521	-0.0007827429
389.1968140000	0.1335981617	-0.0007321528
389.6728200000	0.1290912112	-0.0006846594
390.1488260000	0.1247236168	-0.0006400867
390.6248320000	0.1204916653	-0.0005982678
391.1008380000	0.1163917148	-0.0005590442
391.5768440000	0.1124201940	-0.0005222656

392.0528500000	0.1085736028	-0.0004877897
392.5288560000	0.1048485117	-0.0004554816
393.0048620000	0.1012415620	-0.0004252136
393.4808680000	0.0977494650	-0.0003968648
393.9568740000	0.0943690020	-0.0003703209
394.4328800000	0.0910970238	-0.0003454739
394.9088860000	0.0879304502	-0.0003222215
395.3848920000	0.0848662693	-0.0003004673
395.8608980000	0.0819015373	-0.0002801201
396.3369040000	0.0790333775	-0.0002610939
396.8129100000	0.0762589795	-0.0002433075
397.2889160000	0.0735755992	-0.0002266845
397.7649220000	0.0709805570	-0.0002111526
398.2409280000	0.0684712380	-0.0001966439
398.7169340000	0.0660450904	-0.0001830943
399.1929400000	0.0636996251	-0.0001704435
399.6689460000	0.0614324148	-0.0001586347
400.1449520000	0.0592410927	-0.0001476146
400.6209580000	0.0571233520	-0.0001373328
401.0969640000	0.0550769449	-0.0001277422
401.5729700000	0.0530996814	-0.0001187984
402.0489760000	0.0511894286	-0.0001104596
402.5249820000	0.0493441094	-0.0001026867

403.0009880000	0.0475617019	-0.0000954429
403.4769940000	0.0458402382	-0.0000886937
403.9530000000	0.0441778033	-0.0000824066
